# The Evolution of Hemocyanin Genes in Caenogastropoda: Gene Duplications and Intron Accumulation in Highly Diverse Gastropods

**DOI:** 10.1007/s00239-021-10036-y

**Published:** 2021-11-10

**Authors:** Gabriela Giannina Schäfer, Lukas Jörg Grebe, Robin Schinkel, Bernhard Lieb

**Affiliations:** Institute of Molecular Physiology, Johannes Gutenberg-University of Mainz, Johann-Joachim-Becher-Weg 7, 55128 Mainz, Germany

**Keywords:** Hemocyanin, Adaptation, Gene structure, Intron accumulation, Gene duplication, Caenogastropoda

## Abstract

Hemocyanin is the oxygen transport protein of most molluscs and represents an important physiological factor that has to be well-adapted to their environments because of the strong influences of abiotic factors on its oxygen affinity. Multiple independent gene duplications and intron gains have been reported for hemocyanin genes of Tectipleura (Heterobranchia) and the caenogastropod species *Pomacea canaliculata,* which contrast with the uniform gene architectures of hemocyanins in Vetigastropoda. The goal of this study was to analyze hemocyanin gene evolution within the diverse group of Caenogastropoda in more detail. Our findings reveal multiple gene duplications and intron gains and imply that these represent general features of Apogastropoda hemocyanins. Whereas hemocyanin exon–intron structures are identical within different Tectipleura lineages, they differ strongly within Caenogastropoda among phylogenetic groups as well as between paralogous hemocyanin genes of the same species. Thus, intron accumulation took place more gradually within Caenogastropoda but finally led to a similar consequence, namely, a multitude of introns. Since both phenomena occurred independently within Heterobranchia and Caenogastropoda, the results support the hypothesis that introns may contribute to adaptive radiation by offering new opportunities for genetic variability (multiple paralogs that may evolve differently) and regulation (multiple introns). Our study indicates that adaptation of hemocyanin genes may be one of several factors that contributed to the evolution of the large diversity of Apogastropoda. While questions remain, this hypothesis is presented as a starting point for the further study of hemocyanin genes and possible correlations between hemocyanin diversity and adaptive radiation.

**Supplementary Information:**

The online version contains supplementary material available at 10.1007/s00239-021-10036-y.

## Background

Mollusca is the second largest animal phylum and includes over 82,000 extant species (for numbers cf. WoRMS Editorial Board 2020). The great diversity of this phylum is represented best by the two large gastropod groups Heterobranchia and Caenogastropoda which together form the clade Apogastropoda. They comprise over 64,000 species living in various habitats including the sea, fresh waters and terrestrial ecosystems, as well as all kinds of intermediate environments. The numerous habitat shifts, which were undergone multiple times independently by different groups of Apogastropoda, were enabled by a multitude of adaptations that resulted in enormous diversification. In addition to the evolution of a range of different lifestyles and morphological adaptations, modifications of respiratory systems have been essential during habitat shifts. In addition to the evolution of new respiratory organs such as pneumostomes and lungs (Dayrat and Tillier 2002; Jörger et al. 2010; Kocot et al. 2013; Schrödl 2014), molecular adaptations that influence respiration have been detected, e.g., adaptations of mitochondrial complexes of Panpulmonata to increase metabolic efficiency (Romero et al. 2016b) or the evolution of multiple metabolic states using different levels of available oxygen (Schweizer et al. 2019).

Another very important factor of gastropod respiration that has to be adapted to environmental conditions is the oxygen transporter hemocyanin. Previous studies have shown that oxygen affinity, which strongly influences the function of hemocyanin, is temperature dependent (Brix et al. 1989, 1995; Mangum 1990; Miller 1985; Miller and van Holde 1974). Thus, shifts to habitats with different temperatures must be accompanied by adaptations of these proteins to sustain a sufficient oxygen supply. In particular, environments with varying temperatures, e.g., land and intertidal zones in contrast to solely marine habitats, require well-adapted oxygen transport proteins. Different hemocyanin paralogs can have different oxygen affinities (Swerdlow et al. 1996), and differential expression helps to adapt to varying oxygen conditions (e.g., low oxygen pressure in eggs of *Sepia officinalis*; Gutowska and Melzner 2009; Strobel et al. 2012). We previously reported a multitude of hemocyanin gene duplications in different species of Tectipleura (Schäfer et al. 2018). This large group of Heterobranchia comprises very diverse snails that conquered land and freshwater several times independently in different lineages (Dinapoli and Klussmann-Kolb 2010; Jörger et al. 2010; Kano et al. 2016; Kocot et al. 2013; Romero et al. 2016a). Therefore, we hypothesized that hemocyanin duplications may have helped to increase genetic variability by leading to a multitude of hemocyanin paralogs with potentially different properties and/or varying expression patterns. Accordingly, they may represent one of many factors that have enabled the exploitation of new habitats and extremely large adaptive radiation (Schäfer et al. 2018).

**Fig. 1:**
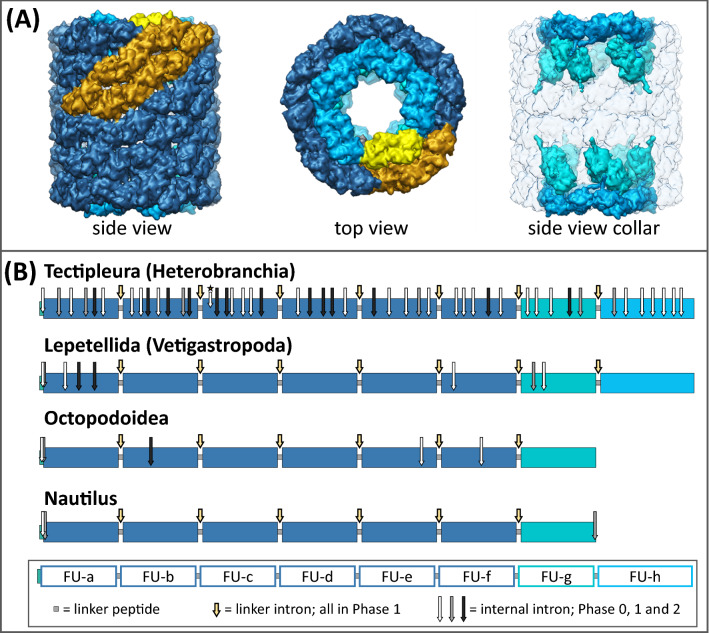
Molluscan hemocyanin: molecules and genes. **A** Typical gastropod hemocyanin didecamer and based on the 9 Å model of KLH1 (Gatsogiannis and Markl 2009, PDB: 4BED). The wall (FU-a–FU-f) is colored in dark blue. The collar is restricted to both sides of the didecamer and built by 10 FU-g (cyan) and 10 FU-h (light blue). Side and top views are depicted with one hemocyanin subunit dimer highlighted in gold (wall) and gray/yellow (collar: FU-g/h). **B** Exon–intron structure of molluscan hemocyanins. Shown are coding sequences of molluscan hemocyanins. Their genes typically contain ~ 10,200–10,300 nucleotides coding for the eight functional units (FU-a, FU-b, …, FU-h) of one hemocyanin polypeptide subunit. FU-h is approximately 300 nucleotides longer than the other functional units. Hemocyanin genes in Cephalopoda do not contain FU-h. Large boxes represent functional units (FU-a, FU-b, …, FU-h), and small boxes between them represent linker peptides. Arrows symbolize intron positions in hemocyanin genes with respect to the coding sequences. Linker introns are conserved in phase 1 within all known hemocyanin genes (yellow arrows). Internal introns occur in all intron phases and are color-coded accordingly: located before the first (phase 0, white), after the first (phase 1, gray) or after the second (phase 2, black) nucleotide of a codon. Hemocyanin genes in Tectipleura comprise a significantly larger number of internal introns than those in Lepetellida, Octopodoidea or Nautilus adapted from Schäfer et al. 2021a (Color figure online)

The overall shape of functional molluscan hemocyanin proteins is a partly hollow cylinder of 4 MDa formed by decamers of 35 nm in diameter which can assemble into di- or multidecamers (Fig. 1A). These large oxygen transport molecules float freely within the hemolymph of most molluscs (van Holde and Miller 1995). The basic structure of a single 400 kDa polypeptide subunit encompasses eight paralogous domains called functional units a, b, …, h (FU-a to FU-h), which are connected by short linker regions (Fig. 1B). The FUs have similar tertiary structures forming 45 to 50 kDa large globular substructures of the polypeptides and comprising one oxygen binding site each. Thus, one didecamer, which is the most common hemocyanin molecule in gastropods, encompasses 160 oxygen binding sites (basic structure reviewed in Markl 2013 and Kato et al. 2018). The basic composition of hemocyanin subunits, including multiple FU domains as well as the primary structures of these FUs are highly conserved across all different molluscan classes that have been analyzed thus far (overview in Markl 2013).

The segmentation of molluscan hemocyanin subunits in multiple FU domains is also represented by the highly conserved basic exon–intron structure of their genes (Lieb et al. 2001). Gene segments that encode for different functional units are separated by phase 1 introns (Fig. 1B; intron phases 0/1/2 are defined as being located before the first/after the first/after the second nucleotide of a codon). Within all FUs of molluscan hemocyanins that have been analyzed so far, they lie at almost equivalent positions just upstream of linker peptide coding regions (Altenhein et al. 2002; Bergmann et al. 2006; Lieb et al. 2001; Schäfer et al. 2021b). Accordingly, these introns are termed *linker introns*, while those lying within FU-coding regions are termed *internal introns*. The numbers and positions of internal introns are less conserved and differ between hemocyanins of different molluscan lineages (Fig. 1B). Previous studies showed that the number of these internal introns varies greatly between Octopodoidea (5 internal introns) or Lepetellida (Vetigastropoda; 8 internal introns) and Tectipleura (Heterobranchia; 46 internal introns) but are conserved within these different groups of molluscs (Altenhein et al. 2000, 2002; Lieb et al. 2001; Yao et al. 2019).

Despite the strong conservation of the cylindrical hemocyanin structure, their subunits and their genes, deviations from these basic structures have been described for several molluscan groups. These deviations mostly concern the number of functional units which changed due to domain duplications or losses, e.g., hemocyanins of Cephalopoda lack FU-h (van Holde and Miller 1995). While the basic structure of gastropod hemocyanins corresponds to the typical eight functional unit domains, multiple variations have been found for hemocyanins of Caenogastropoda and are discussed below.

Within the extremely large and diverse Cerithioidea (Cerithiida, Caenogastropoda), the so-called mega-hemocyanin has been identified (Lieb et al. 2010). It represents a hemocyanin tridecamer that includes two typical decamers built from 400 kDa subunits and additionally one larger decamer that is located between the two typical decamers. This larger decamer is composed of subunits with a molecular mass of 550 kDa. These 550 kDa mega-hemocyanin subunits lack FU-g and FU-h but encompass six additional functional units which are paralogous to FU-f (FU-f_1_, FU-f_2_ … FU-f_6_) (Gatsogiannis et al. 2015). These additional FUs reach within the center of the molecule and fill the mega-hemocyanin cylinders. Therefore, they increase the oxygen transport capacity. The viscosity and the colloid-osmotic pressure of the hemolymph, however, remain the same as in a typical hemocyanin tridecamer, thus, the oxygen transport efficiency is increased (Gatsogiannis et al. 2015). The ability to differentially express the 400 kDa and 550 kDa hemocyanin subunits most likely facilitates variable ratios of typical hemocyanins and mega-hemocyanins. This may further help to adapt to different living conditions and may have accelerated the adaptive radiation of the extremely diverse group of Cerithioidea (Lieb et al. 2010).

**Fig. 2:**
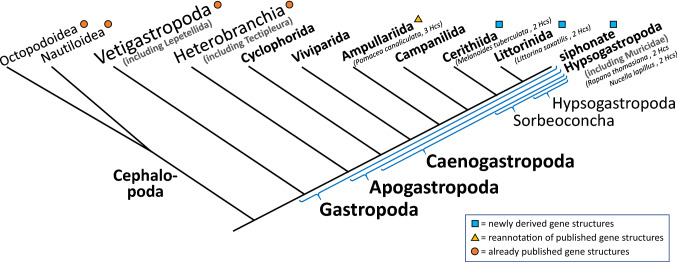
Broad systematics of Gastropoda focusing on Caenogastropoda. Despite a large number of phylogenetic studies on Caenogastropoda, many phylogenetic relationships within that large group of gastropods remain unresolved. The depicted systematics are combined from different studies by Ponder et al. (2019). Caenogastropoda species included in this study and their numbers of hemocyanin (Hc) paralogs are shown in brackets. Groups in which hemocyanin gene structures are newly derived in this study, reannotated or already published are indicated

Recently, we reported an additional variation in one of the two hemocyanin subunits of *Rapana venosa* (RtH2 derived from the synonym *R. thomasiana*) and *Nucella lapillus* (NlH2) (Schäfer et al. 2021a). Both species belong to Muricidae, which represents another main group of Caenogastropoda (Fig. 2). We identified 118 (in RtH2) and 340 (in NlH2) highly hydrophilic amino acids within the N-terminal region of FU-g in addition to the highly conserved amino acids within a typical molluscan hemocyanin. These additional amino acids seem to build an extra mass within the hemocyanin didecamer of up to 800 kDa and may influence the function of this hemocyanin molecule within these species (Schäfer et al. 2021a).

Chiumiento et al. (2020) analyzed hemocyanins of *Pomacea canaliculata*, a species that belongs to Ampullariida and represents a third main group of Caenogastropoda (Fig. 2). They identified four hemocyanin subunits that correspond to the basic polypeptide structure of this oxygen transporter but encompass a remarkably larger number of introns than hemocyanins of Vetigastropoda or Cephalopoda (27–32 in *P. canaliculata*, only 9–15 in Vetigastropoda or Cephalopoda; Altenhein et al. 2002; Bergmann et al. 2006; Lieb et al. 2001; Yao et al. 2019). Since hemocyanin genes of Tectipleura include 53 introns each (Schäfer et al. 2021b), large numbers of introns in hemocyanin genes may be a feature of Apogastropoda in general. According to Chiumiento et al. (2020), the exon–intron architectures of the four hemocyanin genes of *P. canaliculata* differ from each other. This contrasts with the exon–intron structures of hemocyanins that have been analyzed for Vetigastropoda (Lepetellida), Heterobranchia (Tectipleura) and Octopodoidea, which are highly conserved across different hemocyanin paralogs (Altenhein et al. 2002; Schäfer et al. 2021b).

Motivated by the above exceptions in the organization of caenogastropod hemocyanins, we analyzed the evolution of the genes coding for this oxygen transporter within Caenogastropoda in more detail. Therefore, we inferred the evolutionary background of caenogastropod hemocyanin genes by reconstructing phylogenetic trees and compared their exon–intron structures. These results revealed a detailed, novel scenario of intron evolution in gastropod hemocyanins. Therefore, our analysis included the previously described hemocyanins of the Cerithioidea *Melanoides tuberculata* [MtH_400_ and MtH_550_ (Lieb et al. 2010), the Muricidae species *R. venosa* and *N. lapillus* (RtH and NlH; Gebauer et al. 1999; Schäfer et al. 2021b) and three hemocyanins of *P. canaliculata* (Ampullariida; Chiumiento et al. 2020)]. Additionally, we characterized hemocyanins from *Littorina saxatilis*, a species that belongs to the same large group of Hypsogastropoda as the Muricidae *R. venosa* and *N. lapillus* but does not belong to the siphonate clade (Fig. 2; Ponder et al. 2019).

## Methods

### Animal Sampling and DNA Isolation

One individual of *M. tuberculata* was taken from a freshwater aquarium at the Institute of Molecular Physiology in Mainz. Three specimens of *N. lapillus* were collected at the western Atlantic coast of Brittany, France (Schäfer et al. 2021a). DNA of one individual of both species was isolated from foot tissue using the E.Z.N.A.® Mollusc DNA Kit (Omega Bio-Tek, Norcross, GA, USA). Via a Nanodrop (Thermo Fisher Scientific, Waltham, MA, USA), the DNA was checked for purity and quantified. Subsequently, the DNA was sent to StarSeq in Mainz, Germany, for next-generation sequencing (NGS, Illumina Next Seq500) and library preparation to subsequently enable the reconstruction of hemocyanin gene structures (see below).

### In Silico Assembly of Hemocyanin cDNAs of *L. saxatilis* and *P. canaliculata*

Hemocyanin cDNA sequence assemblies were performed with Geneious 9.1.8 (Kearse et al. 2012) using publicly available transcriptomic raw data of *L. saxatilis* (SRR9651721, SRR9651722, SRR9651724) and *P. canaliculata* (SRR6429145, SRR6429146, SRR6429153) to obtain hemocyanin coding sequences. Paired-end reads were set, sequencing adapters were removed, and transcriptomic raw reads were quality trimmed with Geneious 9.1.8 (Kearse et al. 2012). Processed reads of *L. saxatilis* were mapped to the previously published cDNA sequence of the 400 kDa hemocyanin of *M. tuberculata* (KC405575, overlap identity: 70%). Those of *P. canaliculata* were mapped to the cDNA sequences that we deduced from the published hemocyanin gene structures (Chiumiento et al. 2020). Reads that mapped against the known references were used as references for iterative mappings of the remaining reads to elongate cDNA fragments and to obtain the full-length coding sequences [minimum overlap: 60 nucleotides; minimum overlap identity: 99%; maximum mismatches: 1%; using Geneious 9.1.8 (Kearse et al. 2012)]. This mapping process was reiterated until the isolated fragments resulted in complete hemocyanin coding sequences.

The existence of multiple hemocyanin genes per species and the repetitive structure of their cDNAs, which contain sequences coding for functional units that share some highly conserved amino acid motifs, may challenge correct assemblies. To preclude such misassemblies, we verified the sequences by (i) low sensitive mappings that enable misassembly detection, (ii) analyzing the sequences for highly identical sections between hemocyanin sequences of a species to enable manual double checking for correct assemblies and (iii) using paired-end reads. For a more detailed description of sequence assembly and verification, see Schäfer et al. 2021a.

### Reconstructing Exon–Intron Structures of Hemocyanin Genes

For the reconstruction of gene architectures, we used Geneious 9.1.8 as a bioinformatic tool (Kearse et al. 2012) to map genomic NGS data to hemocyanin coding sequences. For *M. tuberculata* and *N. *lapillus, we used NGS data sequenced by StarSeq in Mainz, Germany (see above), which included 104,512,762 and 195,550,720 sequences, respectively. Public genomic NGS data were used for *R. venosa* (SRR5371534; 661,123,146 sequences) and *L. saxatilis* (SRR7976330; 502,027,256 sequences). All genomic NGS data were processed as described for transcriptomic raw reads. Trimmed and paired-end reads were then mapped to coding sequences of *R. venosa* (BK014286, BK014287), *N. lapillus* (MT939254, MT939255), *M. tuberculata* (KC405575, KC405576) and *L. saxatilis* (obtained in this study, BK014376, BK014375). The mapping results showed that some parts of the cDNA sequences were not covered by genomic NGS data or showed inconsistencies. These sections were used to subdivide the cDNA sequences into different sections representing hypothetical exons. To obtain all splice sites, these sections were extended by repetitive mappings of genomic NGS data until their 3’ and 5’ ends deviated by at least ten base pairs from cDNA sequences (procedure explained in more detail in Schäfer et al. 2021b). In this way, we derived intron positions within hemocyanin genes. Therefore, our analyses did not include characterization of intron lengths or sequences. The corrected cDNA sequences of *P. canaliculata* (BK014379, BK014378, BK014377) were compared with genomic sequences by Chiumiento et al. (2020; cf. https://doi.org/10.5061/dryad.15nd8v3) to deduce splice site positions.

### Sequence Alignment and Phylogenetic Tree Generation

We used MEGA 7 (Kumar et al. 2016) to align amino acid sequences by applying the implemented Muscle algorithm and to determine LG + G + I + F as the best evolutionary model. This model was used to compute the maximum likelihood tree with branch supports based on 100 bootstrap replicates using MEGA version 7 (Kumar et al. 2016). Hemocyanin sequences of the Cephalopoda *Nautilus pompilius* and *Enteroctopus dofleini* were used as outgroups. We used MrBayes 3.2.6 (Huelsenbeck and Ronquist 2001), which is implemented in Geneious 9.1.8 (Kearse et al. 2012), to conduct Bayesian inference based on two parallel runs of four Monte Carlo Markov chains (MCMC) with one million generations, a subsampling frequency of 500 and a burn-in of 250,000.

## Results

To reveal relationships between different hemocyanin paralogs and to identify hemocyanin gene duplication events within Caenogastropoda, we inferred the phylogeny of these proteins, including eleven hemocyanin sequences of over 10,000 nucleotides each from species that belong to four different main groups of Caenogastropoda. To further investigate the evolution of hemocyanin genes in Caenogastropoda, we compiled exon–intron structures for all eleven caenogastropod hemocyanin genes. These analyses include the previously published hemocyanin cDNA sequences of the Muricidae species (siphonate Hypsogastropoda) *N. lapillus* (two hemocyanin paralogs: MT939254, MT939255) and *R. venosa* (two hemocyanin paralogs: BK014286, BK014287) and of the Cerithioidea species *M. tuberculata* (two hemocyanin paralogs: KC405575, KC405576). Additionally, we revised the hemocyanin sequences of the Ampullariida species *P. canaliculata* (three hemocyanin paralogs: BK014379, BK014378, BK014377) and newly assembled the cDNA of the hemocyanins of *L. saxatilis* (two hemocyanin paralogs: BK014376, BK014375), which belongs to the asiphonate Hypsogastropoda (Fig. 2).

### Canonical Hemocyanin Coding Sequences Identified for *L. saxatilis* and *P. canaliculata*

We obtained two complete hemocyanin coding sequences for *L. saxatilis* by assembling public transcriptomic NGS data (LisaH1, LisaH2). For *P. canaliculata,* four hemocyanin cDNAs were published by Chiumiento et al. (2020). As previously reported, these sequences contained some inconsistencies (cf. Schäfer et al. 2021a). By assembling transcriptomic NGS data, we identified and corrected three of those hemocyanin cDNA sequences (PcH I, IIb, III). This facilitated comparative phylogenetic analyses and helped to elucidate the correct gene architectures (see below).

The obtained hemocyanin sequences of *L. saxatilis* and *P. canaliculata* include eight canonical FUs (a, b, …, h) and comprise approximately 10,250–10,300 nucleotides, as is typical for gastropod hemocyanins (cf. Lieb et al. 2000, 2004; Schäfer et al. 2018). Accession numbers, lengths and molecular weights for all hemocyanin cDNA sequences and primary structures of *L. saxatilis* and *P. canaliculata* are shown in Table 1.

**Table 1:** Hemocyanins of *L. saxatilis* (LisaH) and *P. canaliculata* (PcH)

	LisaH1	LisaH2	PcH I	PcH IIb	PcH III
Accession number	BK014376	BK014375	BK014379	BK014378	BK014377
CDS (nt)	10,308	10,278	10,278	10,287	10,272
Primary structure (aa)	3436	3426	3426	3429	3424
Deduced molecular mass (kDa)	392	391	391	391	390

Accession numbers; the lengths of coding sequences (CDS) in nucleotides (nt); the number of amino acids (aa) for the deduced primary structure of the polypeptides; and the calculated molecular weight in kDa are shown

### Phylogenetic Analyses Reveal Multiple Independent Gene Duplications in Different Caenogastropods

**Fig. 3:**
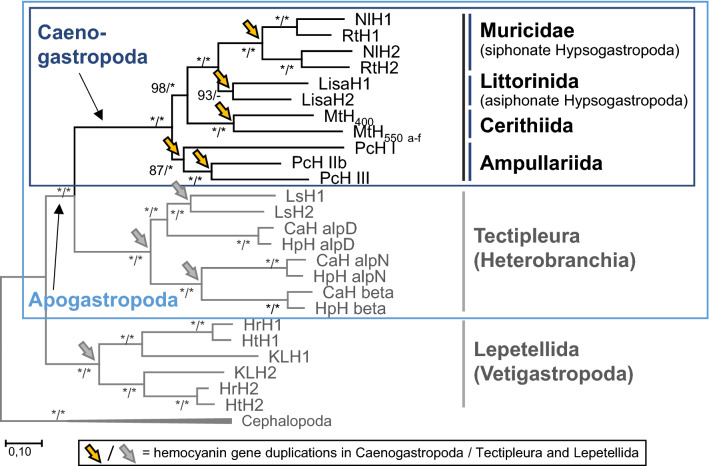
Maximum likelihood (ML) tree of gastropod hemocyanins. The phylogenetic tree is based on an amino acid sequence alignment and conducted with MUSCLE (Edgar 2004) implemented in MEGA7 (Kumar et al. 2016) using the LG + G + I + F model. Independent hemocyanin gene duplications (symbolized by orange arrows) occurred within all main groups of Caenogastropoda included in this study, namely, within Ampullariida, Cerithiida and Hypsogastropoda. Therefore, the tree includes hemocyanins from the following Caenogastropoda: *Pomacea canaliculata* (PcH I + IIb + III), *M. tuberculata* (MtH_400+550_), *Littorina saxatilis* (LisaH1 + 2), *Rapana venosa* (RtH1 + 2) and *Nucella lapillus* (NlH1 + 2). It further encompasses hemocyanins from Tectipleura (*Helix pomatia* HpHαD + αN + β; *Cornu aspersum* CaHαD + αN + β; *Lymnaea stagnalis* LsH1 + 2) and Lepetellida (*Haliotis tuberculata* HtH1 + 2; *Haliotis rubra* HrH1 + 2; *Megathura crenulata* KLH1 + 2). Gene duplications within hemocyanins from Tectipleura and Lepetellida are represented by gray arrows. The tree was rooted with hemocyanins of the Cephalopoda *Nautilus pompilius* (NpH) and *Enteroctopus dofleini* (OdH_A_ and OdH_G_). Nodes are congruent with those obtained by Bayesian inference except for the position of LisaH2, which is grouped together with hemocyanins of Muricidae in the Bayesian analysis tree (Supplement 1). Hemocyanin gene duplications in Caenogastropoda are not affected by the previously described deviations from maximum likelihood or Bayesian inference. Nodes are labeled with bootstrap (BS) percentages based on 100 replicates from ML analyses and posterior probabilities (PP) computed by MrBayes (BS/PP). Asterisks indicate nodes supported by BS ≥ 99%/PP ≥ 0.99

Molecular phylogenetic trees based on amino acid alignments of full-length protein sequences and inferred by maximum likelihood and Bayesian analyses are well-supported (Fig. 3, Supplement 1). These trees are largely congruent with each other and share the same nodes, except for minor differences in one hemocyanin from *L. saxatilis* (LisaH2), which is grouped with hemocyanins from Muricidae in the Bayesian analysis. The obtained phylogenies of caenogastropod hemocyanins are in accordance with the currently accepted systematic relationships of the four groups Muricidae, Littorinida, Cerithiida and Ampullariida (Fig. 2; Bouchet et al. 2017; Ponder et al. 2019). The phylogenies obtained by maximum likelihood and MrBayes both reveal that the multiple hemocyanin genes from different analyzed species resulted from independent gene duplications that occurred after the diversification of Caenogastropoda into Ampullariida, Cerithiida and Hypsogastropoda (orange arrows in Fig. 3). Although the position in the various phylogenetic trees of LisaH2 is uncertain within the hemocyanins of Hypsogastropoda, the results of maximum likelihood and Bayesian inferences both support multiple independent duplications. The gene duplication that led to the two hemocyanin paralogs of *R. venosa* and *N. lapillus* most likely took place in a common ancestor of both Muricidae species but after separation of siphonate and asiphonate Hypsogastropoda (Fig. 3).

### High Variability of Hemocyanin Gene Architectures in Caenogastropoda

**Fig. 4:**
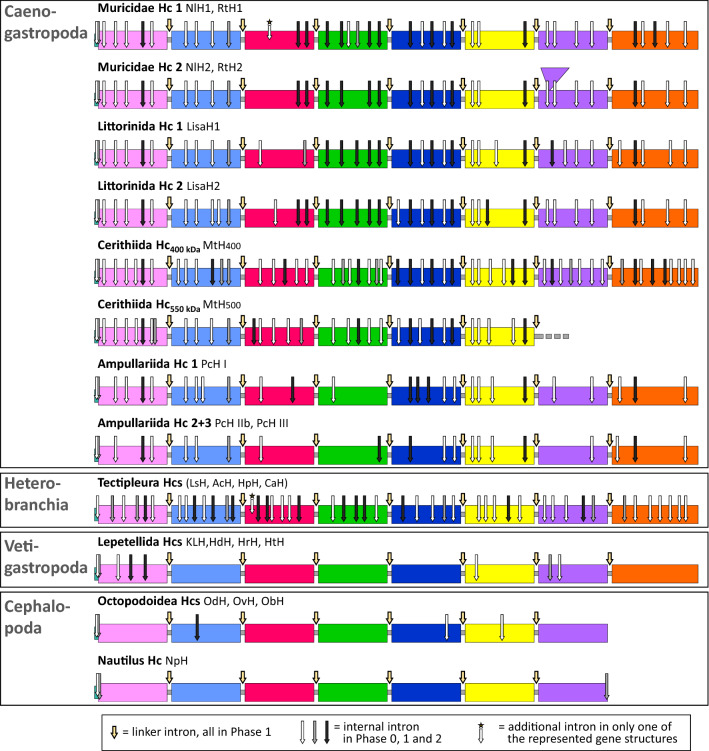
Hemocyanin gene structures of Caenogastropoda, Heterobranchia, Vetigastropoda and Cephalopoda. The comparison of exon–intron structures indicates that nine of the eleven analyzed hemocyanin genes in Caenogastropoda vary in their gene structures concerning the number and positions of internal introns. The coding sequences of hemocyanins (large boxes: functional units; small and gray boxes: linker peptides) are shown, and intron positions within their genes are represented by arrows (cf. alignment in Supplement 2). To enable the comparability of gene structures, we did not enlarge the box representing the FU-g coding sequence of Muricidae Hc2 despite 118 and 340 additional amino acids being identified for RtH2 and NlH2 (cf. Supplement 2, Schäfer et al. 2021a). Instead, we included a violet triangular box on top of the box representing the additional amino acids. Introns are divided into linker introns, which are highly conserved within all molluscan hemocyanins (bold yellow arrows), and internal introns, which differ between various genes (thin arrows in phase 0: white; phase 1: gray or phase 2: black). Smaller arrows with a star on top represent internal introns that were obtained in only one hemocyanin from one structure type (one intron within NlH1, Muricidae; and one within LsH1, Tectipleura). The comparison includes all of the hemocyanins analyzed in this study from the following Caenogastropoda species: *Nucella lapillus* (NlH1 + 2), *Rapana venosa* (RtH1 + 2), *Littorina saxatilis* (LisaH1 + 2), *Melanoides tuberculata* (MtH_400_; MtH_550_ FU-a–FU-f; exon–intron structures of the additional functional units f_1_, f_2_, …, f_6_ of the mega-hemocyanin of *M. tuberculata* are shown in Supplement 3) and *P. canaliculata* (PcH I + IIb + III). Additionally, conserved gene structures are included for hemocyanins of Tectipleura (*Lymnaea stagnalis* LsH, *Aplysia californica* AcH, *Helix pomatia* HpH and *Cornu aspersum*), Lepetellida (*Megathura crenulata* KLH, *Haliotis diversicolor* HdH, *Haliotis rubra* HrH, *Haliotis tuberculata* HtH), Octopodoidea (*Enteroctopus dofleini* OdH, *Octopus vulgaris* OvH and *O. bimaculoides* ObH) and *Nautilus pompilius* NpH (Color figure online)

Characterization of the gene structures of hemocyanins in five Caenogastropoda (each species has 2–3 hemocyanin paralogs; Table 2) revealed that nine of the eleven hemocyanin genes differ in both the number and relative intragene positions of internal introns (Fig. 4). Therefore, we obtained exon–intron architectures for hemocyanin genes from different caenogastropod lineages (variations between Muricidae, Littorinida, Cerithioidea and Ampullariida and between different hemocyanin paralogs within the same species, e.g., between RtH1 and RtH2 of *R. venosa* or LisaH1 and LisaH2 of *L. saxatilis*). Identical exon–intron structures are only present for hemocyanin gene 2 of the two Muricidae species (RtH2 and NlH2; RtH1 and NlH1 vary in one intron but differ strongly from RtH2 and NlH2) and for the hemocyanin genes PcH IIb and PcH III of *P. canaliculata*.

**Table 2:** FU-internal introns of caenogastropod hemocyanins

FU	Hc										
	RtH1	NlH1	RtH2/NlH2	LisaH1	LisaH2	MtH_400_	MtH_550_	PcH I	PcH IIb/ PcH III	HpH	HtH
Sign	2	2	2	2	2	2	2	2	2	1	2
-a	5	5	5	5	5	6	7	4	3	5	3
-b	4	4	4	4	5	6	4	4	3	6	0
-c	2	3	2	2	3	5	5	2	1	6	0
-d	6	6	4	5	5	7	5	1	1	5	0
-e	5	5	5	5	6	6	6	5	3	5	0
-f	3	3	3	4	4	6	5	4	4	5	1
-g	4	4	4	4	4	7	–	2	1	5	2
-h	5	5	4	5	4	9	–	3	3	7	0
-f_1_	–	–	–	–	–	–	2	–	–	–	–
-f_2_	–	–	–	–	–	–	6	–	–	–	–
-f_3_	–	–	–	–	–	–	4	–	–	–	–
-f_4_	–	–	–	–	–	–	5	–	–	–	–
-f_5_	–	–	–	–	–	–	3	–	–	–	–
-f_6_	–	–	–	–	–	–	3	–	–	–	–
Σ(total, incl. sign.)	36	37	33	36	38	54	57	27	21	45	8
Σ(a–f)	25	26	23	25	28	36	32	20	15	32	4
Σ(FUs)	34	35	31	34	36	52	55	25	19	44	6
Ø Intron /FU	~ 4.3	~ 4.4	~ 3.9	~ 4.3	~ 4.5	~ 6.5	~ 4.6	~ 3.1	~ 2.4	~ 5.5	~ 0.8

Internal introns vary between hemocyanin genes of different Caenogastropoda groups and between different genes within the same caenogastropod species. These results contrast with the highly conserved exon–intron structures of Tectipleura (Heterobranchia) and Lepetellida (Vetigastropoda) represented in this table by the hemocyanins of *Helix pomatia* (HpH) and *Haliotis tuberculata* (HtH). The table shows the numbers of introns lying within the functional units of hemocyanins of *R. venosa* (RtH, two paralogs), *N. lapillus* (NlH, two paralogs), *L. saxatilis* (LisaH, two paralogs), *M. tuberculata* (MtH, two paralogs) and *P. canaliculata* (PcH, three paralogs). The numbers of internal introns are shown for the specific FUs and the signal peptide (sign.). Additionally, the table includes the sum of the internal introns of FU-a to FU-f (these FUs are included in all represented hemocyanins), as well as the total number of internal introns and the respective average number (Ø) of internal introns per functional unit in each hemocyanin

The total numbers of internal introns of caenogastropod hemocyanins vary between 21 and 57 (Table 2). The average numbers of internal introns per functional unit domain vary between 2.4 introns/FU (PcH IIb/III) and 6.5 introns/FU (MtH_400_). The comparison with known gastropod hemocyanin genes shows that all analyzed hemocyanins of Caenogastropoda contain a greater number of internal introns than those of Vetigastropoda (Altenhein et al. 2002; Lieb et al. 2001). Hemocyanins of Caenogastropoda contain fewer internal introns per functional unit than those of Tectipleura (cf. HpH in Table 2 and Fig. 4; Schäfer et al. 2021b), with the exception of MtH_400_ of *M. tuberculata*. Hemocyanin genes of this cerithioid encompass the greatest number of internal introns that have been identified thus far.

Linker introns are highly conserved across all molluscan hemocyanins that have been analyzed thus far (Bergmann et al. 2006; Lieb et al. 2001; Schäfer et al. 2021b) and are also present in hemocyanins of Caenogastropoda (Fig. 4). However, we did not detect an intron within the linker peptide-coding region between FU-f_1_ and FU-f_2_ of the mega-hemocyanin gene in *M. tuberculata* (star in Supplement 3). Thus, the MtH_550_ gene is not only the first mega-hemocyanin with a gene structure that has been analyzed but also the first hemocyanin gene that has been detected to lack a linker intron between two FU-coding regions. All other analyzed hemocyanin genes in Caenogastropoda include typical phase 1 linker introns between all FU-coding regions (Fig. 3, Supplement 2 + 3), which are characteristic of molluscan hemocyanins (Lieb et al. 2001).

## Discussion

Similar to most molluscs, Caenogastropoda use hemocyanin as an oxygen transporter. This respiratory protein has previously been identified within the Muricidae species *R. venosa* (Gebauer et al. 1999) and *N. lapillus* (Schäfer et al. 2021a), within the cerithioid *M. tuberculata* (Lieb et al. 2010) and within the apple snail *P. canaliculata* (Chiumiento et al. 2020). All of these Caenogastropoda species possess at least two hemocyanin genes like other Gastropoda (Gebauer et al. 1999; Markl et al. 1991; Schäfer et al. 2018). This gene system may enable differential expression of several hemocyanin genes as shown for Cephalopoda (Oellermann et al. 2015a, 2015b; Thonig et al. 2014). Similar regulatory mechanisms may help Caenogastropoda to adapt to different living conditions by sustaining oxygen supply despite changes in partial oxygen pressure and temperature, as hypothesized for Tectipleura (further discussed below under “Evolutionary constraints on hemocyanin genes in Apogastropoda?” and in Schäfer et al. 2018). by analyzing public transcriptomic NGS data, we corrected inconsistencies within published hemocyanin cDNA sequences from *P. canaliculata* and obtained two hemocyanins from *L. saxatilis,* which belongs to the asiphonate Hypsogastropoda and thus represent another lineage of the large group of Caenogastropoda (Fig. 2). Finally, we conducted molecular phylogenies based on maximum likelihood (Fig. 3; Felsenstein 1981; Kumar et al. 2016) and MrBayes (Supplement 1; Huelsenbeck and Ronquist 2001; Mau and Newton 1997) and analyzed the hemocyanin gene structures of the following species: *R. venosa, N. lapillus, L. saxatilis, M. tuberculata* and *P. canaliculata* (Fig. 4). Our analyses revealed ongoing hemocyanin gene evolution within all major groups of Caenogastropoda that were analyzed within this study: siphonate and asiphonate Hypsogastropoda (Muricidae and Littorinida), Cerithiida and Ampullariida (Fig. 2). Since we analyzed only one or two species per group and basal groups as Cyclophorida and Viviparida were not included in the study (Fig. 2), our analysis will not provide a comprehensive overview of hemocyanin gene evolution within Caenogastropoda. Nevertheless, by including species of various large groups of Caenogastropoda, our study gives a first insight into hemocyanin gene evolution within the diverse group of Caenogastropoda. Furthermore, the observed phenomenon is similar to that in their sister group Heterobranchia and thus will be discussed for the whole group of Apogastropoda.

### Multiple Independent Hemocyanin Gene Duplications: A Phenomenon of Apogastropoda

Both, maximum likelihood analysis and Bayesian phylogenetic inferences (Fig. 3, Supplement 1) revealed that multiple hemocyanin paralogs identified for *P. canaliculata*, *M. tuberculata*, *L. saxatilis* and the two Muricidae species *R. venosa* and *N. lapillus* resulted from independent gene duplications. This result is similar to the multiple gene duplications in Tectipleura (Heterobranchia), which took place independently in different groups (e.g., Stylommatophora, Hygrophila, Anaspidea; Schäfer et al. 2018) and thus may be a general phenomenon for Apogastropoda. These multiple independent gene duplications contrast with the much more conserved hemocyanin genes 1 and 2 of Lepetellida analyzed within *H. tuberculata*, *Haliotis diversicolor* and *Megathura crenulata* (Lieb and Markl 2004; Yao et al. 2019). These paralogous hemocyanin genes resulted from one gene duplication that took place before the split of Lepetellida into Haliotoidea and Fissurelloidea ~ 343 million years ago (Lieb and Markl 2004) and has been maintained in both lineages. Thus, the comparison of hemocyanin genes of Apogastropoda and Vetigastropoda indicates strongly different evolutionary dynamics of hemocyanin gene duplications during the evolution of these gastropod lineages.

### Extensive Variability of Hemocyanin Gene Structures of Caenogastropoda Suggests a Higher Continuous Intron Gain Rate than that Identified in any other Molluscs

In addition to multiple independent gene duplications, our results on exon–intron architectures also reveal a high rate of evolutionary changes in hemocyanin genes in Caenogastropoda. We have previously reported that hemocyanin genes of Tectipleura encompass a significantly larger number of FU-internal introns (on average 5.6 introns per FU; Schäfer et al. 2021b) than hemocyanin genes of Vetigastropoda or Cephalopoda (≤ 0.8 introns per FU). This study also identified a larger number of internal introns in hemocyanin genes of Caenogastropoda (2.4–6.5 introns per FU), the other branch of Apogastropoda (Fig. 4; Table 2). As typical for internal introns of molluscan hemocyanins, they vary in phases. Across all hemocyanin gene structures of Caenogastropoda that we have analyzed, phase 0 was the most frequent intron phase (≥ 50%). This result matches the results of Long & Deutsch (1999) and Fedorov et al. (1992) as well as the results for Tectipleura hemocyanins (Schäfer et al. 2021b). Intron phases affect which exons may be spliced alternatively. Since we did not detect any splice variants, we did not focus on analyzing these intron phases in more detail. However, the color-coded phases in Fig. 4 accentuate the differences, which will be described below. The distinct hemocyanin genes of Caenogastropoda differ strongly from each other in terms of the number and position of internal introns. This phenomenon applies to hemocyanins of different caenogastropod lineages (Muricidae, Littorinida, Cerithioidea and Ampullariida) as well as to paralogous genes within the same species (Fig. 4, Table 2). Large variations in gene architectures between paralogous hemocyanin genes within one molluscan species have not been reported before. To date, only for Hygrophila, a group of Tectipleura, have two paralogous hemocyanin genes been identified that vary in one of 45 or 46 introns. Specifically, hemocyanin gene 1 from *Lymnaea stagnalis*, *Radix balthica* and *Biomphalaria glabrata* (LsH1, RbH1, and BgHcl-2, which are all orthologous to each other) has one additional intron along with those conserved within all other analyzed Tectipleura hemocyanins, including hemocyanin gene 2 of those Hygrophila species (Fig. 4, arrow with star and Schäfer et al. 2021b).

**Fig. 5:**
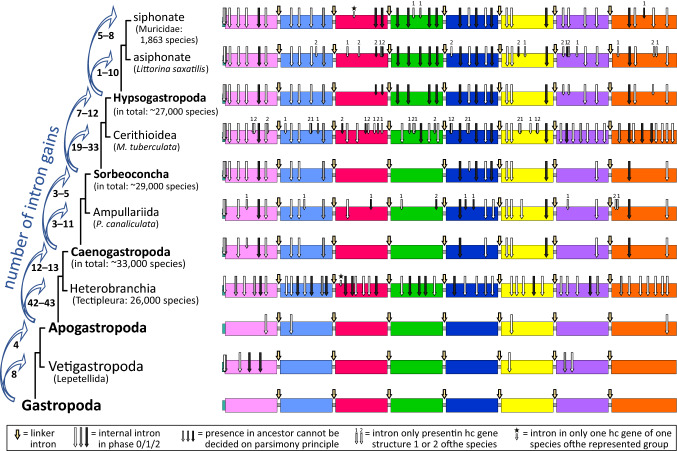
Maximum parsimony scenario of exon–intron architecture evolution within gastropod hemocyanins. The phylogenetic tree on the left shows the relation of gastropod species with known hemocyanin gene architectures (Ponder et al. 2019). For groups of Apogastropoda, the number of included species according to the WoRMS Editorial Board (2020) is also shown. If gene structures are known for only one representative of a lineage, the specific species is given. On the right, hypothetical gene structures are shown for the common ancestors. These structures are based on the maximum parsimony principle, assuming that introns that are at the same position concerning the coding sequences of hemocyanin genes of sister groups were already present in hemocyanin genes of a common ancestor. The same applies for intron losses if both descendants do not include a formerly present intron. The deduced model of gene structure evolution within gastropods shows a gradual gain of introns during the evolution of Caenogastropoda. The level of intron gains varies between the different hemocyanin lineages and is numbered on the left side of the taxonomic tree. If the exact number of intron gains is uncertain, we indicated the range between the possible minimum and maximum. We were unable to exactly assess the origin of every intron because two independent intron gains within two descendant species represent the same number of evolutionary events as one gain within a common ancestor together with an intron loss within one gene of one of the descendants. We highlighted these cases with smaller arrows in the ancestor genes. In contrast to the hemocyanin gene structures in Tectipleura, those in Caenogastropoda vary not only between different caenogastropod lineages but also within paralogous genes within the same species. We symbolized such introns that are present in the gene structure of only one hemocyanin gene of the same species with small arrows with the number of the gene in which it is located on top. Arrows with a star on top represent deviations from a conserved group-specific hemocyanin gene structure [e.g., additional Hygrophila-specific intron, see Fig. 4 and cf. Schäfer et al. (2018)]. As in all other molluscan hemocyanins, linker introns are located at the same position within the sequences coding for linker peptides between all canonical functional units

To analyze the evolution of hemocyanin gene structures of Gastropoda in more detail, we derived the most parsimonious scenario of intron evolution within these genes (Fig. 5). This scenario is based on the parsimony principle (Rogozin et al. 2006), which assumes that an intron shared within a sister group was already present within their common ancestor. The same applies to missing introns detected within sister groups that are assumed to be lost in an ancestor. Intron sliding was not considered because this phenomenon is difficult to identify based on location alone if introns vary by more than one nucleotide (Rogozin et al. 2000). Positions of nearby FU-internal introns within the analyzed hemocyanin genes of Caenogastropoda vary by at least six nucleotides (Supplement 2). Furthermore, intron sliding most likely contributes little to gene structure evolution (Poverennaya et al. 2020; Stoltzfus et al. 1997). It should be noted that the presence of some introns within an ancestor cannot be assessed because the two possible scenarios would have taken the same number of evolutionary steps (smaller arrows in a hypothetical gene precursor, Fig. 5). Nevertheless, this model of intron evolution shows the most parsimonious explanation for the revealed exon–intron structures and clearly indicates that ongoing changes within hemocyanin gene structures during the evolution of different Caenogastropoda are most likely. This scenario strongly suggests a gradual accumulation of introns, which led to gene structures with several internal introns, regardless of the exact origins of particular introns. Thus, our findings support the hypothesis that the accumulation of introns is a general phenomenon within hemocyanin genes of Apogastropoda and contrasts with the hemocyanin gene evolution of Vetigastropoda and Cephalopoda (Chiumiento et al. 2020; Schäfer et al. 2021b).

Considered more closely, the presented model of intron evolution reveals four internal introns that are present within all analyzed species of Tectipleura (Heterobranchia) and Caenogastropoda and thus within both major groups of Apogastropoda. Therefore, intron accumulation most likely started within a hemocyanin gene of a common ancestor, and ongoing accumulation subsequently led to various gene structures within different Apogastropoda lineages. The model of intron evolution shows, for example, that 12 or 13 FU-internal introns and two introns within the signal peptide-coding sequences are likely to have evolved within a common ancestor of the analyzed Caenogastropoda groups and that accumulation most likely continued gradually throughout the evolution of Sorbeoconcha, Hypsogastropoda and Muricidae (Fig. 5).

We have previously shown that the high intron gain rate is specific to the hemocyanin gene within Apogastropoda (Schäfer et al. 2021b). Such lineage- and gene-specific evolutionary rates have also been described for other genes (Carmel et al. 2007; Rogozin et al. 2003). A large number of introns may be accompanied by regulatory advantages, which may cause this lineage- and gene-specific trend of intron accumulation in hemocyanins in Apogastropoda (Schäfer et al. 2021b).

The extensive variation of exon–intron structures of caenogastropod hemocyanin genes reported in this study contrasts with the highly conserved exon–intron structures that have been detected in hemocyanins in Tectipleura (Heterobranchia). Hemocyanin gene architectures in Tectipleura evolved most likely within a common ancestor and thus before the multiple gene duplications that occurred in several Tectipleura groups. Subsequently, the exon–intron structure has remained mostly conserved within all analyzed species of this large group of gastropods (Schäfer et al. 2021b). Hemocyanin gene structures of Caenogastropoda, in contrast, changed independently within the different lineages and within different paralogous genes.

Since the number of Tectipleura species corresponds to ~ 80% of the number of Caenogastropoda species and even ~ 96% of the number of Hypsogastropoda species (Fig. 5; WoRMS Editorial Board 2020), the higher number of differences within hemocyanin genes in Caenogastropoda cannot be explained by the relatively higher number of species. Rather, we suggest that the intron gain rate decreased within Tectipleura quite early in evolution because their hemocyanin genes became saturated by introns more quickly than those of Caenogastropoda. Therefore, the large number of introns led to relatively small exon lengths, which may prevent further intron gains (Hawkins 1988; Hwang and Cohen 1997; Roy and Irimia 2009). Gene- and lineage-specific saturation of intron densities have already been described for plants (Basu et al. 2008) and mammals (Kordiš 2011). According to the most parsimonious scenario of intron evolution in gastropod hemocyanins (Fig. 5), 42 introns were gained during the evolution from an Apogastropoda ancestor to a precursor of Tectipleura and may have saturated the genes. This number is strikingly higher than that for intron gains during the evolution from an Apogastropoda ancestor to the ancestors of Caenogastropoda (12–13 gains), Sorbeoconcha (15–18 gains) or Hypsogastropoda (22–30 gains, Fig. 5). Schäfer et al. (2021b) identified conservation in the exon lengths within hemocyanin genes in Tectipleura. This phenomenon has been suggested to indicate evolutionary advantages (Davila-Velderrain et al. 2014; Fu and Lin 2012). Accordingly, our results showing conserved exon–intron structures may imply that the large number of introns and the regular distribution of exons within hemocyanin genes of Tectipleura may have evolutionary benefits, e.g., expanded possibilities of gene expression regulation (Schäfer et al. 2021b). More elaborate explanations of possible evolutionary advantages are further discussed below under “Evolutionary constraints on hemocyanin genes in Apogastropoda?”. If a similar evolutionary constraint acts on hemocyanin genes in Caenogastropoda, this might explain the continuous accumulation of introns in these genes during the evolution of Caenogastropoda, as proposed in the maximum parsimony scenario (Fig. 5). The hemocyanin gene architectures suggested for the common ancestors of Caenogastropoda, Sorbeoconcha or Hypsogastropoda include fewer introns than the gene structures of Tectipleura hemocyanins (Fig. 5). Therefore, such hemocyanin genes comprise relatively large exons which may represent possible targets for intron insertion (Hawkins 1988; Hwang and Cohen 1997; Roy and Irimia 2009). Due to lineage- and gene-specific intron gain/loss rates that may result from evolutionary constraints, the gene architectures of many hemocyanin genes may have accumulated introns during the evolution of Caenogastropoda until saturation is reached. This may be the reason for the continuous intron gains in hemocyanins in Caenogastropoda which contrasts with the conserved gene structure of Tectipleura hemocyanins. Although the rate of intron gains varies, intron accumulation seems to be common within hemocyanins of both groups of Apogastropoda and may be caused by evolutionary constraints.

### Evolutionary Constraints on Hemocyanin Genes in Apogastropoda?

Our results on the large number of gene duplications and the various intron gains identified for hemocyanin genes in Caenogastropoda support the hypothesis of high dynamics within hemocyanin gene evolution in Apogastropoda (Schäfer et al. 2018, 2021b). Both Caenogastropoda and Heterobranchia represent the most diverse groups of the phylum Mollusca and together encompass over 64,000 extant species (WoRMS Editorial Board 2020) that live in nearly all kinds of habitats from the deep sea to deserts. The radiation that led to the high diversity of Apogastropoda involved a great number of adaptations, including the evolution of altered abilities for osmoregulation, novel respiratory organs (pneumostomes and lungs) and reproductive behavioral strategies (Vermeij and Dudley 2000; Vermeij and Wesselingh 2002). As we have proposed previously, high evolutionary rates within hemocyanin genes may represent molecular adaptations that have enabled multiple habitat shifts and species diversification within Apogastropoda (Schäfer et al. 2018, 2021b). This hypothesis is developed from consideration of the strong temperature dependence of the oxygen affinity of hemocyanins (Brix et al. 1989; Burnett et al. 1988; Mangum 1990). Various adaptations of this oxygen transport protein to different environmental conditions have been reported (González et al. 2017; Melzner et al. 2007; Oellermann et al. 2015a, b; Strobel et al. 2012; Yesilyurt et al. 2008; Zielinski et al. 2001). These adaptations appear necessary to ensure a sufficient supply of oxygen and therefore are fundamental for molluscs to survive. Consequently, they represent one essential precondition for habitat shifts between strongly different environments (e.g., from sea to land or freshwater). Strong variability, as previously identified for hemocyanin genes in Tectipleura and now verified for five groups of Caenogastropoda, may accommodate these required adaptations (Schäfer et al. 2018, 2021b).

Gene duplications, as we have identified for hemocyanins in Tectipleura and Caenogastropoda (Fig. 3), play a major role in genomic complexity and evolution (Magadum et al. 2013; Ohno 1970). They are a driving force in organismal diversity (Lynch and Conery 2000) and can promote adaptation (Qian and Zhang 2014). Hemocyanin gene duplications could be followed by differential evolution of various genes and could eventually lead to hemocyanins with, for example, different oxygen affinities, varying pH or temperature sensitivities or differential expression patterns. These differences may represent the origin of genetic variability and adaptation, as has already been discovered for Cephalopoda (Oellermann et al. 2015a, b; Strobel et al. 2012; Thonig et al. 2014). The squid *S. officinalis*, for example, possesses multiple hemocyanin genes that underlie differential expression (Strobel et al. 2012; Thonig et al. 2014). Thonig et al. (2014) identified ontogeny-specific expression patterns of hemocyanin genes in this squid species. For example, one hemocyanin gene is highly expressed within the egg and may help to sustain an adequate oxygen supply despite hypoxic and hypercapnic conditions arising within the eggs during embryogenesis. After hatching, however, the expression of this gene is downregulated, whereas the expression of two other hemocyanin genes is upregulated (Thonig et al. 2014). To examine whether the multitude of hemocyanin genes in Apogastropoda contributes to adaptive radiation and evolutionary benefits or if it has even led to neofunctionalization of these proteins, further studies are needed to examine the biochemical properties and physiological implications of paralogous hemocyanins in Heterobranchia and Caenogastropoda.

In addition to gene duplications, our findings confirm that intron accumulation is a general phenomenon of hemocyanin genes in Apogastropoda (Chiumiento et al. 2020; Schäfer et al. 2021b). As already proposed, this result may indicate evolutionary constraints on a large number of introns in gene structures of Apogastropoda. Introns, in general, can increase species diversity (Calarco and Ellis 2020) and thus may also contribute to adaptation. The extensive number of internal introns found within hemocyanin genes of Apogastropoda may facilitate the regulation of differential expression (discussed in more detail in Schäfer et al. 2021b). Several regulatory mechanisms associated with introns have already been identified (e.g., Chorev and Carmel 2012; Rose 2008, 2018) and include, among others, temperature-dependent gene expression (Airoldi et al. 2015; Evantal et al. 2018; Gotic et al. 2016; James et al. 2018). Differential expression of hemocyanin genes could help to control the availability of different paralogs that harbor different properties (e.g., varying oxygen affinities at different temperatures). Thus, the regulatory functions of introns could represent evolutionary advantages that promote intron accumulation in hemocyanin genes of Apogastropoda (for more details see Schäfer et al. 2021b). To analyze potential advantages of introns in hemocyanin genes, further studies should characterize their nucleotide sequences, investigate them for specific regulatory mechanisms and determine if there are differences in introns between paralogs within one species.

Our results show that the accumulation rate of introns maintained in hemocyanins in Caenogastropoda is different within various lineages and highest within Cerithioidea (Fig. 5). In addition, the two paralogous hemocyanin genes from the cerithioid gastropod *M. tuberculata* exhibit the largest variations in exon–intron structures that have been found within one gastropod species (small arrows with numbers on top in Fig. 5, cf. also Supplement 3). Furthermore, the 550 kDa mega-hemocyanin subunit represents the only hemocyanin gene that lacks a linker intron between two different functional units. The loss of regions coding for FU-g and FU-h, as well as the gain of six FU-f-coding sequence sections, additionally led to strong variations in the protein structures which influence its physiological features (typical 400 kDa hemocyanin and 550 kDa mega-hemocyanin subunits that differ in their oxygen binding capacity, cf. introduction above and Lieb et al. 2010). These extensive differences at the gene and protein levels may represent combined adaptations of hemocyanins that enable Cerithioidea to live in a variety of habitats. The members of this superfamily of Caenogastropoda are distributed in freshwater, brackish water and marine ecosystems in mostly warm temperate regions (for an overview, see Strong et al. 2011). Their habitats include some extreme biotopes, such as rocky intertidal shores, mudflats and mangrove forests, which include environmental conditions such as strongly changing temperatures, humidity differences and little oxygen availability. The fact that the strongest differences in paralogous hemocyanins (including the gene and protein levels) have been identified in a group of gastropods with such extremely diverse habitats may represent a further hint that the evolution of hemocyanin plays a major role in the evolution of molluscs. Since both genes can be expressed in different ratios (Lieb et al. 2010), they can help to adapt to different living conditions.

## Conclusions

The oxygen affinity of molluscan hemocyanins is strongly influenced by abiotic factors such as temperature (Mangum 1990). A multitude of adaptations in these oxygen transport proteins have already been described (e.g., Strobel et al. 2012) and seem to be indispensable for many molluscan species to ensure a sufficient oxygen supply. Our findings reveal a strong diversity of hemocyanin genes of Caenogastropoda, including multiple independent gene duplications within different caenogastropod groups as well as a strong accumulation of FU-internal introns (21–57) within their genes. Since gene duplications and intron gains have also been discovered within hemocyanin genes of Tectipleura (Schäfer et al. 2018, 2021b), they most likely represent general phenomena of hemocyanin gene evolution within Apogastropoda. This contrasts with the hemocyanin evolution of Vetigastropoda and may support the hypothesis that diversity in this oxygen transporter may increase adaptation. Therefore, gene duplications may provide new genes to be adjusted by mutation and selection, and the accumulation of introns may contribute to regulatory opportunities. Although follow-up studies are required to analyze the biochemical and physiological properties of apogastropod hemocyanins (e.g., varying oxygen affinities and different temperature dependencies of hemocyanin paralogs and differential expression patterns of their paralogous genes), our results indicate that the evolution of hemocyanins in Heterobranchia and Caenogastropoda may be one of many influencing factors that enabled the large diversity of Apogastropoda by facilitating adaptive radiation.

## Supplementary Information

Below is the link to the electronic supplementary material.

Supplementary file1 (PDF 168 kb)

Supplementary file2 (PDF 512 kb)

Supplementary file3 (PDF 145 kb)

## Declarations

### Data Availability

The cDNA sequences and gene structures obtained during the current study are available at the NCBI under the following accession numbers: LisaH1: BK014376; LisaH2: BK014375; PcH I: BK014379; PcH IIb: BK014378; PcH III: BK014377; NlH1: MT939254; NlH2: MT939255; RtH1: BK014286; RtH2: BK014287; MtH_400_: KC405575; MtH_550_: KC405576.
