# Supplementary material for: The Evolution of Hemocyanin Genes in Caenogastropoda: Gene Duplications and Intron Accumulation in Highly Diverse Gastropods
Source: J Mol Evol. 2021 Nov 10;89(9-10):639–55. doi: 10.1007/s00239-021-10036-y (PMC8599328; doi:10.1007/s00239-021-10036-y)

Supplement 1: Bayesian inference. The phylogenetic analysis is based on an amino acid sequence alignment and was conducted using MrBayes 3.2.6 (Huelsenbeck and Ronquist 2001) which is implemented in Geneious 9.1.8 (Kearse et al. 2012). The tree includes hemocyanins of the following Caenogastropoda: *Pomacea canaliculata* (PcH I+IIb+III), *Melanoides tuberculata* ($MtH_{400+550}$), *Littorina saxatilis* (LisaH1+2), *Rapana venosa* (RtH1+2) and *Nucella lapillus* (NlH1+2). It further encompasses hemocyanins of Tectipleura (*Helix pomatia* HpHαD+αN+β; *Cornu aspersum* CaHαD+αN+β; *Lymnaea stagnalis* LsH1+2), Lepetellida (*Haliotis tuberculata* HtH1+2; *H. rubra* HrH1+2; *Megathura crenulata* KLH1+2) and Cephalopoda (*Nautilus pompilius* NpH and *Enteroctopus dofleini* OdH). It is based on two parallel runs of four Monte Carlo Markov Chains (MCMC) with one million generations, a subsampling frequency of 500 and a burn-in of 250,000. Nodes are labelled with posterior probabilities (PP) computed by MrBayes.

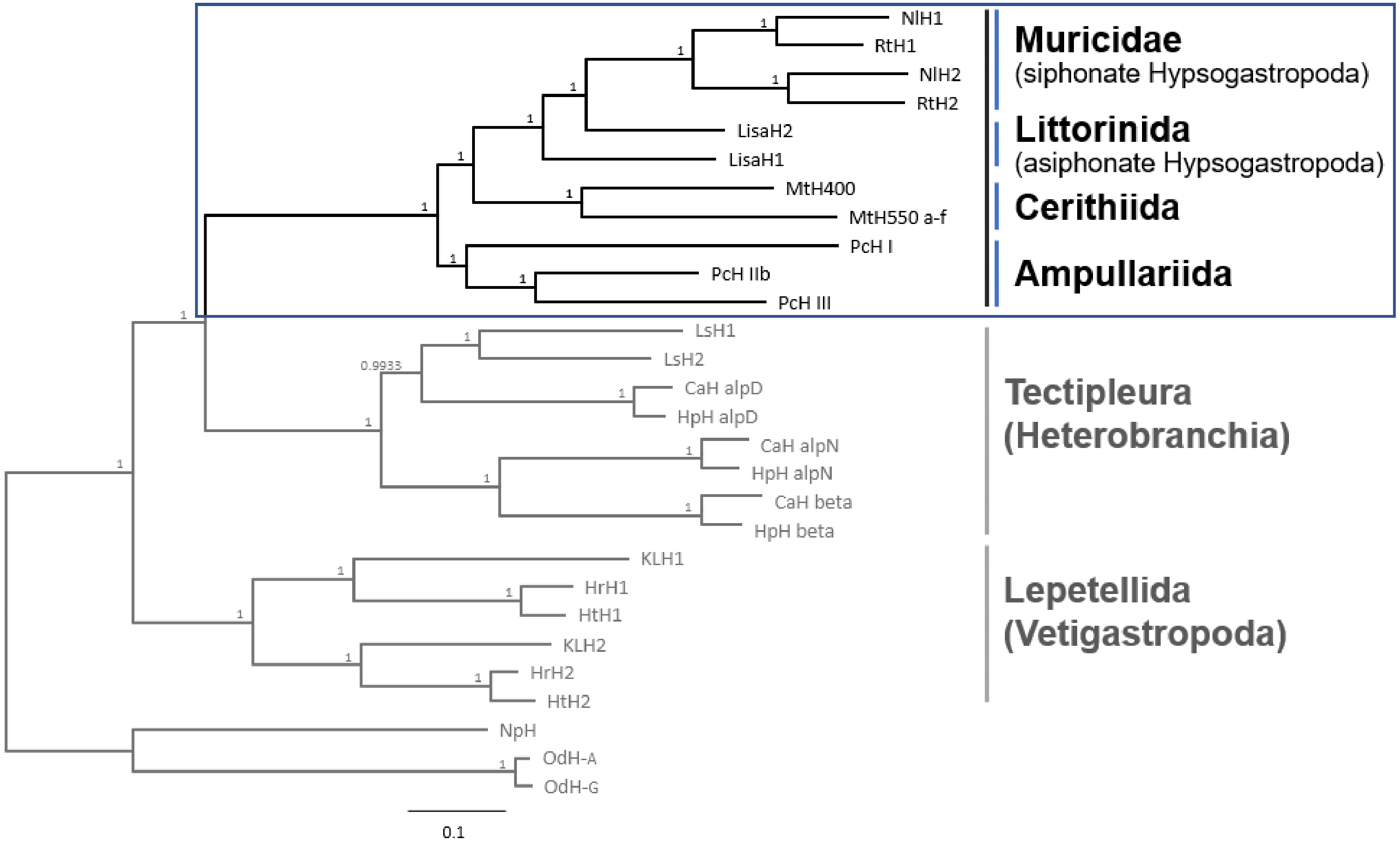

Supplement: Supplementary file 1 — Supplementary file1 (PDF 168 kb) [file 239_2021_10036_MOESM1_ESM.pdf]
