# Supplementary material for: The Evolution of Hemocyanin Genes in Caenogastropoda: Gene Duplications and Intron Accumulation in Highly Diverse Gastropods
Source: J Mol Evol. 2021 Nov 10;89(9-10):639–55. doi: 10.1007/s00239-021-10036-y (PMC8599328; doi:10.1007/s00239-021-10036-y)
Supplement: Supplementary file 2 — Supplementary file2 (PDF 512 kb) [file 239_2021_10036_MOESM2_ESM.pdf]

Supplement 2: Amino acid sequence alignment of hemocyanins. The alignment includes the full-length amino acid sequences of hemocyanins of the following molluscan species: *Nucella lapillus* (NlH), *Rapana venosa* (RtH), *Littorina saxatilis* (LisaH), *Melanoides tuberculata* (MtH), *Pomacea canaliculata* (PcH), *Lymnaea stagnalis* (LsH), *Megathura crenulata* (KLH, from keyhole limpet), *Haliotis tuberculata* (HtH), *Enteroctopus dofleini* (OdH) and *Nautilus pompilius* (NpH). Shading shows their similarity conservation with black = 100% and grey ≥ 80%. Splice site positions (with respect to the coding sequences of the amino acids) are highlighted in pink (internal introns) and yellow (linker introns). Positions 2,659 – 2,988 include the additional amino acid sequence sections within NlH2 and RtH2.

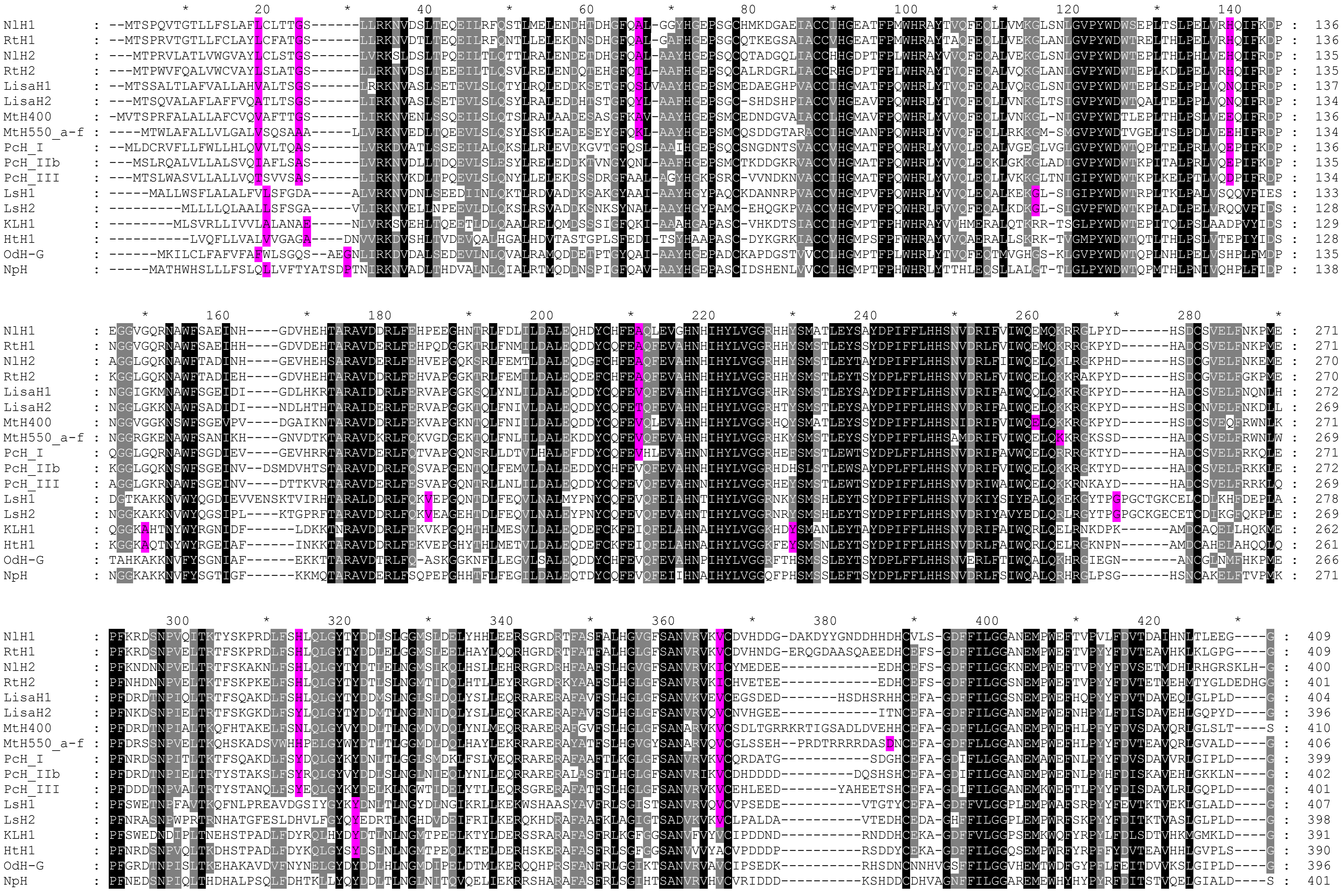

* 20 * 40 * 60 * 80 * 100 * 120 * 140
NlH1 : --MTSPQVTGTLLFSLAFLCLTTGS------LLRKNVDSLTEQEIIRFQSTLMELENDHTDHGFQAL-GGYHGEPSGCHMKDGAEIACCIHGEATFPMWHRAYTVQFEQLLVMKGLSNLGVPYWDWSEPLTSLPELVRHQIFKDP : 136
RtH1 : --MTSPRVTGTLLFCLAYLCFATGS------LLRKNVDTLTEQEIIRFQNTLLELEKDNSDHGFQAL-GAFHGEPSGCQTKEGSAIACCVHGEATFPMWHRAYTAQFEQLLVEKGLANLGVPYWDWTRELTHLPELVRHQIFKDP : 136
NlH2 : ---MTPRVLATLVWGVAYLCLSTGS------LVRKSLDSLTPQEIITLQTTLRALENDETDHGFQAL-AAYHGEPSGCQTADGQLIACCHHGDPTFPLWHRAYVVQFEQALVEKGLSNLGVPYWDWTEPLTHLPHLVEHQIFRDP : 135
RtH2 : ---MTPWVFQALVWCVAYLSLATGS------LLRKNVDSLTEEEITLQSVLRELENDQTEHGFQTL-AAFHGEPSGCALRDGRLIACCRHGDPTFPLWHRAYTVQFEQALVQKGLANLGVPYWDWTEPLKDLPELVRHQIFRDP : 135
LisaH1 : --MTSSALTLAFVALLAHVALTSGS------LRRKNVASLSETEVISLQTYLRQLEDDKSETGFQSLVAAYHGEPSMCEDAEGHPVACCVHGMATFPQWHRLYTVQFEQALVQKGLSNLGVPYWDWTEPLSELPPLVQNQIFRDP : 137
LisaH2 : ---MTSQVALAFLAFFVQATLTSGS------LIRKNVASLSETEVISLQSYLRALEDDHTSTGFQYL-AAFHGEPSGC-SHDSHPIACCVHGEAVFPQWHRLYTVQFEQLLVNKGLTSIGVPYWDWTQALTELPPLVQNQIFRDP : 134
MtH400 : -MVTSPRFALALLAFCVQVAFTTGS------LIRKNVENLSSQEIISLQTSLRALAADESASGFKAV-AAYHGEPSMCEDNDGVAIACCLHGMAVFPQWHRLYVVQFEQLLVNKGL-NIGVPYWDTLEPLTHLPSLVEEQIFRDP : 136
MtH550_a-f : ----MTWLAFALLVLGALVSQSAAA-----LLVRKNVEDLTQEEVISLQSYLSKLEADESEYGFQKL-AAYHGEPSMCQSDDGTARACCIHGMANFPQWHRLYVVQFEQLLRKKGM-SMGVPYWDTVGELTSLPDLVEEHIFRDP : 134
PcH_I : --MLDCRVFLLFWLLHLQVVLTQAS------LVRKDVATLSSEEIIALQKSLLRLEVDKGVTGFQSL-AAIHGEPSQCSNGDNTSVACCVHGMATFPQWHRLYVVQLEQALVGEGLVGLGVPYWDWTQPLTELPRLVQEPIFKDP : 136
PcH_IIb : ---MSLRQALVLLALSVQIAFLSAS------LVRKNVDLLTDQEVISLESYLRELEDDKTVNGYQNL-AAFHGEPSMCTKDDGKRVACCVHGMATFPQWHRLYVVQLEQKLGKKGLADIGVPYWDWTKPITALPRLVQEPIFKDP : 135
PcH_III : ---MTSLWASVLLALLVQTSVVSAS------LIRKNVKDLTPQEVISLQNYLLELEKDSSDRGFAAL-AGYHGKPSRC-VVNDKNVACCIHGMATFPQWHRLYVVQLEQLLVKKGLTNIGIPYWDWTKPLKELPTLVQDPIFRDP : 134
LsH1 : -----MALLWSFLALALFVLSFGDA-----ALVRKNVDNLSEEDIINLQKTLRDVADDKSAKGYAAI-AAYHGYPAQCKDANNRPVACCVHGMPVFPQWHRLYVVQLEQALKEKGL-SIGIPYWDWTRPLTKLPALVSQQVFIES : 133
LsH2 : ---------MLLLLQLAALLSFSGA-----LIRKNVELLNPEEVLDLQKSLRSVADDKSNKSYNAL-AAYHGEPAMC-EHQGKPVACCVHGMPVFPQWHRLFVVQFEQALKDKGL-SIGVPFWDWTKPLADLPELVRQQVFIDS : 128
KLH1 : --------MLSVRLLIVVLALANAE-----NLVRKSVEHLTQEETLDLQAALRELQMDSSSIGFQKI-AAAHGAPASC-VHKDTSIACCIHGMPTFPHWHRAYVVHMERALQTKRR-TSGLPYWDWTEPITQLPSLAADPVYIDS : 129
HtH1 : ----------LVQFLLVALVVGAGA----DNVVRKDVSHLTVDEVQALHGALHDVTASTGPLSFEDI-TSYHAAPASC-DYKGRKIACCVHGMPSFPFWHRAYVVQAERALLSKRK-TVGMPYWDWTQTLTHLPSLVTEPIYIDS : 128
OdH-G : ------MKILCLFAFVFAFWLSGQS--AEGNLIRKDVDALSEDEVINLQVALRAMQDDETPTGYQAI-AAYHGEPADCKAPDGSTVVCCLHGMPTFPLWHRLYTVQFEQTMVGHGS-KLGVPYWDWTQPLNHLPELVSHPLFMDP : 135
NpH : -----MATHWHSLLLFSLQLLVFTYATSDPTNIRKNVADLTHDVALNLQIALRTMQDDNSPIGFQAV-AAYHGEPASCIDSHENLVVCCLHGMPTFPHWHRLYTTHLEQSLLALGT-TLGLPYWDWTQPMTHLPNIVQHPLFIDP : 138
* 160 * 180 * 200 * 220 * 240 * 260 * 280 *
NlH1 : EGGVGQRNAWFSAEINH----GDVHEHTARAVDDRLFEHPEEGHNTRLFDLILDALEQHDYCHFEAQLEVGHNHIHYLVGGRHHYSMATLEYSAYDPIFFLHHSNVDRIFVIWQEMQKRRGLPYD------HSDCSVELFNKPME : 271
RtH1 : NGGVGQRNAWFSAEIHH----GDVDEHTARAVDDRLFEHPQDGGKTRLFNMILDALEQHDYCHFEAQFEVAHNHIHYLVGGRHHYSMSTLEYSSYDPIFFLHHSNVDRIFVIWQEMQKRRGKPYD------HADCSVELFNKPME : 271
NlH2 : AGGLGQKNAWFTADINH----GEVHEHSARAVDERLFEHVEPGQKSRLFEMTLDALEQDGFCHFEAQFEVAHNHIHYLVGGRHHYSMSTLEYTAYDPIFFLHHSNVDRLFIIWQELQKLRGKPHD------HSDCGVELFNKEME : 270
RtH2 : KGGLGQKNAWFTADIEH----GDVHEHTARAVDDRLFEHVAPGGKTRLFEMILDALEQDEFCHFEAQFEVAHNHIHYLVGGRHHYSMSTLEYTSYDPLFFLHHSNVDRLFVIWQELQKKRAKPYD------HSDCGVELFGKPME : 270
LisaH1 : NGGIGKMNAWFSGEIDI----GDLHKRTARAIDDRLFERVAPGGKSQLYNLILDALEQDDYCQFEVQFEVAHNHIHYLVGGRHRYSMSTLEYSAYDPIFFLHHSNVDRIFAIWQQLQKKRGKPYD------HADCSVELFNQNLH : 272
LisaH2 : NGGLGKKNAWFSADIDI----NDLHTHTARAIDERLFERVAPGGKTQLFNIVLDALEQDDYCQFETQFEVAHNHIHYLVGGRHTYSMSTLEYSAYDPIFFLHHSNVDRIFAIWQELQKKRGKPYD------HSDCNVELFNKDLL : 269
MtH400 : NGGVGGKNSWFSGEVPV----DGAIKNTARAVDDRLFEKVAPGKNTQLFNIILDALEQDDYCQFEVQLEVAHNHIHYLVGGRHQYSMATLEYSSYDPIFFLHHSNIDRIFVIWQELQKKRGKPYD------HSDCSVEQFRWNLK : 271
MtH550_a-f : NGGRGKENAWFSANIKH----GNVDTKTARAVDKRLFQKVGDGEKTQLFNLILDALEKDDYCQFEVQFEVAHNHIHYLVGGRHKYSMSTLEYSSYDPIFFLHHSAMDRIFVIWQELQKKRGKSSD------HADCSVELFRWNLW : 269
PcH_I : QGGLGQRNAWFSGDIEV----GEVHRRTARAVDERLFQTVAPGQNSRLLDTVLHALEFDDYCQFEVHLEVAHNNIHYLVGGRHEFSMSTLEWTSYDPLFFLHHSNVDRIFAVWQELQRRRGKTYD------HADCSVELFRKQLE : 271
PcH_IIb : KGGLGQKNSWFSGEINV--DSMDVHTSTARAVDERLFQSVAPGENTQLFEMVLDALEEDDYCHFEVQFEVAHNHIHYLVGGRHDHSLSTLEWSAYDPLFFLHHSNVDRIFVIWQELQKKRGKTYD------HADCSVELFRKKLE : 272
PcH_III : AGGLGKRNAWFSGEINV----DTTKVRTARAVDERLFESVAPGENTRLLNLILDALEQDDYCQFEVQFEVAHNHIHYLVGGRHEYSMSTLEWTSYDPIFFLHHSNVDRIWAIWQELQKKRNKAYD------HADCSVELFRRKLQ : 269
LsH1 : DGTKAKKNVWYQGDIEVVENSKTVIRHTARALDDRLFQKVEPGQNTDLFEQVLNALMYPNYCQFEVQFEIAHNTIHYLVGGRNKYSMSHLEYTSYDPIFFLHHSNVDKIYSIYEALQKEKGYTPGPGCTGKCELCDLKHFDEPLA : 278
LsH2 : NGGKAKKNVWYQGSIPL----KTGPRFTARAVDDRLFQKVEAGEHTDLFEQVLNALEYPNYCQFEVQFEVAHNTIHYLVGGRNRYSMSHLEYTSYDPIFFLHHSNVDRIYAVYEDLQRLRGYTPGPGCKGECETCDIKGFQKPLE : 269
KLH1 : QGGKAHTNYWYRGNIDF------LDKKTNRAVDDRLFEKVKPGQHTHLMESVLDALEQDEFCKFEIQFELAHNAIHYLVGGKHDYSMANLEYTAYDPIFFLHHSNVDRIFAIWQRLQELRNKDPK------AMDCAQELLHQKME : 262
HtH1 : KGGKAQTNYWYRGEIAF------INKKTARAVDDRLFEKVEPGHYTHLMETVLDALEQDEFCKFEIQFELAHNAIHYLVGGKFEYSMSNLEYTSYDPIFFLHHSNVDRLFAIWQRLQELRGKNPN------AMDCAHEIAHQQLQ : 261
OdH-G : TAHKAKKNVFYSGNIAF------EKKTTARAVDTRLFQ-ASKGGKNFLLEGVLSALEQDDYCHFEVQFEVAHNPIHYLVGGRFTHSMSSLEYTSYDPLFFLHHSNVERLFTIWQALQKHRGIEGN-------ANCGLNMFHKPME : 266
NpH : NGGKAKKNVFYSGTIGF------KKMQTARAVDERLFSQPEPGHHTFLFEGILDALEQTDYCQFEVQFEIIHNAIHYLVGGQFPHSMSSLEFTSYDPLFFLHHSNVDRLFSIWQALQRHRGLPSG------HSNCAKELFTVPMK : 271
300 * 320 * 340 * 360 * 380 * 400 * 420 *
NlH1 : PFKRDSNPVQITKTYSKPRDLFSHLQLGYTYDDLSLGGMSLDELYHHLEERSGRDRTFASFALHGVGFSANVRVKVCDVHDDG-DAKDYYGNDDHHDHCVLS-GDFFILGGANEMPWEFTVPVLFDVTDAIHNLTLEEG----G : 409
RtH1 : PFKRDSNPVELTRTFSKPRDLFSHLQLGYTYDDLELGGMSLEELHAYLQQRHGRDRTFATFALHGLGFSANVRVKVCDVHNDG-ERQGDAASQAEEDHCEFS-GDFFILGGANEMPWEFTVPYYFDVTEAVHKLKLGPG----G : 409
NlH2 : PFKNDNNPVELTRTFSKAKNLFSHLQLGYTYDTLELNGMSIKQLHSLLEHRRGRDRHFAAFSLHGVGFSANVRVKICYMEDEE------------EDHCEFS-GDFFILGGKNEMPWEFTVPYYFDVSETMDHLRHGRSKLH-G : 400
RtH2 : PFNHDNNPVELTKTFSKPKELFSHLQLGYTYDTLSLNGMTIDQLHTLLEYRRGRDRKYAAFSLHGLGFSANVRVKICHVETEE------------EDHCEFS-GDFFILGGSNEMPWEFTVPYYFDVTETMEHMTYGLDEDHGG : 401
LisaH1 : PFDRDTNPIQLTRTFSQAKDLFSHLQLGYTYDDMSLNGLSLDQLYSLLEQRRARERAFAVFSLHGLGFSANVKVEVCEGSDED-------HSDHSRHHCEFA-GDFFILGGSNEMPWEFHQPYYFDVTDAVEQLGLPLD----G : 404
LisaH2 : PFNKDSNPIELTRTFSKGKDLFSYLQLGYTYDDMTLNGLNIDQLYSLLEQRKARERAFAVFSLHGLGFSANVRVQVCNVHGEE------------ITNCEFA-GDFFILGGANEMPWEFNHPYLFDISDAVEHLGQPYD----G : 396
MtH400 : PFDRDTNPIALTKQFHTAKELFSNLQLGYTYDTLTLNGMDVDQLYNLMEQRRARERAFGVFSLHGLGFSANVRVKVCSDLTGRRKRTIGSADLDVEHHCEFA-GDFFILGGANEMPWEFHLPYYFDVSDAVQRLGLSLT----S : 410
MtH550_a-f : PFDRSSNPVELTKQHSKADSVWHHPELGYWYDTLTLGGMDLDQLHAYLEKRRARERAYATFSLHGVGYSANARVQVCGLSSEH--PRDTRRRRDASDNCEFA-GDFFLLGGANEMPWEFHLPYYFDVTEAVQRLGVALD----S : 406
PcH_I : PFNRDSNPITLTKTFSQAKDLFSYDQLGYKYDNLTLGGLSMDKLFSLVEQRRARERAFAAFTLKGLGFSANVRVKVCQRDATG-----------SDGHCEFA-GDIFLLGGANEMAWEFNLPYYFDVSDAVVRLGIPLD----G : 399
PcH_IIb : PFDRDTNPIELTRTYSTAKSLFSYRQLGYVYDDLSLNGLNIEQLYNLLEQRRARERALASFTLHGLGFSANVRIKVCDHDDDD---------DQSHSHCEFA-GDIFILGGANEMPWEFNLPYHFDISKAVEHLGKKLN----G : 402
PcH_III : PFDDDTNPVALTRTYSTANQLFSYEQLGYKYDELKLNGWTIDELYTLLEQRSGRERAFATFSLHGLGFSANVRVKVCEHLEED-------YAHEETSHCEFA-GDIFILGGANEMKWEFTLPYYFDISDAVLRLGQPLD----G : 401
LsH1 : PFSWETNPFAVTKQFNLPREAVDGSIYGYKYDNLTLNGYDLNGIKRLLKEKWSHAASYAVFRLSGISTSANVRVQVCVPSEDE----------VTGTYCEFA-GDFFVLGGPLEMPWAFSRPYYFEVTKTVEKLGLALD----G : 407
LsH2 : PFNRASNPWPRTRNHATGFESLDHVLFGYQYEDRTLNGHDVDEIFRILKERQKHDRAFAAFKLAGIGTSADVKVKVCLPALDA----------VTEDHCEDA-GHFFILGGPLEMPWRFSKPYYFDITKTVASLGLPLD----G : 398
KLH1 : PFSWEDNDIPLTNEHSTPADLFDYRQLHYDYDTLNLNGMTPEELKTYLDERSSRARAFASFRLKGFGGSANVFVYVCIPDDND----------RNDDHCEKA-GDFFVLGGPSEMKWQFYRPYLFDLSDTVHKMGMKLD----G : 391
HtH1 : PFNRDSNPVQLTKDHSTPADLFDYKQLGYSYDSLNLNGMTPEQLKTELDERHSKERAFASFRLSGFGGSANVVVYACVPDDDP----------RSDDYCEKA-GDFFILGGQSEMPWRFYRPFFYDVTEAVHHLGVPLS----G : 390
OdH-G : PFGRDTNPISLTKEHAKAVDVFNYNELGYDYDDLHLNGMDIPELDTMLKERQQHPRSFANFRLGGIKTSANVRVAVCIPSEDK----------RHSDNCNNHVGSFFILGGVHEMTWDFGYPFLFEITDVVKSLGIPLD----G : 396
NpH : PFNEDSNPIQLTHDHALPSQLFDHTKLLYQYDDLTLNGLNITQVQELIEKRRSHARAFASFRLSGIHTSANVRVHVCVRIDDD----------KSHDDCDHVAGNFFILGGAREMEWHYHYPYRFDITSTVQELGIALD----S : 401

```
                    440       *       460       *       480       *       500       *       520       *       540       *       560       *       580
NlH1       : HYGVHVEVYSVNGTRLGAHVLGTTPHLSHRPARGYHDPEIPDGRSGK-------TVVRKDVEVLTKEEMYHLRQAMAKFQNDTSIDGFQAVAEFHGLPAKCPHPDAAVRYACCVHGMPTFPHWHRLFVTEVEDELKSRGLEIGMP : 547
RtH1       : HYGVKADVYSVNGTLLGAHVLGSRPYLSHRPAEGYHDPEIHPDLKKT-------TLVRKDVETLTDEEVYYLRQAMAKFQNDTSIDGFQAVAEFHGLPPKCPHPDAAVRYACCIHGMATFPHWHRLFVTEVEDELKSRGLEFGIP : 547
NlH2       : HFEVKAEVYSVNGTRLESDIL-SKPYISHRPAHGFTDPVIHHSRRAT-------TVGRKNVDVLTDEEVYHLRQALARFQNDTSIDGFQAVAEFHGLPAKCPHPDAAVRYACCLHGMPTFPHWHRLFVTEVEDELRSRGLDIGIP : 537
RtH2       : HLEVKAEVYSVNGTLLGSQIL-GKPYISHRPAHGFTDPVIHRNRGTT-------TVGRKDVEVLTDEEVYHLRQALARFQNDTSIDGYQAVAEFHGLPAKCPHPDAAVRYACCIHGMPTFPHWHRLFVTEVEDELRSRGLEIGIP : 538
LisaH1     : RYHVSAEVFSVNGTSLGSGDL-PHPYGSHRPPHGHTDPTPGDGKGPT-------TVVRKDIAVLTDEEVYALRQAMARFQNDTSVDGFQAVAEFHGLPAKCPHPDAALRYACCIHGMATFPHWHRLFVVEVEDELRSRGLEFGIP : 541
LisaH2     : LYKVRADVYSVNGTMLGSNVL-PAPWGSHRPARGHTDPVIAEHHVAT-------NVVRKDIEVLTDEEVYALRVAMSRFQNDTSIDGFQAVAEFHGLPAKCPTPDAAVRYACCVHGMAVFPHWHRLFVVEVEDELRSRGLEIGIP : 533
MtH400     : KWSLEWDLYHVNGTALPGNLL-PTPYGKHRPAKGDRDSEEVSFFADRDVHRH--AVVRKNVVDLTDEEVYELRLAMDRFQNDTSVDGYQAIAEFHGLPARCPRPDAAVRYACCIHGMATFPHWHRLFVVQIEDELKARDLHFGMP : 552
MtH550_a-f : AWTVKVDFYHVNGTAMPGDIL-PPPVGAHRPAKGAHDSDDIHGHSGPD------IVVRKDVSELTAEEMYELRQAMSKFQNDTSVDGYQAVAEFHGLPARCPRPDAAVRYACCIHGMATFPHWHRLFVVQIEDELRARGLHIGVP : 544
PcH_I      : DFLVKAELYSVNGTLLSSGLL-PPPIGAHRPARGYRDPVPAMYRTDA-------TVVRKNVKALTDEETLALRQALSRFQNDTSVDGFQAVAEFHGLPARCPHPDARVRYACCLHGVPTFPHWHRLFVVQVEDALRTRGLDIGIP : 536
PcH_IIb    : DYVVTAELYSVNGTSLNPNLL-PRPVGVYRPAKGFTDPKPSESDVQSAN-----VVIRKDVDTLTDEEVYELRQALSRFQNDTSVNGYQSVAEFHGLPARCPRPDARVRYACCVHGMATFPHWHRLFVVQVEDALKSRGLHFGIP : 541
PcH_III    : NYIIQTEVFSVNGTELQAHVL-PDPVGAHRPVRGWTDPKPAGLEARTAK-----TVIRKDVDVLTDEEIYALRQALSRFQNDSSVDGYQAVAEFHGLPARCPYPDARNRYACCIHGMATFPHWHRLFVVQVEDALERRGLEIGIP : 540
LsH1       : NYRIEAELFSVNGTQLPSSLL-PHPFVSYRPAKGTVDTPIAHNEVNHEHEYHEGISVRKDIDRLTNEEIYELREALQKFQNDKSVDGYQAIAEFHGDPGKCPNPNSKNRKACCIHGMPTFPHWHRLLVVQVEDALRRRGSNIGVP : 551
LsH2       : NYHVEAEIYSVNGTLLPPGLL-PAPYVSYRPAKGKIDPVIEISGPDHDHEK---VAVRKNVDRLTDVEIYELRQALKKFQNDKSVDGYQAIAEFHGDPGKCPNPTAKDRMACCVHGMPTFPHWHRLLVVQVEDALRRRGSPIGIP : 539
KLH1       : HYTVKAELFSVNGTALPDDLL-PHPVVVHHPEKGFTDPPVKHHQSAN-------LLVRKNINDLTREEVLNLREAFHKFQEDRSVDGYQATAEYHGLPARCPRPDAKDRYACCVHGMPIFPHWHRLFVTQVEDALVGRGATIGIP : 528
HtH1       : HYYVKTELFSVNGTALSPDLL-PQPTVAYRPGKGHLDPPVHHRHDDD-------LIVRKNIDHLTREEEYELRMALERFQADTSVDGYQATVEYHGLPARCPRPDAKVRFACCMHGMASFPHWHRLFVTQVEDALVRRGSPIGVP : 527
OdH-G      : NYYVHADVTAINGTLLPDGTI-PRPTVSYIPAHNFKDADMVVVDKTG-------LNVRKDLQSLTTEEEYELRVAMERFMDDKSIDGYQALAEFHGLPAKCPEPDAINRVACCVHGMSTFPHWHRLVVMQFEDALLARGSPIGVP : 533
NpH        : NYYVDIDLIAINGTLLSSSLI-PNPTVDYQPAPGSSDPPMRQIDREK-------LVVRKDLRRLHDTEEFGLRQAMARFMKDESIDGFQALAEYHGLPARCLTPEG-NRIACCIHGMATFPHWHRLFVLQVEDALIRRGSPIGIP : 537

                         *       600       *       620       *       640       *       660       *       680       *       700       *       720
NlH1       : YWEWTRPNTHVPELAAEETYEDPHT-HHQVHNPFHDAVVAFLGE--KTSRDIQEDELSETPDFGDHTSLFDAMLLAFEQEDFCDFEVQFEVVHNAIHFLVGGLNPYTMATLHYSAYDPIFYLHHSNVDRLWAIWQRLQMRRGKLY : 689
RtH1       : YWDWTRPNTHVPALAAEETYEDPHT-HHQVHNPFHDALVAFLKK--KTTRDVQAD-LTETPAFGDHTALFDGMLLAFEQTDFCDFEVQFEVVHNAIHFLVGGFDPYTMATLHYSAYDPIFYLHHSNVDRLWAIWQKLQMRRGKLY : 688
NlH2       : YWDWTEPETHVPALANDETYEDPHT-HEQLHNPFHDAPIVFLGE--KTTRQIQDE-LSEKPKFGDHTALFDGMLLAMEQTDYCDFEVQFEVVHNAIHFLVGGFGPYTLTTLHYSAFDPIFYLHHSNVDRLWAIWQELQMRRGLPY : 678
RtH2       : YWDWTKPNAHVPALANDETYEHPYT-HETIHNPFHDAAVAFLGQ--RTTRQIQDD-LAETPKFGDHTTLFDGMLLAFEQTDFCDFEVQFEVVHNAIHFLVGGFGPYTMATLHYSAFDPIFYLHHSNVDRLWAIWQELQMKRGLPY : 679
LisaH1     : YWDWTKPNAAIPALAADITYTDPHS-GVEVHNPFHDAAVAFLGE--RTTRDTMVLELSKNAEFGDHTPLFDGILLAFEQTDFCDFEVQFEVTHNAPHFLVGGFAPYSLTTLHYSAFDPIFYLHHSNVDRLWAIWQKLQIIRGLPY : 683
LisaH2     : YWDWTRPNAHIPALAADETYEDPIT-HETVHNPFQDAAIAFLDQ--RTSRQVQDD-LSETPKFGDHTSLFDGILLAFEQDDYCDFEVQFEVTHNAPHFLIGGFAPYSMATLHYSAFDPIFYLHHSNVDRLWAIWQQLQMRRGKPY : 674
MtH400     : YWDWTVPNAQIPAIAAEATYQDPHHPDVTLHNPFYDAAVAFLNT--RTTRDVQND-LAENPIYGDHSKLYDGILLAFEQTDFCDFEIQFEVIHNSPHFLVGGFGAYTLATLHYSAFDPIFYLHHSNVDRMWAIWQELQIRRGLPY : 694
MtH550_a-f : YWDWTRPNTHIPKLAAEATYQDPHHEGVTLNNPFYEAAVAFLGK--KTERDVQSQ-LSENPPFGDHTDLFDGMLLAFEQTDFCDFEIQFEVTHNAPHFLVGGFKPYSLATLHYSAFDPIFYLHHSNVDRLWAIWQELQLRRGLPY : 686
PcH_I      : YWDWTGMDTTVPPLADDDVYLDPIT-GEFQHNPFHDAPVAFIGE--RTSRQVQAE-LTQTPKFGDHTDLFDGMLLAFEQTDFCDFEIQFEVVHNAPHFLVGGFAPYSLATLHYSAFDPIFYLHHSSVDRMWAIWQALQVLRGLPY : 677
PcH_IIb    : YWDWTRPGTTVPAIASKETYVDPNS-GSSVHNPFFNAPVAFLGE--TTSRQVQPE-LSTKPEYGDHTALFDGMLLALEQRDFCDFEVQFEVVHNLPHFLVGGFASYSLSTLHYSAFDPIFILHHSNVDRLWAIWQALQIKRGLPY : 682
PcH_III    : YWDWTKPGTKIPAFVKEETYEDPVS-KNMVHNPFFNAPVAFLKE--TTQREIQYD-LFKNPKFGDHTDLFDGMLLAFEQTDFCDFEVQFEVIHNLPHFMVGGFAQFSLSTLHYSAFDPIFILHHSNVDRLWAIWQALQIKRGLPY : 681
LsH1       : YWDWTKPRTSVPKLAADETYTDPTT-NEVKRNPFHDAPIAFLGDSTKTTRDVSSS-LSDSPKWGDHTELFDAFLLALEQDNFCDFEVQFEVAHNLIHALVGGKEQYSLATLSYSAFDPIFYLHHSNVDRIWAIWTALQELRGKPY : 694
LsH2       : YWDWTRPNTKIPDLANDVKYIDPKT-KDELDNPFHDAEISFLDNNVHTSRDVQKD-LSQTPLWGDHTELFDAFLLALEQDNFCDFEVQFEIAHNLIHGLVGGNSKYGLSSLSYSAFDPIFYIHHSNVDRIWAIWTALQQHRGKPY : 682
KLH1       : YWDWTEPMTHIPGLAGNKTYVDSH--GASHTNPFHSSVIAFEENAPHTKRQIDQR-LFKPATFGHHTDLFNQILYAFEQEDYCDFEVQFEITHNTIHAWTGGSEHFSMSSLHYTAFDPLFYFHHSNVDRLWAVWQALQMRRHKPY : 670
HtH1       : YWDWTKPMTHLPDLASNETYVDPY--GHTHHNPFFNANISFEEGHHHTSRMIDSK-LFAPVAFGEHSHLFDGILYAFEQEDFCDFEIQFELVHNSIHAWIGGSEDYSMATLHYTAFDPIFYLHHSNVDRLWAIWQALQIRRHKPY : 669
OdH-G      : YWDWTTPSSSLPHLVAVETYEDPYT-KEVKPNPFYHAKIEFLHNDVFTARNVDSR-LFEKPTKGHHGYLHDGMLLAFEQEDFCDFEVQFEVTHNAIHAWVGGNEPYSMSSLHYTSFDPLFWLHHSQVDRLWAVWQALQIHRGKPY : 676
NpH        : YWDWARPMISLPHLIADETYINPYS-NKSVPNPFHHGSIDFLHDHITTQRDIKQETLFEQPAFGDHTYLFDGLMYALEQDDFCDFEIQFEIVHNGIHAWTGGQQTYSMSSLHFTSFDPLFFLHHSNVDRIWAIWQALQIYRGKHY : 681

                  *       740       *       760       *       780       *       800       *       820       *       840       *       860       *
NlH1       : KAHCAGSLTEEVMKPFGFPPPYHNDPKTHDNARPDKVYDYEHVLEYTYDSLLFGGMTIEQLDHYLEER-QTHDRVFVGVMLHNIGISAWATLSIEMKNGE-NYTVGKLGVLGGEKEMPWHFDRPYKVEITDALHKLGYRYDDDFH : 832
RtH1       : KAHCAGSLTQELMMPFGFPPPYHNDPKTHDNARPSQIYDYESVLDYTYDSLQFGGMTIAQLDHYLEER-KTHDRTFVGIMLHNIGISAWATLAIEMKNGE-EYTVGKVAVLGGEKEMPWHFDRTFKVEITEALHKLGYRYDDEYD : 831
NlH2       : KAHCAGSLTYEVMKPFGFLPPYHNDAKIHDNARPYKIYDYEKTLDYAYDSLQFGGMTIEQLDHYLEER-KSHDRAFVGIMLHNIGISAWATLSIEMTNGE-KYEVAKLAVLGGEKEMPWRFDRAYRVEITDALRHLGFSYDDEFH : 821
RtH2       : KAHCAESLSKEVMKPFGFPPPYHNDQKIHDNARPFKIYDYESVLDYTYDSLQFGGMTVDQLDPYLEER-KTHDRVFVGIMLHNIGVSAWCTLSIMRASGD-KYEVTKLAVLGGEKEMPWRFDRAFKVDITEALKTLGFSYDDDFH : 822
LisaH1     : KAHCASTQTQEVLKPFGFPPPWNNDEKTFNNARPTNVYDYENVLDYTYDTLQFGGMTVDQLEHYLEER-QTRDRAFVGIALHNIGVSAWVDVKINA-PGGESYQVGHVAVLGGEKEMAWHFDRLFKLEITDALSALGLRYDDDFE : 826
LisaH2     : KAHCASTQTQELLKPFGFPPPWNNDEKTFSNARPTNIYDYEEVLDYTYDSLQFGGMTVDQLDHYIEER-QTRERTFAGILLHNIGVSAWVEVSIDV-KGGDPHVVGHVAVLGGEKEMAWRFDRLFKLEITDALHQMGFNYDDDFH : 817
MtH400     : RAHCAHTMTHEPMKPFSFAAPLNNNEKTFSHSEPSQVYDYERDLGYTYDSLTFGGMTIEQLENYLEER-QSKERVFVGIELHNIGASAWVKVLIDSGSGGEPYLVGKIAVLGGEKEMPWHFDRLFKMEITDALSDLGLSYDDNFD : 838
MtH550_a-f : EAHCANSMTHDLLKPFGFEAPLNNNEKTHSHARPTKIYDYELELDYTYDNLQFGGMTIEQLEHYLEER-QARERVFVGLEFHNIGVSAWADVYVVA--GGQRYRAGGVAVLGGEQEMPWHFDRLFKMEITDALKALGLGYNDDFT : 828
PcH_I      : QAHCALSLTRDTLKPFGFPSPLNNNQKTFSRARGDQVYEYQKNFLYDYDSLTFGGMTIPQLNQFLAER-GNRDRTFVGVKLHNIGVSAWVKLYISV-PGGERYLANSFAVLGGEREMTWTFDRLFKVEITDTLLAIGRNYDEDFD : 820
PcH_IIb    : KAHCAHTMTHELLKPFGFAPPLNSNEKTYSHARPTNIYDYEHELEYAYDNLQFGGMTIDQLDHYIDER-RTKDRTFVGLHLQNIGTSALVILSIKP-KDGDSYEVAKLAVLGGEKEMPWNFDRLVKVEITDALNKKGLKYDDEYD : 825
PcH_III    : KAHCANTLTHESLKPFGFPPPLNNNEKTFAHATPLNIYDYEKELGYGYDTLQFGGMTVDQLKTYIDDR-RAKDRTFVGILLHSIGVSAKVELSIQK-SGVAPYKVGALVVLGGEAEMPWNFDRVVKVEITKALTDLGLSYDDNFD : 824
LsH1       : KAHCAQSYTYEPLKPFAFSSPYNNDQKTFTHAVPRNVYDYETELNYAYDSLEFGGLTIKQLNDYINNERKTKNRVFAGVLLWGIKRSALATLYV------NDVEAGRFALLGGPSEMEWRFDRLYRHEITDTLEELNLHWAQPYT : 833
LsH2       : KAHCAQSYTYEPLKPFAFSSPYNNNEKTFSHSTPRNVYDYEAELGYAYDSLEFGGMAIPELDDFINSNLKGRSRVFVGILLSGIKKSGIAKIKVIT-MRNEEYEAGRFALLGGPSEMPWSLDRVYRHDITTALEKMGLFWAQPYE : 826
KLH1       : RAHCAISLEHMHLKPFAFSSPLNNNEKTHANAMPNKIYDYENVLHYTYEDLTFGGISLENIEKMIHEN-QQEDRIYAGFLLAGIRTSANVDIFIKT-TDSVQHKAGTFAVLGGSKEMKWGFDRVFKFDITHVLKDLDLTADGDFE : 813
HtH1       : QAHCAQSVEQLPMKPFAFPSPLNNNEKTHSHSVPTDIYDYEEVLHYSYDDLTFGGMNLEEIEEAIHLR-QQHERVFAGFLLAGIGTSALVDIFINK-PGNQPLKAGDIAILGGAKEMPWAFDRLYKVEITDSLKTLSLDVDGDYE : 812
OdH-G      : KPYCALSEVHRPLKPFAFEPPLNNNKHTHSHSVPTHVYDYQSDLHYTYDTLFFGGMSVRELQRHIEED-KAKDRVFVGFLLMGIKTSANVVINVES--AGNTYMAGTITILGGSKEMEWRFDRLYKYEITDALAELGVDMHAEYS : 818
NpH        : LAHCANSEVHSPMRPFSFPSPRNPNPRTFTHATPTDIYDYEAELGYTFDTLDFGGRSIPDLQNEIEEH-QRHDRTFAGFLFWGIHTSTNGKVFVDV-IGQATYNAGTIAILGGTKEMTWRFDRLYHFEISEALKALGKTADDEFS : 824
```

```
                    880         *        900         *        920         *        940         *        960         *        980         *       1000         *
NlH1       : VHMHLTDVKGHEWPEDTFSHHTIFHQPASIHHEEE--YHKDEHVRMDVETLTVEQEQYLREALATLKHDHSISGYNQIASFHGQPNWCPSLEAEHKFACCTHGMPIFPHWHRLLTVQAENALRAHGLHSGLPYWDWTLPMTELPK :  975
RtH1       : VKLHIKDVTGKEWPEDTFSHHTIIHVPASTHLEED--HKEEDHVRNDVDTLTKEQIQNMREALATLKQDHSPGGFDHLAAFHGQPNWCPSMQAEHKVACCPHGMPVFPHWHRLLTVQAENALIAHGMHSGLPYWDWTLPMTALPK :  974
NlH2       : VHLDIIDVTGKKWPEDTFSHETIIHVPAEVHTEDN--FEKDDHVRNDVEHLTVEQMQNLREALHSLKEDHTISGYNQIAAFHGQPNWCPSPQAEKKFSCCAHGMAIFPHWHRLMTVQMENALMANGMITGLPYWDWTLPMTELPF :  964
RtH2       : VHLDITDVTGKKWPKDTFSHDTIIHVPASAKAEDD--FASDDHVRSDVEHLTAEQMQNLREALRALMNDASIAGYNQIASFHGQPNWCPSPDAEEKFACCAHGMPIFPHWHRLLTVQAENALMAHGMTSGLPYWDWTLPIDHLPE :  965
LisaH1     : VHLHIVDVTGKQWPEDTFSHSTILHSPAQVHEHEGMTFGDREYLRSDVTSLSREQIQNLRDAMQSLESDTSINGYNQIAAFHGQPNWCPSPDAEKKYACCAHGMAIFPHWHRLLTVQAENALHANGLQDGLPYWDWTLPMETLPE :  971
LisaH2     : VHLDITDVNGKKWPEDTFSHTNIIHVPPKVHEEDG--YEKEERVRSDVDSLTVEQIQNIREALQAAKNDETVSGYNQIAAFHGQPNWCPSPAAEKKYACCTHGMAVFPHWHRLLTVQAENALVANGLHGGLPYWDWTLPITSLPA :  960
MtH400     : VTLQITDVTGKEWSSDSFQHDTIFHEPGKTFEVRG---VSDKRTRLDVDHLNSEQIQNLRDAFKALEDDTSINGFNQIAAFHGQPSWCPSPDAETKYACCLHGMPTFPHWHRLITVQLENALRDKGFESGLPYWDWTLPMTELPE :  980
MtH550_a-f : ISLNITDVNGKHVDENTFSHDTIFHDPPHVHHDAG-DAPSPDKVRSNIESLTREQVQNLRDALQALEDDSSNAGFAQIAAFHGEPRWCPSTDAEKKYACCLHGMAVFPHWHRLITVQAENALRAHGFEDGLPYWDWTRPMKALPQ :  972
PcH_I      : VTLDITDVQQQKLSVDTFSHTTIFHQPKKDYDGDA--PEEVGRMRYNVDSLTREQAQSLRDALQKLQNDSTVNGFGHIAAFHGQPSWCPAPDAPVKYACCVHGMAVFPHWHRLLTVQMENALRNKGFKEGLPYWDWTQPMSSLPD :  963
PcH_IIb    : VVLSIKDVSGKDWPENTFSHTTIIHEEGTTHEETG--EHQELHVRSDVSSLTREQVQSLREALQSLEDDTSINGFEHIAAFHGQPNWCPSPDAEKKFACCAHGMAVFPHWHRLLTVQAENALRAKGFQDGLPYWDWTLPMTALPE :  968
PcH_III    : VILSITDVNGLTLPEDTFSHHTIIHDDGDVIEPTT--KAVEKYVRSDLDSLSREQIQNLRDALQALKDDSSVRGYAQIAGFHGQPNWCPSPDVEKKVACCPHGMAIFPHWHRLITVQAENALRDRGFKDGLPYWDWTLPMTKLPD :  967
LsH1       : VTLKLTDFEGHDIPINPFPKPQIIFKAKEEPKDVS---ADTIKIRKNVATLTEAEVVDLRQALANLQNDQSAGGYQDLGRFHGTPNWCPTPDAEVKKSCCLHGMPTFPHWHRLWTVQAENALRRHGYVGAIPYWDWTQPVKSLPD :  975
LsH2       : VKLEMTEFDGTPIDVRLFPRIQLIYRAPEHPKAAA---EHDVHVRKNVDQLTEEEVHDLRQALSNLQDDKSNGGYQDLGRFHGTPKWCPSPEAEKKVSCCLHGMPTFPHWHRLHAVQAENALRRHGYHGGLPYWDWSRPLKSLPA :  968
KLH1       : VTVDITEVDGTKLASSLIPHASVIREHARVKFDKV---PRSRLIRKNVDRLSPEEMNELRKALALLKEDKSAGGFQQLGAFHGEPKWCPSPEASKKFACCVHGMSVFPHWHRLLTVQSENALRRHGYDGALPYWDWTSPLNHLPE :  955
HtH1       : VTFKIHDMHGNALDTDLIPHAAVVSEPAHPTFEDE---KHSLRIRKNVDSLTPEETNELRKALELLENDHTAGGFNQLGAFHGEPKWCPNPEAEHKVACCVHGMAVFPHWHRLLALQAENALRKHGYSGALPYWDWTRPLSQLPD :  954
OdH-G      : INLQINDINGTALPPTSIPDPIVIFSPGKKESGVV--FDELYRSRRDVSSLTDADMNALRKALQAYEDDKDASGYQQVAAFHGSTKWCPSPDAEVKYACCHHGMATFPHWHRLLTVNFENGLRHNGYQNGIPYWDWTRPLSELPT :  961
NpH        : LHVKIHDVNGSRLSNDIIPAPNIIFVPAHVKSMNI---SHKGHTRRNINSLTQESLYELRQALTSFMADTENTGFQRYAAFHGFPEWCPSPDAEHKFACCVHGMPTFVHWHRLLTIQFENGIRNHGYQCGLPYWDWTEAITHLPE :  966

              1020         *       1040         *       1060         *       1080         *       1100         *       1120         *       1140         *       1160
NlH1       : FVAVATYESPRTHHEEINPWYNAEVN--DHNTTRSVR-DELFQQPELGKLTRIAEKVMLAFEQDNFCDFEIQYEIAHNHIHALVGGNQLYSMASLRYTAFDPIFYLHHSNTDRIWAIWQVLQKIRGKPYNSANCALSELRKPLQP : 1117
RtH1       : FVADATYENPKTHHTEPNPWFSGEVD--GHNTSRMVR-DDLFEQPEFDKMTRIAEKVMLAFEQDNFCDFEIQYEIAHNHIHALVGGNKLYSMASLRYTAFDPLFFLHHSNTDRIWAIWQTLQKMRGKPYNSANCAIAELRKPLQP : 1116
NlH2       : LVREATYHNPKTDLDEPNPWYSGVVD--GHETHRNVR-EDLFKQPDFGHMTPLAEKVMLAFEQDNFCDFEIQYEIAHNYIHAVVGGTEEYSMASLRYTAYDPLFFLHHSSTDRIWAIWQHLQKFRNKPYNSANCAIAELRRPLQP : 1106
RtH2       : LVAGENFDNPKNGQSEPNPWFSGEVN--GHETQRNVR-DDLFKQPAFGEMTPLAEKVMLAFEQDNFCDFEIQFEIAHNFIHAVVGGTELYSMASLRYTAFDPLFYLHHSSTDRLWAVWQKLQKFRNKPYNSANCALAEMREPLQP : 1107
LisaH1     : IVRDETYIHPKTGQSTENPLHHAVVD--GHKTVRTLREEELFEAPAFGKLTRIAEKVMLAFEQTDFCDFEIQFEIAHNYIHALVGGNEEYSMASLRYTAYDPIFYLHHSNTDRIWAIWQQLQEYRGLPYNSANCAIASLRKPLQP : 1114
LisaH2     : IVKEEFYTNPKTGESTPNPLYNGLAD--GHPTTRSVR-DELFEAPAFGSLTRIAEQVMLAFEQTDFCDFEVQYEIAHNYIHALVGGNEEYSMASLRFSAYDPIFFLHHSNTDRLWAIWQELQKYRGLPYNSANCAIASLRQPLQP : 1102
MtH400     : SFTAETYDNPVSGANEANPLHHAVVE--GHKTTRSVR-GELFQQPAFGKFTRIGEQVMLAFEQTDYCSFEVQFEVAHNYIHALVGGNELYSMASLRYTAYDPLFALHHSNTDRIWAIWQALQKYRGLPYNTANCAITQLRKPLQP : 1122
MtH550_a-f : VVESAQYVNQQTGQTMDNPFHHGIAN--GHATERDVS-PKLFQQPEFGKFTDIAELVMFAFEQTDFCDFEVQMEIAHNYIHALVGGSKPYSMSSLRYTTYDPIFFLHHSNTDRIWAIWQALQKYRGLPYNTANCALASMRKPLQP : 1114
PcH_I      : FVEEESYQNPRTEDIEPNPFHHGTVE--GQDTTRSPR-EDLFKVPEYGQMTPVAEQVLLALEQTDFCSFEVQFEIAHNYIHALVGDKEEYSMASLRYTAYDPLFYLHHSNTDRLWAMWQVLQGYRGLPMFTANCALTSFRQPLQP : 1105
PcH_IIb    : IVAGETYVNPETNLPEPNPFHHGLVE--GKKTTRSVR-DELFEKPKFGRLTKIAEMVLLAFEQTDFCTFEVQYEIAHNYIHALVGGNESYSMASLRYTAYDPIFFLHHSNTDRIWAIWQALQHYRGLPYNSANCAIASLRKPLQP : 1110
PcH_III    : LVESDTYLNHKKNETVPNPFHHGEIG--NEKTTRSVR-DELFEQPEFGKLTKIAEMVLLAYEQTDFCDFEVQFEIAHNYIHALVGGKELLSMASLRYTAFDPLFFLHHSNTDRIWATWQALQRYRGLPYNSANCAIASMRTPLQP : 1109
LsH1       : LVTSEKYRDPSSNNEIKNPFYSAHITDADQDTVRSPR-DDLFEQPKFGQNTAIAKQVLLALEQDNFCDFEVQFEIAHNFIHALVGGNEQFSMASLLYTAFDPLFYLHHANTDRLWAIWQALQKYRNKPYNTANCAIGQLRKPLSP : 1119
LsH2       : LVSSPTYLDPSNNEQVKNPFYSAHIDDVDHDTVRSVR-SDLFQQPGYGEYTDIAEQILLALEQDNFCDFEVQFEIAHNFIHALVGDKEPYSMASLMYTAFDPLFYLHHSNTDRIWAIWQALQNYRGKPYNTANCAIGQLRQPLAP : 1112
KLH1       : LADHEKYVDPEDGVEKHNPWFDGHIDTVDKTTTRSVQ-NKLFEQPEFGHYTSIAKQVLLALEQDNFCDFEIQYEIAHNYIHALVGGAQPYGMASLRYTAFDPLFYLHHSNTDRIWAIWQALQKYRGKPYNVANCAVTSMREPLQP : 1099
HtH1       : LVSHEQYTDPSDHHVKHNPWFNGHIDTVNQDTTRSVR-EDLYQQPEFGHFTDIAQQVLLALEQDDFCSFEVQYEISHNFIHALVGGTDAYGMASLRYTAYDPIFFLHHSNTDRIWAIWQSLQKYRGKPYNTANCAIESMRRPLQP : 1098
OdH-G      : LVKDETYAD-ENGETHPNPFFSGVIDEIGEHTTRSPN-PTLFLKPPFGHFTPLGDEVMYALEQEDFCSFEVQFEIAHNHIHALVGGTEPYSMSSLEYTTFDPIFILHHSNVDRIWAIWQALQKFRGHRYNSANCAIETLRKPMSP : 1104
NpH        : SLTAAKYTDPHSNEMHHNPLHNGHIDSVNADTMRNIN-EKLFEQPEFGHFTLLANQVILALEQENFCDFSVQFEISHNEIHALVGGTEPYSMASLTYTAFDPLFMLHHSNVDRIWAIWQSLQQYRGHPHKSANCALQQLHQPLSP : 1110

                         *       1180         *       1200         *       1220         *       1240         *       1260         *       1280         *       1300
NlH1       : FAQTSVTNPDPVTRDNSAPFKVFNYRKSFHYRYDNLQFNGMSPPQLQREIIKRKGLERVFAGFMLHGIQKSSLVVFEICKPD-DTC-KQAGEFYLLGDEFEMPWEYDRLFKYEITDQLDKFDLKPTDRYDIHYTVYDLNRQNLGE : 1260
RtH1       : FAQTSVTNPDPVTRDHSVPFDVFKYHENFHYRFDNMQFNGMSPPQLLRETVRRKGLDRVFAGFMLHGIQKSALVVFEICKPD-GTC-KEAGEFYLLGDEFEMPWEYDRLFKYDITEQLEKFDLEPMDRYDIQYTVSDLNGQNLGD : 1259
NlH2       : FAQTSLTNPDPVTRDHSVPFDVFSYHDSFHYEYDNLQFNGMSIPQLHRETVKRKGLSRVFAGFMLHGIKKSSLVVFQICTPD-HTC-TDGGEFYLLGDEYEMPWEYDRLYKHEITSQLEKRGLDHHDRFDVEYHVYDLEENDLGS : 1249
RtH2       : FAQTSATNPDPVTRDHSVPFDVFNYRNSFHYEYDNFQFNGMSIPQLQRETLKRQGLDRVFAGFMLHGIKKSSLVVFNICTSD-KSC-TKAGEFYLLGDEFELPWEYDRLYKHEITKELKEMSLDHHDPFDIHYNVYDLENNDLGD : 1250
LisaH1     : FAQSSVVNPDPVTRDHAVPFDVFNYEDSFHYHYDNFQFNGLSIPQIQREVVRRQAKERIFAGFMLHGVEQSVLVVFEICKPN-GDC-KEAGEFYLLGDEYELPWSYDRLFKYEITDQLHEFDLQHGDNYDIHYVVYDLAGSSLGD : 1257
LisaH2     : FAQDHVTNPDPVTRDHSTPFDVFNYENSFHYHYDNLQFNGMSIPQIQREVIKRAAEERVFAGFMLHGIKKSSLVVFEICKPT-GDC-KKAGEFYLLGDEYELPWEFDRLFKYEITDQLSEFGLEPLDNYEVKYEVFDLAGASLGT : 1245
MtH400     : FAQTSTVNPDSVTRDHSVPFDVFDYSSSFHYDYDNLEFNGLSIAQLQHEVVVRKAHERVFAGFMLHGIKQSALVRFEICSSG-GAC-GQAGEFYILGDDYEMPWEYDRLFKYEITDQLKEQGLEPLDRYDIKYTVFALDGSVVGT : 1265
MtH550_a-f : FAQTSVVNPDPMTRDHSVPFEIFDYQSSFHYTYDDLEFNGLSIPQLQHEVVVRQAHERVFAGFMLKGIGQSALVTFEICQGS-GQC-SAAGEFYMLGDEYEMPWVYDRLFKYEITQQLADKGLKPLDSYYVQYQVKDLQNNVIGT : 1257
PcH_I      : FAQPSVVNPDPITRENSIPFRVFDYTRNFHYRYDNIDFNGLSIPQLQREIVKRQSRERVFVGFMLHGIQRSALVVLNLCQV---TC-QKAGEFYILGDANEMPWKYDRLFKYEITRLLAELKLRADDPYRVSYTVYDLNGQELED : 1246
PcH_IIb    : FAQTSTINPDHITRDHSVPFEVFDYERSFQYHYDDLEFNGFSIPQLQREILHRQHDERVFAGFMLHGIRKSALVTLEVCEPS-GPC-HPEGEFYILGDEYEIPWQYDRLFQYEITDILAKYKLEANDRYSVKYSVIDLDGKKISS : 1253
PcH_III    : FAQSSVINPDPVTRDHSVPFDTFDYQRSFHYSYDNFQFNGLSLPQIQREIVRRQTDDRIFVGFMLYGIKKSALVSFQIIDSN-NES-HKGGEFYILGDENEIPWQYDRLFKFEITRELAKYNIDQRDPFRIEYDVYNLAGEKISN : 1252
LsH1       : FSLTSSVNPDANTREHSLPFQVFDYRNNFHYEYDNLEFNGLSIPQLAKELEKNKGDDRVFVGFMLHGIKRSALIKFQIHKND--TEFKEAGEFYILGDENEMEWSYDRLYKYEVTQQLEDFGLRYNDRYYIKYQVLDLDGSIYST : 1262
LsH2       : FSLTSDVNPDAVTREHSVPFQVFDYRNNFHYTYDNLEFNGLTIAQLARKLEQNKDSDRVFAGFMLHGIGKSALVKFYICRSA-DDCNNYAGEFYILGDPHEMEWAYDRLYKYEITRQLSDLNLHYNDRYYIRYEVLELSGEVFST : 1256
KLH1       : FGLSANINTDHVTKEHSVPFNVFDYKTNFNYEYDTLEFNGLSISQLNKKLEAIKSQDRFFAGFLLSGFKKSSLVKFNICTDS-SNC-HPAGEFYLLGDENEMPWAYDRVFKYDITEKLHDLKLHAEDHFYIDYEVFDLKPASLGK : 1242
HtH1       : FGLSSAINPDRITREHAIPFDVFNYRDNLHYVYDTLEFNGLSISQLDRELEKIKSHERVFAGFLLSGIKKSALVKFEVCTPP-DNC-HKAGEFYLLGDENEMAWAYDRLFKYDITQVLEANHLHFYDHLFIRYEVFDLKGVSLGT : 1241
OdH-G      : FSLTSDINIDPMTREHSVPFDVFDYKKNFHYEYDLLELNGLSIPQLHREISRRRAKSRIFATFMLEGIKQSALVEYYIRAHG-STDQLKAGEFYILGSANEMPWKFDRVYKADITQQMKEANLHFNDQYHIEYHLKDLSGNEIA- : 1247
NpH        : FSLTSEVNPDPETREHSKPMDVFDYEHNFHYHYDNLELLGMSIPQLSREINRRRSRNRVFAGFMLHGFKKSALVSFSILHHQ--TDETYAGEFYILGSEDEMPWHYDRLYRYEITDILNELHLQYTDPLRITYRINDLHNKVVEG : 1253
```

```
                     *      1320          *      1340          *      1360          *      1380          *      1400          *      1420          *      1440          *
NlH1       : DLFGNATIVYKHGLGHMRGHEGDYLEEVMASSHVRKDMSTLINGECESLRSALHDMEEDGSFEQIAKFHGYPGLCDHK-GR---AV-GCCMHGSPIFPHWHRLYVEQVENALLNHGSAVSVPYWDWTEHFDELPNLIGKPTYFNS : 1400
RtH1       : DLFGNATIVYTPGLGHMKGHEEDYRAEVRASSHVRRNLKSLTTGECESLRSALHDMEEDGSFEAIAQFHGSPGLCDHK-GK---QV-GCCVHGAPTFPHWHRLFVEQVENALLDHGSAVSVPYWDWTEDFDHLPKLLAMPTYYNS : 1399
NlH2       : DVFGNASIVYKPGLGQMKGHAEDYLQEVRASSHIRQNIDSLTLGECESLRSALHDMEEDGSFEKIAKFHGYPGECQHD-GS---PS-SCCVHGSPTFPHWHRLYVENVENALLNHGSAVSMPYWDWTQPIVHLPKLFSLPTYYDS : 1389
RtH2       : DVFGNASIIFKSGRGHLKGHTEDYLREVRASSHIRRNIEALTKGECESLRSALHDMEEDGSFEKIAQFHGSPGLCTHD-NH---PA-ACCVHGSPTFPHWHRLYVENVENALLNHGSAVSMPYWDWTQPIEHLPALINEPTYYDS : 1390
LisaH1     : DLFGSSTTVY--SAGHQDGNA--YRQDVQASSHVRRNIDDLTEGEAESLRAALLHMEEDGSFEKIAQFHGFPGLCELN-GQ---KK-ACCVHGSPTFPHWHRLYVEQVENALLSHGSAVSVPYWDWTQPIRKLPELIDAPTYFNS : 1393
LisaH2     : DIFGVPTIVQTKGPGHVAGSP--YFKEVQASAHVRRNIEDLTEGETESLRAALLKMEEDGSFEAIARFHGYPGLCHPD-DDATANK-ACCVHGSPTFPHWHRLYVEQVENALLAHGSAVSVPYWDWTQPIKEIPDLIDAPTYFNS : 1386
MtH400     : DTFGQVTVVHTYCAGHVERKS--YGEELKASSHVRRNVDDLTGGEIESLKAALQKMEDDGSFENIAEFHGAPGLCELN-GK---KV-GCCVHGMATFPHWHRLYVEQVENALLSHGSAVSIPYWDWTKPITKLPDIIAQETYFDS : 1403
MtH550_a-f : DNFGKVTIVHTYGAGHLDRKS--YGQELKASSHVRQNVDSLSEGQKESLKAALQKMESDGSFEKIAEFHGAPGLCKHE-GR---NV-GCCIHGMATFPHWHRLYVEQVENALLTHGSEVSIPYWDWTSPIKELPDLVSKATYFDS : 1395
PcH_I      : NLFGNVTIVRDTGTGVRRGRV--FVNVIETASHVRKNLEDLSPGETESLRSALLQMQKDESFQKIASFHGFPGMCKHN-NR---SV-ACCVHGSPTFLHWHRLYVEQVENCLLARGSAVSVPYWDWTQPIRQLPTLISSPSFFNS : 1384
PcH_IIb    : EVFGKVTVVVRPGSETKKGEI---DKNIVASSHIRRNLQDLSEGEVESLKSALRRMQDDGTFGKIASFHGFPSLCTHE-GR---KV-ACCVHGSAVFPHWHRLYVDQVENALLARGSAVSVPYWDWTQPIRQLPALINEASYFDS : 1390
PcH_III    : STYGQVTIINEPCAGSRQEEI--YKREVQAASHVRRNIESLSKGEIESLRNALSKIEKDGTFENIAKFHGYPGLCEHE-GR---NV-SCCDHGSATFPHWHRLYVEQVENALLAQGSAVAVPYWDWTQPIRSLPDLINEAHFFNS : 1390
LsH1       : DTFSTPTVLFELGTSNLYGKE--FRAVSTAASQIRRNLDSLSQGEIESLRAAFLSIKEDGTYEKIASFHGKPGKCKDSAGR---TV-ACCVHGMPTFPAWHRLYVEQVEEALLGRGSSVAVPYWDWTTAFTKLPDLINQATYFNS : 1401
LsH2       : NVFPTPTILYDLGTSHLYGKE--YRKPVTAASQIRRDLETLSKGEIESLRSAFLSIKADKTYEKIASFHGKPGLCEHN-GN---KV-ACCVHGMPTFPAWHRLYVEQVEEALLSRGSSVAVPYWDWTKPITKLPELINEATYYNS : 1394
KLH1       : DLFKQPSVIHEPRIGHHEGEV--YQAEVTSANRIRKNIENLSLGELESLRAAFLEIENDGTYESIAKFHGKSPGLCQLN-GN---PI-SCCVHGMPTFPHWHRLYVVVVENALLKKGSSVAVPYWDWTKRIEHLPHLISDATYYNS : 1380
HtH1       : DLFHTANVVHDSGTGTRDRDN--YVEEVTGASHIRKNLNDLNTGEMESLRAAFLHIQDDGTYESIAQYHGKPGKCQLN-DH---NI-ACCVHGMPTFPQWHRLYVVQVENALLNRGSGVAVPYWEWTAPIDHLPHFIDDATYFNS : 1379
OdH-G      : GVHLETAIIYEPGLGN-FGEAGIWVEPVTSANRIRKNLNALTDGDMESLRKAFKDMTTDGRYEEIASFHGLPAQCPNKDGS---KVYTCCIHGMPTFPHWHRLYVALVENELLARGSGVAVPYWDWVQPFDHLPALVNRATYYNS : 1388
NpH        : EEF-SPTVLYVPALGT-YGTQTKWREPVTSASRIRKDLNTLTDGELESLRNAFLRLQEECAYEPIAAFHGVPAKCRGEDG----NIYTCCVHGMPTFPHWHRLYVIQVEHALRERGSGVSVPYWDWTKRFNHLPALMDESTFFNS : 1392

               1460          *      1480          *      1500          *      1520          *      1540          *      1560          *      1580          *
NlH1       : RMHRKEINPFFRWKV--SGSGEFTSRDPRFDLFDSEYFLESAMLALEQTSFCDFEVQFEVTHNAIHSFLGGRGKVSMSTLDYSAFDPIFMLHHSTVDRIWAIWQALQKHRDLSFDETDCAVNLMGQTMHPFDNTDINLFRLTHEY : 1543
RtH1       : RMHSKDVNPFFRWKV--SGTDEYTTRDVRFDLFNAEYFLDNILLALEQTSYCDFEVQFEVSHNAIHSFLGGRGKVSMSTLDYSAFDPIFFLHHANMDRIWAIWQALQKHRDLPFDETDCAVNLMSKPLHPFDNKDINHFGLTYKH : 1542
NlH2       : RTQHKEPNAFFRWKI--PDMDEYTSRDFQPELYDSHYFLDNVLLALEQTSFCDFEVQFEVVHNALHAFLGGRGKYSLSTLDFAAFDPVFFLHHANVDRIWAIWQALQKFRGGSFDETDCAMNLMATPLHPFDDKEMNDCRVTHKY : 1532
RtH2       : RTHKKEANAFFRWKV--PGTDEYTSRDPRPELYDSHYFLDNVLLALEQTSFCDFEVQFEVVHNALHSFLGGRGRYSMSTLDYSAFDPVFFLHHATVDRIWAIWQALQKFRGLPFDETDCAVNLMNIPLHPFDDKDLNDCWVTHKY : 1533
LisaH1     : RSHTKDTNPFFRGKI--SDTDKYTTRDPRPDLFNNNYFLDNVLLALEQTSFCDFEVQFEIVHNALHSWLGGRGKFSMSGLDYSAFDPVFFLHHANTDRIWAIWQALQRHRGLSFEESDCALNLMRKPLHPFDDKTQNEFDLTNHY : 1536
LisaH2     : RSQTKDPNPFFRWKI--SGLDEYTTRDPRPDLFDSSYFLDNVLLALEQTSFCDFEVQFEIIHNALHSFLGGRGKFSMSSLDYSAFDPVFFLHHANTDRIWAIWQALQRYRSLSYEESDCALNLMRQPLHPFDDKTLNEFDLTDAY : 1529
MtH400     : RSQTMQNNPFFRGPIRTPDVNDYTTRDPQPELFNNDYFLQQTLLALEQTSYCDFEAQFEVTHNAFHSFLGGRGKYSLSTLDYSAFDPVFFIYHANTDRIFAIWQALQSYRGLHWDEADCALNHMRDGLHPFDVKAQNEFDITHKY : 1548
MtH550_a-f : RSQTMETNPFFKAQI--GNTGHYTTRDPQPQLFNTKYFLNNVLLALEQTSFCDFEAQFEVVHNMMHSFVGGRGKYSLSTLDYSAYDPLFFIYHANTDRIWAIWQALQSQRGLPWDESDCALNHMDHLHPFDDKAQNEFDITHKY : 1538
PcH_I      : RTYNKESNPFFRGEI--PDTDAFTTRDPQPQLFNSNYFLDNVLLALEQTSFCDFEVQLEVTHNALHSWLGGRDKHSMSSLDYSAFDPAFFLHHANTDRLWAIWQALQQYRGLPSHESDCSLHLMRRPLQPFDSTSQNPVEITNQF : 1527
PcH_IIb    : RSQTEESNPFFQVKI--LDTGKVTTRDPQPELFNSDYLLNNVLLALEQTSFCDFEIQFEIVHNALHSLVGGRGLYSLSSLDYSAFDPLFFLHHANVDRIWAIWQALQQYRGLPDDKSDCALNIMRPLKPFNDKGQNEFDLTNRY : 1533
PcH_III    : RTQKEEPNPFFQGKI--SDVDEVTTRDPKPELFNSDYLLNNVLLALEKTGFCDFEIQFEIVHNSIHSWVGGRGQYSLSSLDFAAFDPVFFLHHSNVDRIWAIWQALQRYRGLPYDRADCAINAMQTPLRPFGDKGKNLFDVTHRY : 1533
LsH1       : RQQRFEPNPFFSGSI--AGEGAVTTRDPQPELFNNDDLYEQVLYALEQDHFCDFEIQFEIIHNALHSLLGGRAQYSLSSLDYSAFDPVFFLHHANTDRIWAIWQELQRYRGLKYNEADCAINQMRTPLRPFNDDQLNHGDLTHRY : 1544
LsH2       : REQRFDPNPFFCGTI--ANENAVTTRDPQPELFNSEELYEQVLLALEQEHFCDFEIQFEIVHNALHTLLGGRSQYSLSSLDYSAFDPVFFLHHANTDRIWAIWQELQRYRGLPYNEADCAINLMRKPLQPFNNDSLNHGDLTHRY : 1537
KLH1       : RQHHYETNPFHHGKI--THENEITTRDPKDSLFHSDYFYEQVLYALEQDNFCDFEIQLEILHNALHSLLGGKGKYSMSNLDYAAFDPVFFLHHATTDRIWAIWQDLQRFRKRPYREANCAIQLMHTPLQPF-DKSDNNDEATKTH : 1522
HtH1       : RQQRYDPNPFFRGKV--TFENAVTTRDPQAGLFNSDYMYENVLLALEQENYCDFEIQFEIVHNALHSMLGGKGQYSMSSLDYSAFDPVFFLHHANTDRLWAIWQELQRFRELPYEEANCAINLMHQPLKPFSDPHENHDNVTLKY : 1522
OdH-G      : RTLLVEPNPFFKGKI--SFLNSETNRDPQEELFGNKYLYEHTLFVLEQTDFCDFEVHFEVLHNTIHSWLGGRDPHSMSSLDFAAYDPIFFLHHSNIDRIWAIWQELQRYRKLPYNEANCALPLLNVPMRPFSNTTANHDRMTLTH : 1531
NpH        : RKNEMNTNPFHRGSI--SFINSHTSRDFQPELFNSDYLYENTLLALEQNDFCNFEVQLEVLHNAIHAWIGGRDTYSMSSLDYAAYDPIFFIHHSTLDRIWAIWQELQRYRGLPYNEANCALPLMNQHLRPFNNVSVNHDRNTLTL : 1535

               1600          *      1620          *      1640          *      1660          *      1680          *      1700          *      1720          *      1740
NlH1       : SRPADVWDYSGHLNYHYDNLDFHHWSISELDEVLKKQRSRDRMFAGFMLHGIGTSAVVEINVCVDT--G-VGGRSCHHPAGKFCILGGEYEMPFTFDRLYKHDISDAIRKLGLKLDSAADFDLEVKIHSFNGSYIDASLLKKPTI : 1685
RtH1       : SRPADVWDYSRHLDYHYDNLQLHGWSIAQLDEVLKKQRSRDRMFAGFLLHNIGTSAVVEIDVCVDT--G-DGGRSCHHPAGKFSILGGEYEMPFTFDRLYKHDITDAIRKLGLRLDSAADFDLEVKIHSYNGSYIDASLLHPPSI : 1684
NlH2       : SRPKDVFDYSHHFDYHYDTLEFNSWTIPQLEEVLKKQRSHDRLFAGFMLHSIGTSADVEIDICVHT--G-AGGLSCDHPAGKFCILGGEFEMPWNFDRLYKYDITDTVRQLGLPLDSAADFEVHVKLHAYNGSYIDATPLGKPKI : 1674
RtH2       : SRPKDVFDYSHHFDYHYDTLEFNSWTIPQLEEVLRKQRSHDRLFAGFRLHNIGTSADVEIDICVKS--G-GGALSCDHPAGKFSILGGEFEMPWIFDRLYKYDITDTVRKLGLPLDSAADFEVHIKLHAFNGSYIDATPLGHPTI : 1675
LisaH1     : SRPADLFDYSGHLDYHYDNLKFNSWTVPQLDEVLKQQRSRDRLFAGFLLHNIGTSADVEIDVCVAT--G-NGAQSCNHPAGKFALLGGEYEMPFTFDRLYKFDISDTVRKLGLRLDSAADFDLEIKIKSYNGSYIDSNLLGRPSI : 1678
LisaH2     : SRPVDVFDYSSHFNYHYDNLXFNSWTIPQLEEVLKKQRSRDRLFAGFLLHNIGTSADVEIYVCVDT--G-NGGKSCDHPAGKFALLGGEYEMPFTFDRLYKFDISDTVRKLGLRLDSAANFDLQIKIRSFNGSYINTDLLSKPSI : 1671
MtH400     : SRPVDVWDYSDHLQYHYDNLLLNDWTIPQLEEVLKTQRSRDRMFAGFLLHNIGTSADVEIHICVAT--G-NGDRNCNNPAGKFSILGGEYEMPFTFDRLYKYDISDTVRKLGLRLDSGANFDLKVQIHAYNGSYLDPSLLKEPTI : 1690
MtH550_a-f : NHPDDVWDYSDHLDYHYDTLELNGWSIAELDEVLKKQRSRDRMFAGFLLHDIGTSADVEIFICLAA--G-KNQRDCNHPAGKFSVLGGEFEMPFAFDRQYKFDISEALTKLGIRLGSNADFDLKVTVQSYNGSYLSSDLLDAPTI : 1680
PcH_I      : SRPADVFDYTNHFSYRYDNLLFNSWTLPQLEAVLRNQRSRDRLFAGFLLSNIGTSADVLIFICVAT--G-NGGRSCNHPAGEFSILGGETEMPFTFDRLYKYDISEPIRQLGLKLDSAAEFELEIKIRAFNGSYLEPDLVDKPSI : 1669
PcH_IIb    : SRPIDVFDYSNHFDYHYDTLNFNSWTLPQLEEVLKRQRAQDRLFAGFLLHNIGTSADVQLFICVAT--G-NGERNCNHPAGSFSVLGGETEMPFIFDRLYKYDISDPVRKLGLKLDSAGDIDLEIKIRAYNGSYLESSVLRRPSL : 1675
PcH_III    : SRPADVFDYSHHLDYTYDDLSLNSWSIPQLEEVLKKQRSRDRLFAGFLLHNIGTSADVHIRLCVAT--G-NGERNCNHDAGEFSILGGETEMPFIFDRLYKFDISEPVRKLGFKLDNPGNLDLDIKIQAFNGSYLDPSIIRRPSL : 1675
LsH1       : SRPADTFDYRNHFHYEYDTLEFNHMTVPQLEALLKKRKESGRVFAGFLLHNIGLSAVANIYVCVPS--GPRGKKSCNHPAGSFSILGGELEMPFVFDRLYKYDITKTVRDLGLKLDNAANFELRVDIKAVNGSLLDSDIIPKPTI : 1687
LsH2       : SQPAETFDYRNHFHYEYDTLEFNHLTIPQLDNLLKSHQKSGRVFAGFLLHNIGLTAVVDIYVCVPS--GAHNKQSCNHKAGSFSILGGELEMPFAFDRLYKFDITRAVRDLGLKLDNAANFELKVDIRAVNGSLLDSSIIPPPTI : 1680
KLH1       : ATPHDGFEYQNSFGYAYDNLELNHYSIPQLDHMLQERKRHDRVFAGFLLHNIGTSADGHVFVCLPT--G-EHTKDCSHEAGMFSILGGQTEMSFVFDRLYKLDITKALKKNGVHLQ--GDFDLEIEITAVNGSHLDSHVIHSPTI : 1662
HtH1       : SKPQDGFDYQNHFGYKYDNLEFHHLSIPSLDATLKQRRNHDRVFAGFLLHNIGTSADITIYICLPD--G-RRGNDCSHEAGTFYILGGETEMPFIFDRLYKFEITKPLQQLGVKLH-GGVFELELEIKAYNGSYLDPHTF-DPTI : 1662
OdH-G      : SAPNDVFDYQNVLHYKYDTLSFYDLTITQLDHLIEERKSHDRIFAGFLLHGVQASADIHVEICVPT--S-KHEENCAHDVGVFSVLGGKSEMPWQFASVFQYEITDQLKLLGLNQN--SHFRGVTEVTAVNGSSINSDIFPHPTI : 1671
NpH        : NKPNDAFDYQNHFHYRYDNLDFHGLSIPQLEHELHERQTHDRVFVGFLLHGIQASAYVRIYICVPTQRG-SSKENCNNYAGIFSILGGVTEMPWHFDRLYYYEITHALSNLGLSHR--SHFRIKTDINAVNGTHFDSHIFPDPTI : 1677
```

```
                    *        1760         *        1780         *        1800         *        1820         *        1840         *        1860         *        1880
NlH1        : IFQ--PGEGHTQD-DEGHPIRDLVRKSVWSLSPAEHRSLVLAMRSLQHDSSADGFQSLASFHALPPLCPYPEATERFACCIHGMATFPQWHRLYTVQFEDALRRHGPLVGIPYWDTVVPQSELPPFLHEATWDDPLFHANFTNPW : 1827
RtH1        : IFQ--PGEGKTQD-DEGHPISDLVRKSVWTLSPAERRSLVLAMKSLQADSSADGFQSLASFHAQPPLCPYPEATKRFACCVHGMATFPAWHRLYTVQFEDALRRHGALVGIPYWDTVVPQSELPPFFNDEVWDDPLFHANFPNPW : 1826
NlH2        : LFQ--PGEGKSQD-DHGHTHRNLVRKNVRNLSPAEHRSLVLAMKSLQADSSADGFQSLASFHAQPPLCPYPEASKRFACCIHGMATFPEWHRLYTVQFEDALRRHGSLVGIPYWDTVVPQDDLPALFNDEIWDDPLFHNNFTNPW : 1816
RtH2        : MFE--PGEGKSQD-DQGHTHRNLVRKSVRNLSPAERRSLVLAMKSLQEDSSADGFQSLASFHAQPPLCPYPEANKRFACCVHGMATFPEWHRLYTVQFEDALRRHGSVVGIPYWDTVVPQEDLPAFFNDEIWDDPLFHANFTNPW : 1817
LisaH1      : IFM--PGEGKHQD-EHGHSERRLVRKDAWAISPAERRSLVIALRNLQADDSADGFQSLAAFHAVPPLCPSPEASERFACCVHGQATFPQWHRLYTVQFEDALRRHGALVGIPYLNSLEPVSMLPVLMSDETWYDPLFNSDIPNPW : 1820
LisaH2      : VFL--PGDDHVQN-DVGRTERRLVRKSVRNLSPAERRSLVLAMRSLQADSSADGFQSLASFHALPPLCPYPEATERFACCVHGQATFPQWHRLYTVQFEDALRRHGATVGIPYWDTVVPQTTLPPFFTEGTWDDPLFHSNFPNPW : 1813
MtH400      : IFE--PGENTFQT-QDGKTQRNMIRRNVLTLSLAERRSLVLAMRRLQEDHSADGFQALASFHALPELCPYPEAAKRYACCVHGMASFPQWHRLYTVQFEEALRRHGSLVGIPYWDTSRPQKALPAFATDEKFTDPVLNVEFDNPW : 1832
MtH550_a-f  : IFS--PGNRKVQSDRPAAADGDLVRRNVWSLSFQERRSLVLALRSLQEDHSADGFQSLASFHAVPELCPYPEAPKRFACCVHGSPAFPHWHRLYTVQFEEALKRHGSLVGLPYWDTTDAQSFIPNFLTDEKFHDTVDDVDIPNPW : 1823
PcH_I       : IFL--PGTGKRQT-ANGRTENRLVRKNAWALSPNELRSMMWAMRLLQEDSSPNGFQALASFHALPPLCPYPEAPVRYACCVHGMPTFPQWHRLYLVQFEDALRRHSALVGIPYWDSVEPSGFHPYMFTNKTYQVPVHKFQWNNPW : 1811
PcH_IIb     : IFV--PGEDNSQD-QTGKTERRLIRKSVTSLTIGERRSLVLALQSLEEDSSATGFQALASFHAVPPLCPYPEANQRYACCVHGMATFPQWHRLYTVQFEDALRRRGALVGIPYWDTVNPQNSLPAFFNEPIYRDPVLDVDIPNPW : 1817
PcH_III     : IFV--PGTDRKQN-AVGKSEKRLVRKSVTSLSQSERRSLVLAMQSLQADNSASGFQALASFHALPPLCPYPEANKRFACCVHGMATFPQWHRLYTVQFEDELRRRGAIVGIPYWDTIGPQKSLPDFFNDAEYYDPVLNTTIPNPW : 1817
LsH1        : LFE--PGTGERQK-SSGEVTNYLVRKEINSLSPREVQSLYSAMEALQADSSADGWQSIASFHAIPPLCPSPSAENRYACCLHGMATFPQWHRLYTVQVEDALKRHGALVGIPYWDWSRVSDSLPHFIDDENFVDPVTGDQKANPW : 1829
LsH2        : IFV--PGTSERQE-AHGAVSSFVVRKNVDALSQREALSLYNAMRALQADSSADGFQAIASFHALPPLCPSPAAINRYACCLHGMATFPQWHRLYTVQVEDALRRHGSLVGIPYWDWTRQSKSLPEFFSDPTYPDPDGGDPVDNPW : 1822
KLH1        : LFE--AGTDSAHT-DDGHTEPVMIRKDITQLDKRQQLSLVKALESMKADHSSDGFQAIASFHALPPLCPSPAASKRFACCVHGMATFPQWHRLYTVQFQDSLRKHGAVVGLPYWDWTLPRSELPELLTVSTIHDPETGRDIPNPF : 1804
HtH1        : IFE--PGTDTHIL-DHDHEEEILVRKNIIDLSPRERVSLVKALQRMKNDRSADGYQAIASFHALPPLCPNPSAAHRYACCVHGMATFPQWHRLYTVQVQDALRRHGSLVGIPYWDWTKPVNELPELLSSATFYHPIRNINISNPF : 1804
OdH-G       : IYV--PKQDHSADIKSEEGNEYLVRKNVERLSLSEMNSLIHAFRRMQRDKSSDGFEAIASFHALPPLCPSPTAKHRHACCLHGMATFPHWHRLYVVQFEQALHRHGATVGVPYWDWTRPISKIPDFIASKRYSDPFTKIEDYNPF : 1814
NpH         : SFVPAPGSKNATH-YQEEEHHHFVRKEVSRLSIYEIHEVVYAFRRMQRDMSADGFEYIASYHADPPLCPNPKALHRYACCIHGMATFLHWHRLYVVQFEQSLHRHGSDIGFPYWDWTRPITHIPALLKDVNYTDPFTNSVVGNPF : 1821

                *        1900         *        1920         *        1940         *        1960         *        1980         *        2000         *        2020         *
NlH1        : HGADIEFN--HQHVARDFNMDQLAKKGPK-GYDTWSWKQFVFALEQEDFCDFEVQFEIAHNALHAWVGGHEEYSMGHLHFASYDPVFILHHSNMDRILALWQELQAFRGHDPYNINCALELMREPLKPFSFGPPYNLNSNTREHS : 1969
RtH1        : QGAEIEFN--HHKVARDFDMDQLAKKGPK-GYDTWSWKQYIFALEQEDYCDFEVQFEIAHNAIHAWVGGKEEYSMGHLHYASYDPIFILHHSNTDRIFALWQKLQKFRGHDPNNVNCALELMREPLKPFSFGSPYNLNDNTREHS : 1968
NlH2        : HGADIEFN--HQHVARDIDMDKLSKKGPK-GYDTWSWKQYIFALEQEGYCDFEVQFEIAHNAIHAWIGGDEEYSMGHLHYASYDPVFILHHSNIDRIFALWQELQEFRGHDPNEVNCALEMMREPLKPFSFGAPYNLNPITEEHS : 1958
RtH2        : NGADIDFN--HQKIARDINMDKLSKKGPK-GYDTWSWKQYIYALEQEDYCDFEVQFEIAHNAIHAWVGGHEEYSMGHLHYASYDPVFILHHSNTDRIFALWQELQKFRGHDPNEVNCALEMMREPLKPFSFGAPYNLNPTTKEHS : 1959
LisaH1      : AGADIEFK--GAKVARDVQSDRLAKKGPH-GYDTWSWKQYLYALEQEDYCDFEVQFEIAHNAIHAWLGGSETYSMAHLHYASYDPAFILHHSNTDRIFALWQELQRYRGHDPNEINCALELMREPLKPFSFGAPYNLNPTTTKYS : 1962
LisaH2      : AGAAIDFA--GESIHRQIDTEELGRKGPK-GYDTWSWKQYLYALEQENYCDFEVQFEIAHNAIHKWTGGSQVYSMGHLHFASYDPVFILHHSNTDRIFALWQELQKYRGHDPNEINCALELMQEPLKPFSFGAPYNLNPTTKEYS : 1955
MtH400      : LGADIEFE--NSHTEREPNLARLGEEGEH-GYDTWLYEQYLLALEQDNYCDFEVQFEIAHNAIHAWVGGSKIHSMGHLHYASYDPIFLLHHSSTDRIFAVWQELQRLRGKDPNEANCALEIMHEPLKPFSFGSPYNLNENTQRYS : 1974
MtH550_a-f  : LGADIEFE--NSHTERQFNMDKLRKEGPK-GYDTWSWEQVMLAFEQENFCDFEVQFEVMHNAIHAWTGGTSVHSMAHLHYASYDPIFMLHHSSTDRIFAIWQELQRLRGLDPNSANCDLETMHAPLKPFSFGSPYNLNPDTKRYS : 1965
PcH_I       : ESAAITFA--GKRTARDFQNDRLADSDG--GLGGWQWKQFVFALEQEDYCDFEVQFEIAHNAIHAWVGGSEEYSMGHLHYASFDPVFLLHHSSMDRIYAMWQELQRYRGLDPNEANCALQLVREPLKPFSFGSPYNLNPVTHQYS : 1952
PcH_IIb     : AGADIAFE--NTTVERDFKRNRLSDDGEH-GYDNWLWKQFLFALEQENYCDFEVQFEIAHNAIHAWVGGSKEHSMAHLHYASYDPIFLLHHSSTDRIYALWQELQRLRGHDPNEANCALELMREPLKPFSFGAPYNLDPITKEYS : 1959
PcH_III     : AGAAIKFR--NINVDREFNMERLRQEGEH-GFDTWLWKQYLFALEQENYCDFEVQFEIAHNAIHAWTGGSKEFSMAHLHFASYDPIFLLHHSSTDRIYAVWQELQRLRGHDPNEANCALELMREPLKPFSFGPPYNLDPVTKEFS : 1959
LsH1        : KKARIEFE--NSETEREVVSDRLFKRGPH-GWDTWLFNQALLALEQEDYCNFEIQFEITHNAIHSWVGGSKEHSLGHLHYASYDPAFFVHHSNTDRLWAVWQALQKYRGYDPNEANCALEQMKDPLKPFSFGPPYNLNPLTKEHS : 1971
LsH2        : YKANIEFE--HAVTERDVKEEFLFKVGKH-GWDTWLFNQALYALEQEDYCDFEVQFEITHNAIHSWLGGSKNYSLGHLHYASYDPAFFIHHSNTDRLWAVWQALQKYRGHDPNEANCALELMREPLKPFSFGPPYNLNQMTQDNS : 1964
KLH1        : IGSKIEFEGENVHTKRDINRDRLFQGSTK-THHNWFIEQALLALEQTNYCDFEVQFEIMHNGVHTWVGGKEPYGIGHLHYASYDPLFYIHHSQTDRIWAIWQSLQRFRGLSGSEANCAVNLMKTPLKPFSFGAPYNLNDHTHDFS : 1948
HtH1        : LGADIEFEGPGVHTERHINTERLFHSGDHDGYHNWFFETVLFALEQEDYCDFEIQFEIAHNGIHTWIGGSAVYGMGHLHYASYDPIFYIHHSQTDRIWAIWQELQKYRGLSGSEANCAIEHMRTPLKPFSFGPPYNLNSHTQEYS : 1949
OdH-G       : NQGQISFISEDTETKREVS-EYLFEHPVL-GKQTWLFDNIALALEQTDYCDFEIQLEIVHNAIHSWIGGKEEHSLNHLHYAAYDPIFYLHHSNVDRLWVIWQELQKLRGLNAYESHCALELMKVPLKPFSFGAPYNLNDLTTKLS : 1957
NpH         : NHGHLSFISPEVMTQRDVQ-DKLFKQPKL-GRNTWLHDNVILALEQTDYCNFEVQFEIVHNAIHAWVGGSQEHSLSHLHFAAYDPAFYLHHSNVDRIWVIWQELQKIRGLSAYEANCALELMNEPLKPFAFGSPYNLNEMTRKYS : 1964

                     2040         *        2060         *        2080         *        2100         *        2120         *        2140         *        2160         *
NlH1        : KPEEALDYHTHFHYQYDSLELQGMNVQRIQ-DYINKQKEKARVFAGFLLEGFGSSAHVAFSVCKEGG--ECTDAGFFDVLGGQLEMHWKFDRLYKYEITDVLAAKGLEVHDSFHIEVTITAPNSTVLPSTLIPTPSIIYVPKSHD : 2111
RtH1        : KPEETLDYHTHFHYDYDSLELQGMDVHRLQ-DYINQQKEEDRVFAGFLLQGIGTSAHVAFSVCKDGG--ECTSAGFFDVLGGALETPWRFDRLYKYEISDVLKEKGLEVRDAFHIELTITASNGTALPSSLIPEPSVIYAPKSHD : 2110
NlH2        : KPEDSFDYKGHFHYEYDQMELQGMTAERLQ-DYINKQKERDRVFAGFLLGGIETSALVSFDVCKPDG--DCTHAGDFDILGGSLEMPWQFDRLYKYEITDVLAHKGLDVHDVFDIKVTITGPHSEDISSDKLPHPTVLFVPKSRD : 2100
RtH2        : KPEDTFDYKGHFHYEYDHLELQGMNVQRLH-DYINQQKERDRVFVGFLLEGIGTSAHLDFSICKPDG--ECTHAGYFDVLGGSLETPWQFDRLYKYEITDVLESKGLDVHDVFDIKITSTSWDNEDISTDRFPPPSVIYVPKSRD : 2101
LisaH1      : KPEDAFDYQAHFNYEYDKLELQGLDMSRIQ-DYINERKEKDRVFAGFLLHGFGTSAHVEFAVCNEEG--TCTPAGDFNVLGGSAEMPWRFDRLYKYEITDVLESKGLDVRDTFHIEVMVTAANGTVLDSHILPDASVIYDPKSRD : 2104
LisaH2      : RPEDAFDYQGHFKYEYDTLELQGLDIVRLQ-DYINQRKEEDRVFAGFLLRGIGTSAHVSFSVCAHGT--DCEEAGSFDVLGGSAEMPWRFDRLYKYEITDALEARGMDVRDAFHISVTITAQNRTVLDSSLIPEPSVIYEPHSRV : 2097
MtH400      : RPEDIFDYQSHFHYEYDSLEVLGMSPAQLE-DAIHKRQEKDRVFAGFLLKGIGTSAHVEFTICNEAG--VCTTAGDFDILGGSAEMPWSFDRAYKYDITNVLEKKGLEATDAFKVKVAITAQDGTALDSSLLPEPSIILQHATRD : 2116
MtH550_a-f  : SPDDIFDYHGHFHYEYDDLEMQGLSPPQIL-DRIAQRKERERVFAGFLLHGIGTSAHVEFSVCKD-N--ACTPAGDFNVLGGYAEMPWSFDRAYKYEITDVLKKKNIDPRDQFTIELKITALNGTTLSGNAFGEPSIIIQARSAE : 2106
PcH_I       : RPEDVFDYKARFNYQYDTLELLGMDVPRLQ-GYINKQKEKPRVFAGFLLHSLGTSAHVTFSVCSGEEYQECTLAGDFNVLGGSAEMPWRFDRLYRYEITDVLKTKGLKVDDMFQIKVVITAQNGTVLDSNSLPQPTVIFMPKIRE : 2096
PcH_IIb     : KPEDVFDYKSHFNYEYDTLELAGINAELLL-SLINKQKEKSRAFAGFLLGGIKTSAHVDIKVCTPED--VCKDAGDFDVLGGTAEMPWSFDRLYRYEITEVLEKKGLSVDDAFTVKVELRALNGSILSSSLIPTPSVLYEPKSRV : 2101
PcH_III     : KPEDVFNYKDHFLYTYDILDVQGVSVDLLQ-NYINRQKEKGRVFVGLLLGGIRTSAHVEIIVTAPGG--KDTPAGDFNILGGSAEMPWSFDRLYRYEITDVLKKAGLKKTDSFNVSVVITAINGTILDSSLIPTPSVIYKPAKRK : 2101
LsH1        : RPEDTFNYADHFHYRYDNLEFVGLSIPQLD-AFIKERHEHDRVFAGFLLKGFKKSALVDFDICAN-G--NCFPGGYFTVLGGSAEMPWQFDRLFKYEITDQLNEHKLRFDDEYTFQIKIRAPDGTELSSDLIGTPSVILEPFKRD : 2112
LsH2        : RPEDTFNYEDHFHYRYDDLQFVGMSIPRLD-AFIKEKQEHDRVFAGFLLKGFGQSALVSFEICNDDMS-NCFEGGYFTVLGGSQEMPWRFDRLYKYEITEQLEEHNLRYDDNYHFHVHVKNIDGTMLPGKLIPDPTVILVPAQSD : 2107
KLH1        : KPEDTFDYQ-KFGYIYDTLEFAGWSIRGID-HIVRNRQEHSRVFAGFLLEGFGTSATVDFQVCRTAG--DCEDAGYFTVLGGEKEMPWAFDRLYKYDITETLDKMNLRHDEIFQIEVTITSYDGTVLDSGLIPTPSIIYDPAHHD : 2089
HtH1        : KPEDTFDYK-KFGYRYDSLELEGRSISRID-ELIQQRQEKDRTFAGFLLKGFGTSASVSLQVCRVDH--TCKDAGYFTILGGSAEMPWAFDRLYKYDITKTLHDMNLRHEDTFSIDVTITSYNGTVLSGDLIQTPSIIFVPGRHK : 2090
OdH-G       : KPEDMFRYKDNFHYEYDILDINSMSINQIE-SYIRHQRDHDRVFAGFLLSGFGSSAYATFEICIEGG--ECHEGSHFSVLGGSTEMPWAFDRLYKIEITDILSDMNLAFDSAFTIKTKLVAQNGTELPASILPEATVIRIPPSNE : 2099
NpH         : LPEDTFNYKDHFDYEYDMLEFSGMTIPQLE-DYIKESLHHERIFAGFLLHGIGQSANVDFEVCDNQN--HCEDAGYFSILGGSLEMPWAFNRLYRYDITHTLETMHLNYDDNFHINIHIHDMAGNDLDSHLLQKATILRQPAAEH : 2106
```

```
                  2180        *        2200        *        2220        *        2240        *        2260        *        2280        *        2300        *        2320
NlH1        : TELHEVAPNRVRRSLADLTERDAKNLKSAIRDLQKDDSKDGYQHIAGFHGAPALCPTPEAAEYACCIHGMPTFPHWHRLYAVEMEDALIRHGSGVALPYWDWTLPITELPHLFTSESYYDPWRDEVVPNPFVRAYIKVAGGYTVR : 2256
RtH1        : TELHEVAPNRVRHDLADLTDRDIKNLKSAIRDLQNDISKDGYQHIATFHGAPALCPDPNAPTHACCLHGNPTFPHWHRLYAVEMEDALIRHGSGVALPYWDWMKPITELPELFTSESYYDPWRDEVVPNPFVRAYIKAANGYTVR : 2255
NlH2        : IEQHEVAPNRVRHELGDLSDRDVQNLKAAIRDLQLDDSRDGYQNIASFHGSPALCPSPDAPEYACCNHGNPTFPHWHRLYAVEMEDALIRHGSGVALPYWDWTYPLTKLPDLFTSESYYDAWRDEVIPNPFVRAYIDVAKGYTVR : 2245
RtH2        : IELHEVAPNRVRNDLSKLSERDIQNLKSAIRDLQLDKTIDGYQNIASFHGAPALCPSAENAEYACCIHGVPTFPHWHRLYTVEMEDALIRHGSGVALPYWDWSVAITDLPDLFTSESYYDAWRDEVIPNPFARSYIDVAKGFTVR : 2246
LisaH1      : SQLHEVAPNHVRHDLSHLSERDVMSLKAAMLDMQRDKGTSGYQNIAAFHGAPAMCPTPEAAEYACCVHGMPTFPHWHRLYTVEMEDALIKHGSSVALPYWDWTLPIEHLPDLFTKETYYDAWRDEVMDNPFARGYVSSVGGFTVR : 2249
LisaH2      : TALHEVAPNRVRHDLTHLKERDFQSLKAALRDLQNDDGPDGYQAIARFHGAPPLCPSPEAPTHACCIHGSPTFPHWHRLYTVEMEDALIRHGASVALPYWDWTKPIKDLPELFTSESYYDAWRDEVIINPFARCFIKSVNGYTVR : 2242
MtH400      : QGQYELAPNRVRRSLSDLTERDVMSLKSALHDLQEDDSATGWQSLAAYHGVPALCPSPEEAKYACCIHGMPTFPHWHRLYVLAVEHSLIKHGSSVAIPYWDWTNPLDKLPELFTKQTYYDAWRDEVYDNPFARGYVKGDEAYTVR : 2261
MtH550_a-f  : RGQYNQPANRIRHDLTHLSERDFMSLKSALNDLQADTGKMGWQSLASFHGVPALCPTPAEATYACCIHGMPTFPHWHRLYTLLVEHALIEHGSSSAIPYWDWTQPLDGLPALFTEQTYYDAWKDKVFDNPFARGYIKSVDGYTVR : 2251
PcH_I       : RDLQDAPPSRVRRDLRDLKEVDIQNLKAAMASFQRDKGGNGWEAITAFHGVPARCPSPQKPEKACCIHGMPTFPHWHRLYTLQVDMSVVRKGSSVALPYWDWTLPTDPLPSLFTEQTFYDAWKDEVLENPFARGFIKEISGYTVR : 2241
PcH_IIb     : TSIQELPPNRVRHEISHLDARATMSLKAALRDLQKDGTKSGWQALAAFHGVPALCPSPEAPKYACCLHGVPTFPHWHRLYTLQVEQALLKHGSTVALPYWDWTLPISQLPSLFTEQTFYDAWKDEVLENPFARGYIKESKGYTVR : 2246
PcH_III     : SSISQLPANRIRRDLSTFDVRDKMTLKAALKDLQKENN--GWEDLASLHGVPVKCP-VESPKVACCIHGMPTFPHWHRLYTLQAERELLKHGSSVALPYWDWTKPITELPELFTQQTYYDPWRNAVEENPFARSYISSASGYTVR : 2243
LsH1        : YDLEKVTPNFVRHDLSTLTERDLQGLKSALRDLQLDESADGWASLASFHGAPSMCENESGAKVACCIHGMPTFPHWHRLFTVQVERALQRHGSAVAIPYWDWTKPITALPEIFTKEDFYDAWRDEVVDNPFARGFIPTENTYTVR : 2257
LsH2        : HDLDNVTPNLIRRDLSTLSERDIQSLQAALRDLQLDQSNDGWASLASFHGAPNRCPDPANPTVACCQHGMPTFPHWHRLFTVQIEQALQRHGSAIAIPYWDWTKPIDELPKIFTDEDFYDVWRDEVVANPFARGYVPSEKVYTVR : 2252
KLH1        : ISSHHLSLNKVRHDLSTLSERDIGSLKYALSSLQADTSADGFAAIASFHGLPAKCNDSHNNEVACCIHGMPTFPHWHRLYTLQFEQALRRHGSSVAVPYWDWTKPIHNIPHLFTDKEYYDVWRNKVMPNPFARGYVPSHDTYTVR : 2234
HtH1        : LNSRKHTPNRVRHELSSLSSRDIASLKAALTSLQHDNGTDGYQAIAAFHGVPAQCHEPSGREIACCIHGMATFPHWHRLYTLQLEQALRRHGSSVAVPYWDWTKPITELPHILTDGEYYDVWQNAVLANPFARGYVKIKDAFTVR : 2235
OdH-G       : DADIDTPLNHIRRNVESLDERDIQNLMAALTRVKEDESDHGFQTIASYHG-STLCPSPEEPKYACCLHGMPVFPHWHRVYLLHFEDSMRRHGSSVATPYWDWTQPGTKLPRLLADSDYYDAWTDNVTENPFLRGYIKSEDTYTVR : 2243
NpH         : HEVHH-PLNRIRHNINDLSERDIQSLKSALIKVKEDHSNFGFQTITSYHGLPASCPTPEEATYACCMHGMAAFPHWHRLYALQFEESLLRHGSSVALPYWDWTHKITGLPHLLSNPDYYDALHKQVTENPFLRGHIEVAETYTVR : 2250
```

```
                          *        2340        *        2360        *        2380        *        2400        *        2420        *        2440        *        2460
NlH1        : DPQPELHKLSKDGNHSILFDEILLALEQTDYCDFEVQYEVTHNAIHYLVGGRQVYSLGSLDYSSYDPIFFVHHAFMDKVWAVWQEMQKRRGLVYDRADCAVNYMKHKMHPFDWDDLNPDVRTREHAMPQSVFDYEDLGYHYDNFE : 2401
RtH1        : DPRPALKKLSHDGKHSILFDEVLLALEQTDYCDFEVQFEVTHNAIHYLVGGRHLYSLSSLEYSSYDPIFFVHHSFVDKIWAVWQELQKRRGLTYDRADCAVNFMQHKMHPFDWDDLNPDVRTREHAHPQTVFDYEDLGYHYDDFK : 2400
NlH2        : NVQPELTKLSKDGKHSVLFDELLLVLEQTDYCHFEVQYEVLHNAIHYLVGGRQVYSLATLDYASYDPIFFVHHSFVDKVWAVWQELQKRRGMVYDRADCAVNYMNHPMHPFDWESLNPDVRTREHASPQTVFDYEDLGYHYDNFE : 2390
RtH2        : DPQPELTKLAKDGKHSMLFDELLLALEQTDYCDFEVQFEVLHNAIHFLVGGRQEYSLSSLEYSSYDPIFFVHHSFVDKVWAVWQELQKKRGLVYDRVDCAVNYINQPMHPFDWENLNPDVRTREHASPSSVFDYEDLGYHYDNFE : 2391
LisaH1      : DPQPELTKLSRDGKHSVLFDEVLLALEQTDFCDFEVQYEVLHNAIHYLVGGRQTFALSSLEYSSYDPIFFVHHSFVDKIWVIWQEMQKHRGLSYDRADCAVNYMNEVLHPFDWAQFNTDVRTRAHSLPQSVFDYEDLGYHYDNLE : 2394
LisaH2      : DPQPELTKLSRDGQHSILFDEVLLVLEQTDYCDFEVQYEVTHNAIHYLVGGRQTFALSSLEYSSYDPIFFVHHSFIDKVWVIWQELQKRRHLSHDRADCAINFMNQKLHPFDWEELNPDVRTREHALPQTVFDYEDLGYHYDNME : 2387
MtH400      : DPQPELKERSRDGKHSVLFDQVLLALEQTDYCDFEVQYEVTHNAIHYLVGGPQTFSLSSLHYSSYDPIFFVHHSFVDKIWAVWQALQRKRGKPSERADCAVNYMYDDMHPFDSAKLDPDPTIRSHANPSTVFNIYDLGYKYDDYQ : 2406
MtH550_a-f  : DPQSQMSMRSKDGKHSILFDRVLDALEQDDFCDFEVQFEVTHNAIHYLVGGPQTYSLSSLHYSSYDPIFFVHHSFVDKIWAVWQELQRRRGKPYDRADCAVNYMSKPMMPFNSPDLDPDLEVRAHAVPNSVFNYFELGYKYDNLE : 2396
PcH_I       : DPQPELLKLSADGEHSVLFDEVLLVLEQTDYCDFEVQFEVVHNAIHYLVGGRQSYSLSSLHYASYDPLFFIHHSFVDKIWAVWQELQKRRHLPHDRADCAVNFMAEPMAPFNNPKVNFNPRTRAYAVPQTVFDYEGLEYTYDNLN : 2386
PcH_IIb     : DPQPELFKLSADGKHSVLFDEVLLVLEQTDYCDFEVQFEVTHNAIHYLVGGHQSYSLSSLHYSSYDPIFFTHHTFVDKIWAVWQELQKRRGLPYDRADCAVNYMSDAMQPFNSVVLNENAFTRTHALPKEVFNYQDLNFRYDDMK : 2391
PcH_III     : DPQPELFKTTPDGKNSILFDEVLLALEQTDYCDFEVQFEVTHNAIHYLVGGHQTYALSSLSYASYDPIFFVHHSFVDKIWAVWQELQKRRNLPYNRANCAVNFVTKPMLPFSSPSLNKDKFTLEHSTPLDVFDYLSLNYAYDNLD : 2388
LsH1        : DIRPELFQTSKDGSHSDLFELVLSALEQTDYCDFEVQFEVMHNAIHYLVGGLQTYSLSSLEYSAYDPIFFIHHSFVDKIWVVWQELQQRRHLPSNTANCALNYLAEPMKPFNLD-LNINAFTKKHAVPRDVFNHEELGYHYDNLN : 2401
LsH2        : ETKSELYDKGRGGKNSALIYYVISALEQTDYCDFEVQFEVIHNSIHYLVGGEQTYSLSSLEYSAYDPIFFIHHSFTDKIWAVWQELQKRRRLPYLKANCALNYMAEPMKPFSIDGLNLNKFTKEHAVPDKLFDYEELGYAYDTLE : 2397
KLH1        : DVQEGLFHLTSTGEHSALLNQALLALEQHDYCDFAVQFEVMHNTIHYLVGGPQVYSLSSLHYASYDPIFFIHHSFVDKVWAVWQALQEKRGLPSDRADCAVSLMTQNMRPFHYE-INHNQFTKKHAVPNDVFKYELLGYRYDNLE : 2378
HtH1        : NVQESLFKMSSFGKHSLLFDQALLALEQTDYCDFEVQFEVMHNTIHYLVGGRQTYAFSSLEYSSYDPIFFIHHSFVDKIWAVWQELQSRRHLQFRTADCAVGLMGQAMRPFNKD-FNHNSFTKKHAVPNTVFDYEDLGYNYDNLE : 2379
OdH-G       : DVKPELFEIGG-GEGSTLYQQVLLMLEQEDYCDFEVQFEVVHNSIHYLVGGHHKYAMSSLVYSSFDPIFYVHHSMVDRLWAIWQALQEHRHLPFDKAYCALEQLSFPMKPFVWD-SNPNLHTRAASTPQHLFDDNKLGYKYDNLE : 2386
NpH         : NTQPELFEPSKDGKHSLLYDEILLALEQRDYCDFEVQFEVVHNAIHYLVGGHQRYSMSSLEYASYDPLFFIHHSNVDRLWAIWQALQQYRRIPYNKAYCALEQMTHAMKPFDRE-TNPNKNTHDHAIPNSVFNYDDLGYSYDNLL : 2394
```

```
                     *        2480        *        2500        *        2520        *        2540        *        2560        *        2580        *        2600        *
NlH1        : IGGNTLEELERKIHEKQSHPRVFAAFHLHSIGTSADILFHVCKTEN--SCTRAGGIFVLGGALEMPWSFDRLYKYDITEDLHDLGIEPEDVFNAQA-PFHLKYEIHAVNGSALPP-SSISAPTLIFKPAVGASVDQT-SYSIAGV : 2541
RtH1        : LGGNTLEELEEMIHEKKSHPRVFAGFHLHNIGTSADVVFHICTTES--RCTRAGGLFILGGALEMPWAFDRLYKYDITEDLHDLGIEPEDVFNVQA-PFHLKYEIHAVNGSTLAP-STISAPTLIFKPAEGASVDQT-SYSIAGV : 2540
NlH2        : LGGNDLDSLEKLIHEQKSHPRVFAGFHLESIGTSAGVIFHVCRKDS--SCVRAGGIFILGGKLEMPWAFDRLHKYDITSVLHDIGIEPEDVFNPQA-PFFLKYEIQGVNGSSLPL-STVAAPTLIFKPAEEAKSEEV-SHAIAGV : 2530
RtH2        : LGGNDLEKLEELIHEQKSHARVFAAFHLHNIGTSAAVVFQACRTES--NCVRAGGLFILGGKLEMPWSFDRLFKYDITSVLHDIGIEPEDVFNPEA-PFFLKYEIQGVNGSSLPL-STVSPPTLVFKPAEEAQADEA-AHSIAGV : 2531
LisaH1      : LGGKTLEELEELIHDQQSHPRVFAGFHLHAIGTSADVIFEVCKTDD--ECYRAGGFFILGGHLEMPWTFDRLYKYDITDTLHDADISPEDVFNAQT-PFTIKYSIHAVNGSALPL-SSVSPPTIIFEPAHGASQDHT-SYSVAGV : 2534
LisaH2      : LGGKTLDEIEELIHERHSHPRVFAGFFLHAIGTSADVTFSICKTDT--SCVRAGAFFILGGHLEMPWRFDRLFKYDITDALHDANIEPEDVFNAEA-PFTIEYEIHAVNGSALPK-SSISAPTIIFVPAEGSDAEAS-SYSIAGV : 2527
MtH400      : IGGKNLDELEALIKDNQAHPRVFAGFHLKGIGSSADVVFKICKDKSGGQCTRAGSIFILGGEKEMPWEFDRLYKYDITDALKDNGIQPEDVFDAEA-PFYLKYEVTAVNGSTLPK-SVINAPTLIFVPATGASVDAK-SYAVAGM : 2548
MtH550_a-f  : IGGESLEQLEDRIRDQQSHARVFAGFYLGGIGTSAGVTFAVCRKDDGKRCTYGGSFFILGGSKEMPWSFDRVYKHDMTRALHAIGLEPEDVFDYKNYNVFISYNVTAVNNTALPR-SIIPKPTILYEPAKV-------------- : 2525
PcH_I       : IGDKTPDELEKLINKQRSRPRVFAGFLLHGIGTSADVVISVCVHSN---CVRAGAFFILGGALEMQWSFDRLYRHDITQALLAVSVRPQDLFTEHT-PFTLDYSITAVNGTQLPR-SLMPPPTIIYEPASGGFRGMP-SYSLAGI : 2525
PcH_IIb     : LGGLNLDELEEEIRQHQSKARVFAGFLLHGIGTSAEVRFSVCQENG--KCTRAGAIFVLGGSLEMPWAFDRLYKYDITDTLNELHIRPEDVFNADA-PFSLRYEIIAVNGTALPP-SSLSKPTIIFKPAVGARTEEA-SYSVSGI : 2531
PcH_III     : LGGHTLEEIEHDIDEHQSKPRIFAGFLLHGIGVSAEVRCSICVTRT--RCARAGAFFVLGSDVEMPWSFDRLYKYDITQALEALSIRPEDVFDAQA-SFNLNVDIIAVNGTKLPS-SKLPTPTIIFEPAKGATAERQ-SYELSGV : 2528
LsH1        : IGGYNLDQLEQLIAQHQSRPRVFAGFLLKGIKTSGSVVLNVCKGSQ---CTYAGRFNLLGGPSEMPWAFDRLYKKDITWALEAADIKPESVFDADS-LFTLDVKVFDVEGHAINVKSVFPTPSIIYDPAKGVANVDI-TSAVAGV : 2541
LsH2        : IGGYSLEQIEEIIADNKKTARVYAGFLLKGIKTSGSVIIKACKDSE---CAFAGRFNLLGGPTEMPWAFDRLFKADITWALRNLGLTADDVTDVDS-HFHLEVDITDVKGRSLNRKSVMPEPTIIFRPAEGSTQDAV-STSIAGV : 2537
KLH1        : IGGMNLHEIEKEIKDKQHHVRVFAGFLLHGIRTSADVQFQICKTSE--DCHHGGQIFVLGGTKEMAWAYNRLFKYDITHALHDAHITPEDVFHPSE-PFFIKVSVTAVNGTVLPA-SILHAPTIIYEPGLDHHEDHH-SSSMAGH : 2518
HtH1        : ISGLNLNEIEALIAKRKSHARVFAGFLLFGLGTSADIHLEICKTSE--NCHDAGVIFILGGSAEMHWAYNRLYKYDITEALQEFDINPEDVFHADE-PFFLRLSVVAVNGTVIPS-SHLHQPTIIYEPGEDHHDDHQ-SGSIAGS : 2519
OdH-G       : FHGMNIDQLENAIHKQQNKDRVFASFLLFGIKTSADVHLKLCKDET---CEDAGVVFILGGDNEMPWHFDRTYKKDITHVLHQMHIPLEDLYVHGS-TILLEVEIETVDGKVLDS-SSLPAPSMIYVPAKDFKREVH-KKTVGDA : 2525
NpH         : FDGLTITELEEKIHSRQSHPRVFAGFLLHAIGTSADVHVSVCHHGN--NCTSAGLFFILGGKHEMPWKYDRLFRYDITDAVTKAGFSPEDVFNDNL-LYKIHTEINAVNGSHIDS-HIMHEPTLIFVPGRNTKQAERERRISGGP : 2535
```

```
                  2620         *       2640         *       2660         *       2680         *       2700         *       2720         *       2740         *
NlH1        : GVRKDINTLTAAEVSNLRDALRRLQAGTGRVSYDSVASAHGYPPQCKM------------------------------------------------------------------------------------------------- : 2589
RtH1        : GVRKDINTLTAAEMKNLRDALRRVQAGTGRLTYDFIAGAHGYPAACKM------------------------------------------------------------------------------------------------- : 2588
NlH2        : GVRKDINSLTAEEKENLREAMRRVQAGTGRMSYNTVVKAHGYPPQCRLKVHGHKGHDDHHDDHDDDHHDDHHPNGHKKHGHHDDDHHDDHDDDHHNNSSHHGHKKHGHHDDDHHDDHDHHGHKKHGHHDDDHHDDHDHHSHKKHG : 2675
RtH2        : GVRKNINSLTDAEMENLREAMRRVQAGTGRLSYNKVAKAHGYPAQCHLK------------------------------------------------------------------------------------------------ : 2580
LisaH1      : GVRKDINTLTAEETESLRDSLREMQEGGSGLPFQQFASWHGWPASCEH------------------------------------------------------------------------------------------------- : 2582
LisaH2      : GVRKDINTLTAAETENLRDALRHAQAGTGRLPYNEVAKAHGYPPQCEH------------------------------------------------------------------------------------------------- : 2575
MtH400      : SVRKDINTLSSAETQSLKNSLQKVMDGEGPLSYQKIAEMHGWPGDCEQ------------------------------------------------------------------------------------------------- : 2596
MtH550_a-f  : ------------------------------------------------------------------------------------------------------------------------------------------------- :    -
PcH_I       : GVRKDINTLSEAETENLREALSSVMDDTRIISYQRLAGWHGFPGLCSM------------------------------------------------------------------------------------------------- : 2573
PcH_IIb     : GVRKNINTLTVAETESLKKALRAVQQQSGPRGYQEIAAAHGYPGLCKD------------------------------------------------------------------------------------------------- : 2579
PcH_III     : GVRKNINTLTVAETENLKKALQAVKDDKGVSGYQNIAAAHGYPGKCTE------------------------------------------------------------------------------------------------- : 2576
LsH1        : GVRKDVSSLTASETESLRNALRKVQEDEGPNGFQNIASFHGSLAKCEY------------------------------------------------------------------------------------------------- : 2589
LsH2        : GVRKDVSTLTVSEIENIRNALKRVQEDDGPNGFQNIASFHGSPAKCEY------------------------------------------------------------------------------------------------- : 2585
KLH1        : GVRKEINTLTTAEVDNLKDAMRAVMADHGPNGYQAIAAFHGNPPMCPMP------------------------------------------------------------------------------------------------ : 2567
HtH1        : GVRKDVNTLTKAETDNLREALWGVMADHGPNGFQAIAAFHGKPALCPMP------------------------------------------------------------------------------------------------ : 2568
OdH-G       : IIRNNVNSLTPSDIKELRDAMAKVQADTSDNGYQKIASYHGIPLSCHYE------------------------------------------------------------------------------------------------ : 2574
NpH         : IIRKNINKLTSSEIHELREAMAAVQADHSSNGYQAIAAYHGLPLQCK-------------------------------------------------------------------------------------------------- : 2582

             2760         *       2780         *       2800         *       2820         *       2840         *       2860         *       2880         *       2900
NlH1        : ------------------------------------------------------------------------------------------------------------------------------------------------- :    -
RtH1        : ------------------------------------------------------------------------------------------------------------------------------------------------- :    -
NlH2        : HHDDDHHDDHDHHSHKKHGHHDDDHHDNHDDHHHNDSRHNGHKKDGHHDDHHDDHRGDHHDDHRHNRHRNKRNGNRHDHHDDHNDDHHHKNGHHDDRHDHHDGDHHDHDGDHHDHHDHHDHGNDHDDHRHNGHKKSGHHDDHHDD : 2820
RtH2        : ------------------------------------------------------------------------------------------------------------------------------DDHHDDHHHDDHGHRVLRA : 2599
LisaH1      : ------------------------------------------------------------------------------------------------------------------------------------------------- :    -
LisaH2      : ------------------------------------------------------------------------------------------------------------------------------------------------- :    -
MtH400      : ------------------------------------------------------------------------------------------------------------------------------------------------- :    -
MtH550_a-f  : ------------------------------------------------------------------------------------------------------------------------------------------------- :    -
PcH_I       : ------------------------------------------------------------------------------------------------------------------------------------------------- :    -
PcH_IIb     : ------------------------------------------------------------------------------------------------------------------------------------------------- :    -
PcH_III     : ------------------------------------------------------------------------------------------------------------------------------------------------- :    -
LsH1        : ------------------------------------------------------------------------------------------------------------------------------------------------- :    -
LsH2        : ------------------------------------------------------------------------------------------------------------------------------------------------- :    -
KLH1        : ------------------------------------------------------------------------------------------------------------------------------------------------- :    -
HtH1        : ------------------------------------------------------------------------------------------------------------------------------------------------- :    -
OdH-G       : ------------------------------------------------------------------------------------------------------------------------------------------------- :    -
NpH         : ------------------------------------------------------------------------------------------------------------------------------------------------- :    -

                    *       2920         *       2940         *       2960         *       2980         *       3000         *       3020         *       3040
NlH1        : --------------------------------------------------------------------------------------------------GPYDIACCRHGMASFPGWHRVFTRQMEVALSWEGAMVGIPYWEWTEA : 2636
RtH1        : --------------------------------------------------------------------------------------------------GEYDVACCQHGMASFPGWHRVFTRQMEVALSWEGAKVGIPYWDWTEA : 2635
NlH2        : HDDHHDDHDRDHHHNGHKKSGHRDDHHDDHDDHHDDHDRDHHHNGHKKNGHRDDHHDDHHDDHHDDHHDDHDEDHHKKNGHHDDHDDHHDDHDEEADYYYREVACCHHGMASFPQWHRVFVKQMEAALTWEGAKLGIPYWDWTEA : 2965
RtH2        : VHDHDDHGHPALRAVHGHEVHHDDHGHEVHHDDHGHEVHHADHGHEDHHDDHGHAAPFADHGHDDHHDDHGHEVHHDDHGHEVHHADHGHEVHHDDQHGKKEVACCHHGMASFPQWHRIFTKQMEAALMWEGAELGMPYWDWTES : 2744
LisaH1      : --------------------------------------------------------------------------------------------------DGHPVACCHHGMASFPQWHRLYVRILEMVMTFRGARVGIPYWDWTQA : 2629
LisaH2      : --------------------------------------------------------------------------------------------------DGHEVACCQHGMASFPQWHRLFTRQMELALFWEGAKVGIPYWDWTEA : 2622
MtH400      : --------------------------------------------------------------------------------------------------NGKKVACCHHGMASFPQWHRVYTRLLEMAMTWQGANVGIPYWDWTEA : 2643
MtH550_a-f  : ------------------------------------------------------------------------------------------------------------------------------------------------- :    -
PcH_I       : --------------------------------------------------------------------------------------------------NGQQVACCQHGSASFPHWHRLYVRSLEIAMTLEGARIGIPYWDWTTT : 2620
PcH_IIb     : --------------------------------------------------------------------------------------------------NGHTVACCHHGSASFPQWHRLFVVTLERALTLQGARVGIPYWDWTES : 2626
PcH_III     : --------------------------------------------------------------------------------------------------SGRTVACCHHGMASFPQWHRLFVVLLERALTRQGARVGIPYWDWTES : 2623
LsH1        : --------------------------------------------------------------------------------------------------QGHPIACCLHGMANFPQWHRLYVKQWEDALTSQGSKVGIPYWDWTTA : 2636
LsH2        : --------------------------------------------------------------------------------------------------QGHPIACCLHGMANFPQWHRLYVKQWEDALTAQGAKIGIPYWDWTTA : 2632
KLH1        : --------------------------------------------------------------------------------------------------DGKNYSCCTHGMATFPHWHRLYTKQMEDALTAHGARVGLPYWDGTTA : 2614
HtH1        : --------------------------------------------------------------------------------------------------DGHNYSCCTHGMATFPHWHRLYTKQMEDAMRAHGSHVGLPYWDWTAA : 2615
OdH-G       : --------------------------------------------------------------------------------------------------NGTAYACCQHGMVTFPNWHRLLTKQMEDALVAKGSHVGIPYWDWTTT : 2621
NpH         : -----------------------------------------------------------------------------------------------------PYACCQHGMPTFPHWHRLYVKQMEDTLHTHGSHLGIPYWDWTEA : 2626
```

```
                  *    3060         *    3080         *    3100         *    3120         *    3140         *    3160         *    3180         *
NlH1       : FTELPTLVS-EELDNPFHHGHIPGKDKNITTTRAPRPQLFKDPEHGEESFFYRQALLTFEQKDFCDFEVQFEVLHNAIHSWIGGTSPYGMSTLEYCAYDPIFFIHHSNVDRQFAIWQELQKFRKLDYNTANCHIQDLRQPLEPFN : 2780
RtH1       : FTELPTLVS-EDHDNPFHHGHVPGKAENITTTRAPRPQLFKDPEHGEESFFYRQALLAFEQRDFCDFEVQFEVLHNAIHSWIGGTSPYGMSTLEYAAYDPLFFIHHSNVDRQFAIWQELQKHRGLDYNTANCHIQDLRKPLEPFN : 2779
NlH2       : FTELPSLIS-EEHDNPFHHFEIP-DKEHHVTTRAPRPQLFNDPEHGDESFFYRQVLIAFEQRDYCDFEVQFEVIHNAIHSWIGGTSPYGMSTLEYAAYDPVFFIHHSNVDRQFAIWQALQKYRGLDYNTANCNIHELREPLEPFN : 3108
RtH2       : FTELPRLIS-QEHDNPFHHFEIP-DKEHHVTTRAPRPQLFKDPEHGDESFFYRQVLIAFEQRDYCDFEVQFEVIHNAIHSWIGGTSPYGMSTLEYAAYDPVFFIHHSNVDRQFAIWQALQKYRGLDYNTANCNIHELREPLEPFN : 2887
LisaH1     : FTELPALVT-EEENNPFHHGVIE--ALNMTTSRAPRPQLFNDPEHGDESFFYRQVLLAFEQRDYCDFEVQFEVAHNAIHSWIGGTSPYGMSTLEYSAYDPVFFIHHSNVDRQFAIWQALQQYRGLDYNTANCNIQELRQTLEPFD : 2771
LisaH2     : FTDLPALFT-EKENNPFHHGIVP--GSGNKTTRAPRPQLFNDPEQGEESFFYRQILLAFEQRDYCNFEVQYEITHNALHSWIGGTSPYGMSTLEYAAYDPIFFIHHSNVDRQFAIWQALQKYRGLDYNTANCNIQELRQPLEPFD : 2764
MtH400     : FTALPSLVT-DDGDNPFNHGHIE--ALGKVTSREPRPQLFKDPEHGEESFFYRQILLAFEQRDYCDFEVQFEVTHNAPHSWIGGTSPYGMSTLEFSSYDPVFFLHHSNVDRQFAIWQALQKYRGLDYNSANCDIQELRMDLEPFN : 2785
MtH550_a-f : ---------------------------------------------------------------------------------------------------------------------------------------------- :    -
PcH_I      : FTNLPSLLTADDSSNPFLKGHIK--ALNQSTSRSPRPQLFNDPERGEESFFYRQILLAFEQRDYCDFEVQFEVTHNAIHSWIGGTSPYGMSTLEYSAYDPIFFIHHSNVDRQFAIWQALQRYRGLEHNSANCNIQELKMPLEPFN : 2763
PcH_IIb    : FTSLPTLLT-DKDDNPFEHGEIA--SLNEKTTRSPRPQLFSDPERGEESFFYRQILLAFEQRDYCNFEVQYEVTHNAIHSWIGGTSPYGMSTLEYSAYDPIFFIHHSNVDRQFAIWQALQRYRGLDYNSANCNIQELRQPLEPFN : 2768
PcH_III    : FTALPSLLT-ETENNPFSQGEVA--ALHQVTTRAPRPQLFSDPERGEESFFYRQILLAFEQRDYCDFEVQYEVTHNAIHSWIGGTSPYGMSTLEYSAYDPVFFIHHSNVDRQFAIWQALQRYRGLDYDSANCNIQELRLPLEPFN : 2765
LsH1       : FTALPTLVT-EETNNPFHHGKI---YNGEITTRAPRDQLFNDPEFGSESFFYRQALFTFEQTDYCNFEVQFEITHNAIHSWTGGSSQYGMSSLEYTAYDPLFLLHHSNVDRQFAIWQALQKFRGLPYNSANCAIQHLRQPMRPFS : 2777
LsH2       : FTALPTLVS-QEDDNPFHHGKI---YNGEVTTRAPRDKLFNDPEFGAESFFYRQVLLAFEQTDYCDFEVQFEITHNAIHSWTGGSSPYGMSTLEYTAYDPLFLLHHSNVDRQFAIWQALQKFRGLPYNSANCAIQHLRQPMRPFS : 2773
KLH1       : FTALPTFVT-DEEDNPFHHGHID--YLGVDTTRSPRDKLFNDPERGSESFFYRQVLLALEQTDFCQFEVQFEITHNAIHSWTGGLTPYGMSTLEYTTYDPLFWLHHANTDRIWAIWQALQEYRGLPYDHANCEIQAMKRPLRPFS : 2756
HtH1       : FTHLPTLVT-DTDNNPFQHGHID--YLNVSTTRSPRDMLFNDPEHGSESFFYRQVLLALEQTDFCKFEVQFEITHNAIHSWTGGHSPYGMSTLDFTAYDPLFWLHHSNTDRIWAVWQALQEYRGLPYNHANCEIQAMKTPLRPFS : 2757
OdH-G      : FANLPVLVT-EEKDNSFHHAHID--VANTDTTRSPRAQLFDDPEKGDKSFFYRQIALALEQTDFCDFEIQFEIGHNAIHSWVGGSSPYGMSTLHYTSYDPLFYLHHSNTDRIWSVWQALQKYRGLPYNTANCEINKLVKPLKPFN : 2763
NpH        : FEYLPILVT-ETEHNPFHHAKIE--FINETTSRNPRPQLFTDQRYGETSFFYRQILLALEQTDFCDFEIQFEVGHNAIHSWVGGSTQYGMSTLHFTSYDPLFYIHHSNTDRIWAIWQALQRYRGLPYNSANCEIHLLKKPLEPFS : 2768

                3200         *    3220         *    3240         *    3260         *    3280         *    3300         *    3320         *
NlH1       : RPDNPVLVTKAHSRAIDAFNYDQYGYQYDHLNFHGMSVEKLHHKLEKRQEQDRVFLNFMLHGIKMSGDVEFDLCNEKGTCNFAGTFAILGGPLEMPWQFDRVFKYDVTKIFRQMRLRPDSNYTIPLRIRAVNGMELDSHIMEPPS : 2925
RtH1       : RAGNPVLVTRAHSRAIDAFNYDQYGYQYDHLNFHGLSVQELEEKLHERAEQDRVFLNFMLRGIKMSADVEFDLCNAQGTCNFAGTFAILGGPLEMPWNFDRVFKYDVTKIFRQMRLRPDSNYTIPIRIRAVNGMQLDPDLLEPPS : 2924
NlH2       : HEDNPVHSTRSHSRAIDAFDYDQFDYQYDNLNFHGLTIPQLDHLLEEKKAEDRVFLNFMLKGFESSADVEFELCDDHDHCNFAGTFAVLGGPLEMPWTFDRTFKYDVTKVFQQMKLRPDSEYHFEIHIRAVNGTDLNPRLLKPPS : 3253
RtH2       : HEDNPVHITRAHSRAIDAFDYDDLDYQYDNLNFHGLTIAQLDHLLEQRKSEDRAFLNFMLQGIGTSADVEFELCDKDNHCNFAGTFAILGGPLEMPWSFDRVFKYDVTKVFKQMRLRPDSEYHFELHVRAVNGTELSPRLLKPPS : 3032
LisaH1     : SPHNPVSIIREHATAIDAFNYDQFSYQYDNLNFHGLTISQLDDLLEERKDEDRVFANFLLHNIGTSADVEFDLCDAEEHCQFAGTFAILGGPLEMAWRFDRLFKYDVTSVFRQMRLRPDSVYHFRVHLTAVNGTELDAHLIEDPS : 2916
LisaH2     : RDDNPIPTTRQHAHAIDAFNYDQFGYQYDNLNFHGLTIPELENLLEKKKRDDHIFVNFMLHGIHTSADVEFDLCNEEGTCNFAGTFAILGGPLEMPWSFDRLYKYDVTNVFKQMRLRPDSVYHFDVRIRAVNGTQLDAHLIEAPS : 2909
MtH400     : RDYIPIVIIRNNARAIDAFNYDQYSYQYDNLNFHGMSIPELEALLEKRRSEDHVFLNFMLLGIGASADVTFDICDSEGHCNFAGTFAVLGGPLEMPWSFDRLFKYDVTSVFRQMHLRPDSDYSVKVHIVSVNGTELDSDLLEAPS : 2930
MtH550_a-f : ---------------------------------------------------------------------------------------------------------------------------------------------- :    -
PcH_I      : RKENLITIIRENSRAIDAFNYEQFGYQYDNLNFHGLTIPELEAVLEARRQEDRVFANFMLHGIRSSADVSFDICDAQNHCIFAGTFAILGGPLEMPWVFDRLFKYDVTSVFKQLHLRPDSEYRFKMRLTAVNGTELDPHLLHAPS : 2908
PcH_IIb    : WDSNPNDFIRENARAVDAFDYDQFNYQYDDLNFHGLTIPQLEEAIEKRRSEDRVFANFMLQGFKASADVTFDLCDADNHCSFAGTFAVLGGPLEMDWHFDRLFQYDVTSVFNQMHLRPDSVYHFDVHITAINGTQLASSLLESPS : 2913
PcH_III    : WDDIPYPIIRENARAIDAFHYDRFNYQYDNLNFHGLTIPQLEAVLEKRRDEDRIFANFMLAGFGASADVTFELCDKENHCIFAGTFAILGGPLEMPWHWKFDRLFKYDVTAVFKQLHLRPDSEYHFDVHIRAVNGSELSSSLLEPPS : 2910
LsH1       : DENNLNAITRANSRAIDAFDYDRLNYQYDNLNFHGLTISQLNDLLEKRKEEDRIFAEFLLRGFSASADVIFKLCDEKGHCEFAGTFAVLGGLLEMPWAFDRLFKYDVTNVFNKLNLRTDSDYHFDVEIKAINGTVLDSNLIRSPS : 2922
LsH2       : DESNINAITRANSRAIDAFDYDRFNYQYDNLNFHGLTISQLNDLLEKRIEEDRIFAEFLLIGFGASADVTFQLCDAENHCAFAGTFAVLGGPLEMPWALDRLFKYDVTNVFNQLHLKPDSKYHFEVKIVAVNGTELDSTIIRPPT : 2918
KLH1       : DPINHNAFTHSNAKPTDVFEYSRFNFQYDNLRFHGMTIKKLEHELEKQKEEDRTFAAFLLHGIKKSADVSFDVCNHDGECHFAGTFAILGGEHEMPWSFDRLFRYDITQVLKQMHLEYDSDFTFHMRIIDTSGKQLPSDLIKMPT : 2901
HtH1       : DDINHNPVTKANAKPLDVFEYNRLSFQYDNLIFHGYSIPELDRVLEERKEEDRIFAAFLLSGIKRSADVVFDICQPEHECVFAGTFAILGGELEMPWSFDRLFRYDITKVMKQLHLRHDSDFTFRVKIVGTDDHELPSDSVKAPT : 2902
OdH-G      : LDTNPNAVTKAHSTGATSFDYHKLGYDYDNLNFHGMTIPELEEHLKEIQHEDRVFAGFLLRTIGQSADVNFDVCTKDGECTFGGTFCILGGEHEMFWAFDRLFKYDITTSLKHLRLDAHDDFDIKVTIKGIDGHVLSNKYLSPPT : 2908
NpH        : FESNPNHETHNHSTGNAVFDYHQLGYEYDTLEFHGHNIPQLAVYLDSYHEHDRVFAGFLLHGIKRSADVNFDICSLNGDCTNAGTFALLGGEHEMPWAFDRLFKYDITKPLQNLRLHSTDDFSINIHIVAINGEELDSHLIPTPS : 2913

         3340         *    3360         *    3380         *    3400         *    3420         *    3440         *    3460         *    3480
NlH1       : VTFVPGR-KVGSTKAVVHEDPMPTET-----EDLTRYEISALSLEQISNLRNALYKLQNDFGPNGFEAIASFHGAPGVCPQNASK-HHSCCQHGMPAFPHWHRLLTVQFEHALKEKGAGVGVPYWDWTRPSKGLPSFFTDPSNINP : 3064
RtH1       : VTFAPGK-KSGSGRAVVYEEPTPSET-----QDLTRHDLSSLSMAQISNLEDALYKLQNDHGPNGFEAIASYHGAPGLCPENATE-HYSCCQHGMPAFPHWHRLLTVQFEHALKEKGAMVGVPYWDWTRPAKGMPTLFTDNSAINP : 3063
NlH2       : VSFLPGR-KTYSEVAEDHDEHTPTES-----DDMVRYEVSDLSLAQVSNLRDALYKLMNDHGPNGFESIASFHGAPGLCPENATE-HYACCQHGMSAFPHWHTLLTVQFEHALHDKGALVGIPYWDWTRPSKSLPSLFTDPSEGNS : 3392
RtH2       : VSFLPGR-KTRGQAADAHHEEQPTNTTTDSDDDLVRYEVSAMSLAQVNNLRTALNKLMHDSGPNGYEAIAGFHGAPGLCPEKAEE-HYSCCQHGMPAFPHWHSLLTVQFEHALREHGSLVGVPYWDWTRPSKALPSLFTDPSESNP : 3176
LisaH1     : VSFLPGR-DHGVSHAQAHEDPIPLAS-----EDVTRYEVSSLSLEQSSNLRNALYKLQNDDGPNGFEAIASFHGAPGLCPENGGKLRIACCRHGMPAFPHWHRLLTVQFERALRDKGAVVGVPYWDWTRPVKALPSLLTDPYDNNP : 3056
LisaH2     : VSFVPGK-KPLGSRVVVEEDPTPTET-----DDVTRFEVSSLTLEQVSNLRNALYKLQNDFGPNGFEAIASFHGSPGLCPENATE-RYACCRHGMPAFPHWHRLLTVQFERALRDQGAVVGVPYWDWTRPAKALPSLFTDPFDNNP : 3048
MtH400     : VSFVPGK-RGRKAREDYRPTVS---------EDLIRKEVSSLSLEEVSNLKNALYKLQNDHGPNGFEAIASFHGDPGLCPEGSGS-KYACCQHGMPVFPHWHRLLTVQFERALKAKGSVVGVPYWDWTRPSRGIPQLFSNTYGNNP : 3065
MtH550_a-f : ---------------------------------------------------------------------------------------------------------------------------------------------- :    -
PcH_I      : VSFLPGR-GEQRARA-AREDPVTTVS-----SVVTRYDVDTLTLEQASSLRNALHKLQNDHGLTSYEAIASFHAGSGLCPENASV-TFACVPHGFANLPHFNRLLLVQMEMALLEKGATTGIPYWDWTRTIRALPSLVAE-SGDNS : 3045
PcH_IIb    : VSFVPGK-GKTGSRAVAKEDPQPTKT-----KDVVRYEVSALSLEQVSNLRNALYKLQNDDGPNGYEAIASFHGAPKLCPENATD-RYACCTHGMPVFPHWHRLLTVQFEMALRDKGALLGIPYWDWTRPSKALPSLLTS-TDNNP : 3051
PcH_III    : VSFVPGR-RR-GAHAASSEEPQPSPS-----DDLVRYEISALSLEQVSNLKNALYKLQRDDGPNGFETIAGFHGYPNNCPENTTN-RYACCTHGMPVFPHWHRLLTVQFEMALKDQGARVGVPYWDWTRPAKNLPSLLTD-TKENP : 3047
LsH1       : VQFVPGVKRFYEKVAEKTVVRD---------DKLVRKNVNQLTLDEAANLRNALNQLQNDQGPNGFEAIAGFHGAPFRCPATGDD-KYACCVHGMPVFPHWHRLFTVQIEQALKSNGALIGVPYWDWTTPVKSLPSFFGDDADHNP : 3058
LsH2       : VQFVPGVKRYYEKLAERTVHRE---------DKLTRKDVNTLTLAEATNLRNALSQLQQDAGLNGFEAIAGFHGAPFKCPATGTD-KYACCVHGMPIFPHWHRLLTVQFEQALKAHGASVGVPYWDWTAPVRSLPSLFGDSADFNP : 3054
KLH1       : VEHSPGG-KHHEKHHEDHHE-----------DILVRKNIHSLSHHEAEELRDALYKLQNDESHGGYEHIAGFHGYPNLCPEKGDE-KYPCCVHGMSIFPHWHRLHTIQFERALKKHGSHLGIPYWDWTQTISSLPTFFADSGNNNP : 3034
HtH1       : IEFEPGV-HRGGNHEDEHHDDRLA-------DVLIRKEVDFLSLQEANAIKDALYKLQNDDSKGGFEAIAGYHGYPNMCPERGTD-KYPCCVHGMPVFPHWHRLHTIQMERALKNHGSPMGIPYWDWTKKMSSLPSFFGDSSNNNP : 3039
OdH-G      : VFLAPAKTTH------------------------------------------------------------------------------------------------------------------------------------ : 2918
NpH        : IIFSPGRGAQH----------------------------------------------------------------------------------------------------------------------------------- : 2924
```

```
                      *      3500         *       3520         *       3540         *       3560         *       3580         *       3600         *       3620
NlH1       : FKTYLMSFNDHHIERQVHEDLFSHSSEAD--PESLFHQALEMLEETNYCDFEVHYEMLHNAVHELIGGNHTYSMSTIEYSAFDPFFMVHHSSIDRIWAIWQTLQKLRHKPFNYAVCAA-RSMNRPLEPFSYDTINKDSLTHDNAM : 3206
RtH1       : FYTYLMSFNNHHIERKVHEDLFSHSAEGD--PESLFHQALELLEETNYCDFEVQYEMLHNAVHELVGGPHKYSMSTIEYSAFDPFFMVHHSSIDRIWQIWQTLQKLRHKPFNYAVCAA-RSMYKPLEPFSYESINPDPLTRENSK : 3205
NlH2       : FRTYKISFNNQYIQREVQDELFNHPSEGD--PESLFHQALELLEETSYCDFEVQYEMLHNAVHELVGGRHTHGMSTIEFSAFDPLFMVHHASIDRVWKIWQELQKLRHKPFNYAVCAH-RNLYRHLEPFSYETVNTDALTRNNAQ : 3534
RtH2       : FKTYKISFKGQYIKREVHEDLFNHPSEGD--PESLFHQALETLEQTSYCDFEVQYEMLHNAVHELVGGPNTYSMSTIDYSAFDPFFMVHHASIDRLWLIWQQLQKLRGKPFNYAVCAA-RLLYRPLEPFSYESVNADPLTRNNAK : 3318
LisaH1     : FRSYRISFVEQYTSRDVQAELFKHPSEGD--LESLFHQALETLEENNYCDFEVQYEMLHNAVHELVGGSRKYGMSTIEYSAFDPFFMVHHSSIDRLWLIWQQLQKLRGKPFNFARCAG-RSLFKPMEPFAYESVNADPITRANAE : 3198
LisaH2     : YRTYRISFNEQYVQRDIQSEMFVHSAEGD--HESLFYQALETLEESNFCDFEVHYEMLHNAVHELVGGTKTYGMSTIEYSAFDPFFMVHHSSIDRIWQIWQALQKLRRKPFNYARCAH-RNLFKHLEPFAYDSVNADPLTRANSQ : 3190
MtH400     : FLTYEIQAASAFVERDVQVDALEHTTGHEGFEDSLFHQAALETLEETSYCEFEVQFEMLHNAVHALVGGTKTHSMATLEWSAFDPFFMVHHSSIDRIWRIWQELQRLRKKPFNVARCAM-RYLRKPLEPFSYASVNTDEVARTNSR : 3209
MtH550_a-f : -------------------------------------------------------------------------------------------------------------------------------------------------- :    -
PcH_I      : FFSYHIRQANKDTVRDPQEDLYVASR-GT--RNILFEQTLLALEEVNFCDFLVQMDLLHVRVHALVGGKEAFSMATMEHAAFDPLFWLHAANVDRLWQAWQELQKLRRRSYHSGSCAR-VTEDTPMLPFSSETLNPNPVTHANAR : 3186
PcH_IIb    : FSTYQIKFAQHQVTRDVQPGLYNRRKQSG--LDSLFYQTLETLEETNYCDFEVQFEMLHNTIHALVGGQGTYSMSTIEYSAFDPFFMIHHSSIDRIWQIWQTLQKLRHRSFNYARCAL-RNLYRPLEPFNYESKNPNPVTRANSR : 3193
PcH_III    : FSGYQIKSASQQVTRDVQQLLFNRTQHGS--QDYLFDQTLETLEEDTYCDFEVQLEMLHNTLHALVGGNGTYSMATLDYSAFDPYFMIHHSSIDRIWKIWQTLQKLRHRPFNVARCAA-RSLFRPLEPFSYE-LNPNPITRANSK : 3188
LsH1       : FASFRIAAANEKTTRQVQSELYSERKVHG--FPYLFYLALTTLEEDNYCDFEVQFEVLHNEIHADIGGSGTYSMATLDYSAFDPFFMIHHSSIDRIWVIWQELQKLRHKPFNGASCAG-HIMERPLQPFSYPEVNKNEFTRLNSV : 3200
LsH2       : FYSYFISFVGKKTTRDVKDELFNPPTING--FNQLYLALSTLEEDNYCDFEVQYEILHNQIHALIGGTGTYSMATLDYSAFDPFFMIHHSSIDRIWVIWQELQKIRKKPFNAAHCGG-HLMETPLQPFSYPEVNPNDLTRLNAV : 3196
KLH1       : FFKYHIRSINQDTVRDVNEAIFQQTKFGE--FSSIFYLALQALEEDNYCDFEVQYEILHNEVHALIGGAEKYSMSTIEYSAFDPYFMIHHASLDKIWIIWQELQKIRRVKPAHAGSCAG-DIMHVPLHPFNYESVNNDDFTRENSL : 3176
HtH1       : FYKYYIRGVQHETTRDINQRLFNQTKFGE--FDYLYYLTLQVLEENSYCDFEVQYEILHNAVHSWLGGTGKYSMSTIEHSAFDPVFMIHHSSLDRIWILWQKLQKIRMKPYYALDCAGDRLMKDPLHPFNYETVNEDEFTRINSF : 3182
OdH-G      : -------------------------------------------------------------------------------------------------------------------------------------------------- :    -
NpH        : -------------------------------------------------------------------------------------------------------------------------------------------------- :    -

                  *      3640         *       3660         *       3680         *       3700         *       3720         *       3740         *       3760         *
NlH1       : PSQIFDSHKFHYHYDNLQLNGHSVTELYKMIHSMQAQTRTFAGFVLSGFGSSARVHVDIVKGAD---TASVGNFFVLGGPTELPWAYERIYKLDMTEATKKLGLAGSTSFEFKLTVTKYDGTPMDVH-FPDPVIKRRANSHFDE : 3347
RtH1       : PAQIFDTHKFHYHYDNLNLNGHSVAELNTMIHAMQAQSRTFAGFVLSGISTSARVHVDIYKGDE---VASVGNFFVLGGPSEMPWAYERIYKLDMTEAAKKLGLSGSSHFDFKLTVTKYDGSALDVK-FPDPVIKRRTNAEFDE : 3346
NlH2       : PVQIFDKTKFHYHYDNLELNGHDLHELEEMIHEMQTHTRVFAGFVLYGFGTSARVHVDIMKDGE---EVKVGNFFVLGGPSEMPWAYERIYKLDMTDAVHELNQTENCDFDFQLTVTKYDGTQLDVV-FPEPVIIKRPNVESDE : 3675
RtH2       : PVQIFDREKFHYRYDSLELSGHALQELEDMIHDMQTHTRLFAGFVLHGFGTSARVHVDIVKDGH---VEKVGNFFVLGGASEMPWAYERIYKLDMTEAAEHLNMSDSCAFHFQLTVTKYDGTALDVE-FPEPVVIRKRANTEFEE : 3459
LisaH1     : PVHVFDTEKFHYHYDSLELNGHSVKQLQEMVDSMRNSPRVFAGFLLHGVGTSASVHVDVVAADGS--KVNAGNFYVLGGEEEMPWAYERPYKLDITDAISKAGLAHGDTVHLSTSVARYDGTPLNVT-FPSSLIIERPANADYDV : 3340
LisaH2     : PVQIFDAAKFHYNYDSLSLNGHSVAELKEMIASMKANTRLYAGFVLSGIGTSARVQVDIDSESGE--HVNVGSFYILGGDGEMPWAYERIFKLDMTDAANHLGLGPDSNFDFHLNVTKYDGTHLDVN-FPAPVVVKRAANAEYDV : 3332
MtH400     : PIDIFDTAKFHYDFDNLDLGGHSIGEVNKMINDMRSKTRLFVGFVLSGIETSATVKIEMDDSQGN--SHEVGTFYVLGGHNEMPWAYERVYKYEITEAAKKYGLDHDSVFNFHHKVVKYDGQELSAQ-FKAPILVERPAGVDYDV : 3351
MtH550_a-f : -------------------------------------------------------------------------------------------------------------------------------------------------- :    -
PcH_I      : PVQSVEVDKFRYSYDHLDFNRRSVSELLEATQSLRVKDRLFAAFLLSGVHTSARLHVTLQTGSDE-GAVEVGSIYLLGGLQERRWAHERAYKLDVTEAAARLELDPYSTFDFNVSLFDYTGQPLPYT-LPYPLVLYRPASVDFDV : 3329
PcH_IIb    : PVQIFDASKFHYNYDNLNLNGHSVSEINTIIENLRDHDRVYAGFVLSGIGVSATANVKLVPQGGD--PVDVGSFYILGGDGEMPWAYERVYKYDVTDALEKLGLNAYSNFGFQVTLTKYNGEQLDAS-LATPVVISRPANADYDV : 3335
PcH_III    : PIQIFDAAKFHYEFDNLNLNGHSVEEIDTLIKNLRAKDRVFAGLVLSGFSTSATAHVEVGVSGKS--TQDEGSFYILGGPKELPWAYERVYKYDITHAVTELGVSPYSPIDITVTLTGYDGSSVDAS-LPSPLVVVRPANADYDV : 3330
LsH1       : PNVVFDSERLGYKYDKLELNGNSVEEINNIIKNLHHQERIFVGILAFGHQKSLTIHISLINDNDE--AFDGGNIHILGGEKEMPWAYERIMKLDVTEAIRKGKISTDHAVKARFTSTDYQGNLNHQD-TDYAIIVERHAEQDYDV : 3342
LsH2       : PNLVFDSERFGYEFDKLELNGHDVSELNGIIQRLRGNTRYFLGFVLYGVRQTVGVEVTLISNKK---RYPAGNFYVLGGEKEMPWAYERIAKYDVTDVFHKAKLSDDKPVVGEFYSSLYNGQANNNV-TEEVIVVEKPSDVNYDI : 3337
KLH1       : PNAVVDSHRFNYKYDNLNLHGHNIEELEEVLRSLRLKSRVFAGFVLSGIRTTAVVKVYIKSGTDSD-DEYAGSFVILGGAKEMPWAYERLYRFDITETVHNLNLTDDH-VKFRFDLKKYDHTELDASVLPAPIIVRRPNNAVFDI : 3319
HtH1       : PSILFDHYRFNYEYDNMRIRGQDIHELEEVIQELRNKDRIFAGFVLSGLRISATVKVFIHSKNDTSHEEYAGEFAVLGGEKEMPWAYERMLKLDISDAVHKLHVKDED-IRFRVVVTAYNGDVVTTR-LSQPFIVHRPAHVAHDI : 3325
OdH-G      : -------------------------------------------------------------------------------------------------------------------------------------------------- :    -
NpH        : -------------------------------------------------------------------------------------------------------------------------------------------------- :    -

                 3780         *       3800         *       3820         *       3840         *       3860         *
NlH1       : LILPIRKE-NKLPPKIVVRRGTRVLFHPTEVSVVG-PIRELGSFTNSEHCAIPPGHAHSYELDVLHVLEPGDYYFVSNNL--DAC-KAGSRVQISVDEE-- : 3441
RtH1       : LILPIRKE-NKLPPKIVVRRGTRVLFHPTEEGILG-PIRELGSFTNSKLCAIPPGHAHAYELDRLHVLEPGDYYFVSNNV--ESC-KAGSRIQISVDEE-- : 3440
NlH2       : LILPLRMV-NRLPPKIVVRRGTRVIFHPLEDEVKG-PLRELGSYTSSLHCTIPPGEAHAYDFDVPHDLEPGDYYFTVNDE--SQC-EGGARFQITVDEE-- : 3769
RtH2       : VILPLKTK-NNLPPKIVVRRGTHVIFHAVDQALHG-PIRELGSYTNSVHCAIPPGEAHAYAFDESHELEPGDYYFTANDA--QQC-HGGARIQLTVDEE-- : 3553
LisaH1     : LVVQFGAE-QAKDYKVRVKRGTRVRFHSTLEGYVG-QVKELGTYTNSQLCVIPPGLANSYELEMEHKLEPGDYFFTDFDR--SGC-LAGHRIMISVDQDWN : 3436
LisaH2     : LILPLKKV-NDLPPKIVVRRGTRVLFHVTDKEISG-PVRELGSYTNSVHCAIPPGEANAYDLDVPYVLQPGDYYFTSNVV--EQC-KAGSRIQISVDDE-- : 3426
MtH400     : AIFQLKKD-NDLAPKVVLPRGTRIRFHSAEDGVSS-VLREMGSYTNAVYCSIPPGDANTYKLEVEYTLEPGDYFFVSNDE--EKC-KQGDRIQISIDEE-- : 3445
MtH550_a-f : --------------------------------------------------------------------------------------------------- :    -
PcH_I      : LVYPLYVD-KALPPKVTVRRGTKIRFHAADPSLQGRRIRTFGSYTLFIKCEALPGNADALSLDVTYSLNPGEYYLALDGDLPGKC-LEAGRTILVIDEE-- : 3426
PcH_IIb    : LILPLLEE-NKLPPKVIVSRGTRVRFHSPVSSLNA-NVKEVGSYTSLSLCSIPPGDANSYDPDVNYSLEPGDYFFVSSNK--ARC-EQGTRVQISIDDE-- : 3429
PcH_III    : LVVPLETA-VSIPPKVIVRRGTQIRFVGGGESVQG-RVRELGSYTTLQKCSIPPGDANVYEYNINNALQPGDYYFTLDNA--ESC-KGGKRIQITVDEE-- : 3424
LsH1       : VEIPVGRK-YPLPPKIVLKKGSRVKFYPGSDTFNS-PLENLGSYTSFSKCSIPPFSYQSYALGVVHTLQPGDYFFVPKDK--ALC-EAGKRIQITVEDE-- : 3436
LsH2       : VIIPISTG-NIIPPKIILKKGSRVKFVSSNISV---PMEELGSYTNLQKCKVPPFSFHSYPFGVVHTLAPGDYFFVPKSV--QLC-EAGRRIQITVEDE-- : 3429
KLH1       : IEIPIGKD-VNLPPKVVVKRGTKIMFMSVDEAVTT-PMLNLGSYTAMFKCKVPPFSFHAFELGKMYSVESGDYFMTASTT--ELCNDNNLRIHVHVDDE-- : 3414
HtH1       : LVIPVGAG-HDLPPKVVVKSGTKVEFTPIDSSVNK-AMVELGSYTAMAKCIVPPFSYHGFELDKVYSVDHGDYYIAAGTH--ALC-EQNLRLHIHVEHE-- : 3419
OdH-G      : --------------------------------------------------------------------------------------------------- :    -
NpH        : --------------------------------------------------------------------------------------------------- :    -
```
