# Supplementary material for: The Evolution of Hemocyanin Genes in Caenogastropoda: Gene Duplications and Intron Accumulation in Highly Diverse Gastropods
Source: J Mol Evol. 2021 Nov 10;89(9-10):639–55. doi: 10.1007/s00239-021-10036-y (PMC8599328; doi:10.1007/s00239-021-10036-y)

Supplement 3: Gene structures of FU-f of MtH_{400} and FU-f, $FU\text{-}f_1$ – $FU\text{-}f_6$ of MtH_{550}. Shown are coding sequences of hemocyanin functional units (typical and additional FU-f) of *M. tuberculata* (big boxes: coding for functional units; small and grey boxes: coding for linker peptides) and intron positions within their genes represented by arrows. The comparison of exon-intron structures of the represented hemocyanin functional units indicates that all additional functional units differ in their gene structure with exception of $FU\text{-}f_5$ and $FU\text{-}f_6$. The latter ones, however, share internal introns located at the same position with respect to their coding sequences. The additional FUs include two to six internal introns each. $FU\text{-}f_1$ – $FU\text{-}f_2$ of MtH_{550} are not separated by a linker intron (highlighted with a star in contrast to the yellow arrows symbolizing linker introns).

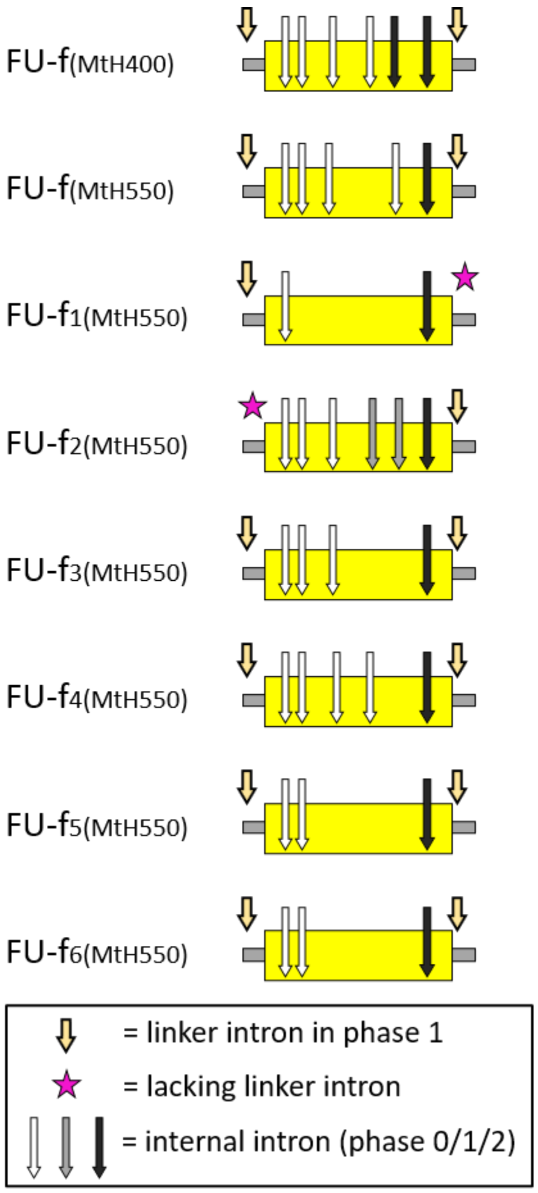

Supplement: Supplementary file 3 — Supplementary file3 (PDF 145 kb) [file 239_2021_10036_MOESM3_ESM.pdf]
